# Locoregional delivery of IL-13Rα2-targeting CAR-T cells in recurrent high-grade glioma: a phase 1 trial

**DOI:** 10.1038/s41591-024-02875-1

**Published:** 2024-03-07

**Authors:** Christine E. Brown, Jonathan C. Hibbard, Darya Alizadeh, M. Suzette Blanchard, Heini M. Natri, Dongrui Wang, Julie R. Ostberg, Brenda Aguilar, Jamie R. Wagner, Jinny A. Paul, Renate Starr, Robyn A. Wong, Wuyang Chen, Noah Shulkin, Maryam Aftabizadeh, Aleksandr Filippov, Ammar Chaudhry, Julie A. Ressler, Julie Kilpatrick, Paige Myers-McNamara, Mike Chen, Leo D. Wang, Russell C. Rockne, Joseph Georges, Jana Portnow, Michael E. Barish, Massimo D’Apuzzo, Nicholas E. Banovich, Stephen J. Forman, Behnam Badie

**Affiliations:** 1 Department of Hematology & Hematopoietic Cell Transplantation (T Cell Therapeutics Research Laboratories), City of Hope Beckman Research Institute and Medical Center, Duarte, CA USA; 2 Department of Computational and Quantitative Medicine, City of Hope Beckman Research Institute and Medical Center, Duarte, CA USA; 3 The Translational Genomics Research Institute, Phoenix, AZ USA; 4 Department of Neurosurgery, City of Hope Beckman Research Institute and Medical Center, Duarte, CA USA; 5 Department of Diagnostic Radiology, City of Hope Beckman Research Institute and Medical Center, Duarte, CA USA; 6 Department of Clinical Research, City of Hope Beckman Research Institute and Medical Center, Duarte, CA USA; 7 Departments of Immuno-Oncology and Pediatrics, City of Hope Beckman Research Institute and Medical Center, Duarte, CA USA; 8 Department of Medical Oncology, City of Hope Beckman Research Institute and Medical Center, Duarte, CA USA; 9 Department of Stem Cell Biology & Regenerative Medicine, City of Hope Beckman Research Institute and Medical Center, Duarte, CA USA; 10 Department of Pathology, City of Hope Beckman Research Institute and Medical Center, Duarte, CA USA; 11 Present Address: Bone Marrow Transplantation Center, the First Affiliated Hospital, and Liangzhu Laboratory, Zhejiang University School of Medicine, Hangzhou, China

**Keywords:** Cancer immunotherapy, CNS cancer, Immunotherapy

## Abstract

Chimeric antigen receptor T cell (CAR-T) therapy is an emerging strategy to improve treatment outcomes for recurrent high-grade glioma, a cancer that responds poorly to current therapies. Here we report a completed phase I trial evaluating IL-13Rα2-targeted CAR-T cells in 65 patients with recurrent high-grade glioma, the majority being recurrent glioblastoma (rGBM). Primary objectives were safety and feasibility, maximum tolerated dose/maximum feasible dose and a recommended phase 2 dose plan. Secondary objectives included overall survival, disease response, cytokine dynamics and tumor immune contexture biomarkers. This trial evolved to evaluate three routes of locoregional T cell administration (intratumoral (ICT), intraventricular (ICV) and dual ICT/ICV) and two manufacturing platforms, culminating in arm 5, which utilized dual ICT/ICV delivery and an optimized manufacturing process. Locoregional CAR-T cell administration was feasible and well tolerated, and as there were no dose-limiting toxicities across all arms, a maximum tolerated dose was not determined. Probable treatment-related grade 3+ toxicities were one grade 3 encephalopathy and one grade 3 ataxia. A clinical maximum feasible dose of 200 × 10^6^ CAR-T cells per infusion cycle was achieved for arm 5; however, other arms either did not test or achieve this dose due to manufacturing feasibility. A recommended phase 2 dose will be refined in future studies based on data from this trial. Stable disease or better was achieved in 50% (29/58) of patients, with two partial responses, one complete response and a second complete response after additional CAR-T cycles off protocol. For rGBM, median overall survival for all patients was 7.7 months and for arm 5 was 10.2 months. Central nervous system increases in inflammatory cytokines, including IFNγ, CXCL9 and CXCL10, were associated with CAR-T cell administration and bioactivity. Pretreatment intratumoral CD3 T cell levels were positively associated with survival. These findings demonstrate that locoregional IL-13Rα2-targeted CAR-T therapy is safe with promising clinical activity in a subset of patients. ClinicalTrials.gov Identifier: NCT02208362.

## Abstract (web summary)

In a large trial of patients with recurrent high-grade gliomas, IL-13Rα2-targeting CAR-T cells were feasible to manufacture and well tolerated when delivered via intratumoral and/or intraventricular routes.

## Main

Diffuse high-grade glioma (HGG) is an intractable cancer that responds poorly to standard-of-care (SOC) therapy of maximal surgical resection, focal radiation and chemotherapy^1^. Glioblastoma (GBM), defined as an isocitrate dehydrogenase (IDH)-wildtype World Health Organization (WHO) grade 4 astrocytoma, is the most aggressive type having the worst prognosis of HGGs. Despite intensive SOC treatment, tumor recurrence is inevitable and remains uniformly lethal with no current effective treatments^2,3^.

Chimeric antigen receptor T cell (CAR-T) therapy is being explored in early-stage clinical trials as a strategy to improve treatment outcomes for HGG. So far, the feasibility and safety of CAR-T therapy targeting a range of tumor-associated antigens in gliomas, including IL-13Rα2 (refs. ^4–6^), HER2 (refs. ^7,8^), EGFRvIII (refs. ^9,10^), GD2 (ref. ^11^) and B7H3 (ref. ^12^), have been reported. Encouragingly, a subset of patients in many of these early clinical trials have reported improved quality-of-life (QOL), objective responses, and noteworthy survival benefit. One case report from our institution demonstrated that locoregional delivery of IL-13Rα2-CAR-T cells mediated a complete response (CR) in a patient with multifocal recurrent GBM (rGBM)^6,13^. While these initial findings are encouraging, larger-scale clinical studies with more comprehensive correlative analyses are needed to better understand determinants of both antitumor potency and tumor resistance.

IL-13Rα2 is a cancer-testis antigen that is expressed by the majority of HGG, and in GBM is associated with a mesenchymal gene signature and poor prognosis^14,15^. The potential of IL-13Rα2 as a CAR-T target is further strengthened by the absence of expression in the normal brain tissue. Our group has optimized an IL-13 cytokine-directed CAR mutated at a single site (E12Y) and incorporating a 4-1BB costimulatory domain, which demonstrates preferential recognition of the intended target, IL-13Rα2, over IL-13Rα1, a low-affinity receptor that is more ubiquitously expressed^16,17^. In this Article, we report findings from a phase I trial of IL-13Rα2-targeted CAR-T cells for rGBM and other HGGs, representing the largest clinical study completed so far.

## Results

### Trial design and patient characteristics

We conducted a single-center, nonrandomized, five-arm, dose-escalation phase I study to evaluate memory-enriched IL-13Rα2-CAR-T cells for recurrent HGG (rHGG). This trial enrolled heavily pretreated patients with no enrollment restrictions for tumor size, multifocal disease, prior bevacizumab or number of recurrences, with approximately 75% of participants being treated following second recurrence or later and the majority being IDH-wildtype rGBM (41 of 58 response evaluable patients) (Table 1)^18^. Trial eligibility criteria included confirmed IL-13Rα2 tumor expression, Karnofsky Performance Score (KPS) ≥60 and life expectancy >4 weeks.

**Table 1:** Characteristics of patients that were evaluable for survival

	All *N* = 57	Arms 1 and 2 (ICT) *N* = 19	Arm 3 (ICV) *N* = 10	Arm 4 (Dual) *N* = 8	Arms 1–4 (Tcm) *N* = 37	Arm 5 (Dual Tn/mem) *N* = 20
**Age** (years at surgery) median (minimum, maximum)	49 (16*, 71)	50 (32, 71)	56 (16, 69)	58 (30, 65)	56 (16, 71)	49 (25, 65)
**Male** No. (%)	36 (63)	13 (68)	8 (80)	5(63)	26 (70)	10 (50)
**Histology** (at surgery)^ No. (%)						
Grade 4 GBM IDH wildtype	41 (72)	14 (74)	7 (70)	6 (75)	27 (73)	14 (70)
Grade 4 Diffuse midline glioma, H3 K27-altered	2 (4)	0	1 (10)	1 (13)	2 (5)	0 (0)
Grade 4 Astrocytoma, IDH-mutated	6 (11)	3 (16)	1 (10)	0	4 (11)	2 (10)
Grade 4 Diffuse astrocytoma, NOS	1 (2)	1 (5)	0	0	1 (3)	0 (0)
Grade 3 Glioma	7 (12)	1 (5)	1 (10)	1 (13)	3 (8)	4 (20)
**MGMTMG** No. (%)						
Methylated	21 (37)	7 (37)	4 (40)	1 (13)	12 (32)	9 (45)
Unmethylated	30 (53)	8 (42)	5 (50)	7 (88)	20 (54)	10 (50)
Unknown or not tested	6 (11)	4 (21)	1 (10)	0 (0)	5 (14)	1 (5)
**IL-13Rα2 H score** median (minimum, maximum)						
At screening	150 (50, 230)	180 (50, 210)	155 (50, 210)	145 (90, 230)	160 (50, 230)	133 (80, 210)
At surgery**	90 (10, 270)	140 (30, 230)	100 (10, 190)	65 (20, 160)	100 (10, 230)	55 (10, 270)
**CD3** (at surgery) median (minimum, maximum)	2 (1, 4)	2 (1,4)	1 (1, 3)	2 (1, 2)	2 (1, 4)	2 (1, 4)
**Multifocal** No. (%)	17 (30)	3 (16)	6 (60)	2 (25)	11 (30)	6 (30)
**Resection** No. (%)	49 (86)	16 (84)	9 (90)	7 (88)	32 (86)	17 (85)
**Tumor Volume** mm^3^ median (minimum, maximum)						
Presurgery	21,078 (873, 123,000)	31,910 (6,423, 123,000)	17,595 (6,906, 32,020)	16,570 (873, 63,280)	22,640 (873, 123,000)	13,630 (1,254, 37,716)
Pre-CAR-T cells	10,818 (648, 74,220)	19,220 (1,992, 74,220)	9,956 (1,041, 34,810)	10,284 (1,074, 48,130)	13,160 (1,041, 74,220)	6,860 (648, 48,867)
**Prior bevacizumab** No. (%)	18 (32)	8 (42)	3 (30)	4 (50)	15 (41)	3 (15)
**KPS** (pre-CAR-T cells) median (minimum, maximum)	80 (60, 100)	80 (60, 90)	75 (60, 90)	80 (60, 100)	80 (60, 100)	90 (80, 100)
**EROTC QLQ-C30 Score** (presurgery) median (minimum, maximum)	80 (45, 100)	80 (54, 100)	84 (69, 97)	77 (61, 98)	80 (54, 100)	79 (45, 96)
**Recurrence** No. (%)						
First	14 (25)	5 (26)	1 (10)	3 (38)	9 (24)	5 (25)
Second	23 (40)	5 (26)	6 (60)	3 (38)	14 (38)	9 (45)
Third or more	20 (35)	9 (47)	3 (30)	2 (25)	14 (38)	6 (30)
**OS** Months median (95% CI)	8.0 (6.2, 10.1)	8.0 (5.8, 16.3)	8.2 (6.5, NA)	4.5 (3.4, NA)	7.5 (5.8, 10.1)	10.2 (7.5, 19.9)
**rGBM Patient^^ OS** Months median (95% CI)	7.7 (6.0, 10.1)	6.9 (4.8, 12.2)	7.5 (4.2, NA)	4.5 (3.4, NA)	6.1 (4.8, 9.5)	10.2 (7.7, NA)

^*^Only one participant, UPN228, was <18 years of age.

^Histology at surgery by UPN provided in Supplementary Table 1.

^**^The only exception being UPN156: IL-13Rα2 H score at enrollment (100) was used since an at surgery value was not available.

^^^^For rGBM sample size, reference no. (%) of grade 4 GBM IDH-wildtype histology at surgery (fourth row), the exception being arm 5 with a sample size of *n* = 13.

NA, infinity.

**Fig. 1:**
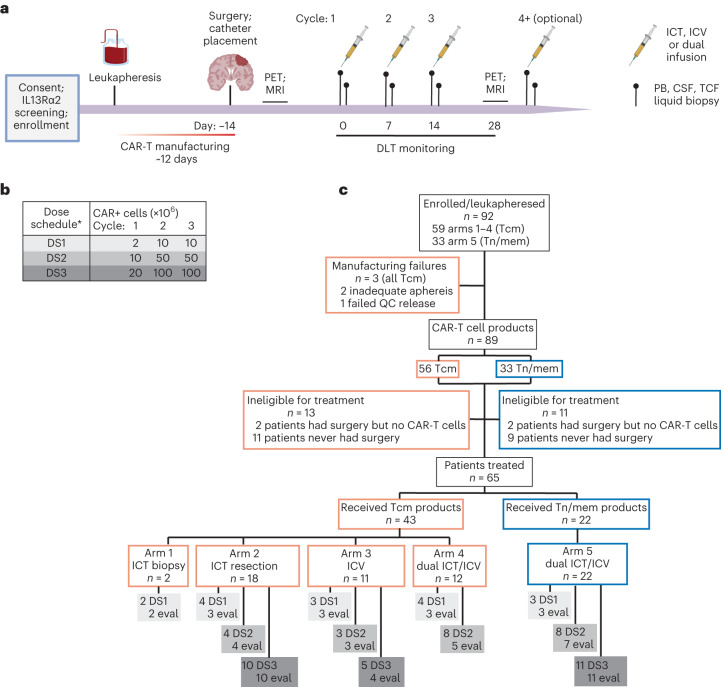
Study overview. **a**, Schema of patient treatment (created with BioRender.com). **b**, Schema of dose escalation schedules (DS). *Participants on dual ICT/ICV arms (arms 4 and 5) received the indicated number of CAR+ cells at each site. **c**, Consort diagram of patient enrollment and treatment on each arm and dose schedule (DS1, DS2 or DS3). Tcm, central memory T cells; Tn/mem, naive, stem cell memory and central memory T cells; QC, quality control; ICT, intratumoral; ICV, intraventricular; eval, evaluable for response.

Patients were treated at one of three dose schedules of weekly infusions and evaluated for 1 week after the third cycle for dose-limiting toxicities (DLTs) (Fig. 1a,b). Additional infusions were allowed, and patients were followed for toxicities, response and survival until they progressed or required disallowed therapy. After patients went off protocol therapy, they were only followed for toxicities and survival, and not disease-response, since other therapies were allowed^19^, except for unique patient number (UPN) 109 who was also followed for anti-tumor response on a single-subject protocol (SSP) under NCT02208362 (SSP available in ref. ^6^).

This study evolved to evaluate five treatment arms testing three routes of locoregional administration and two manufacturing platforms (Fig. 1c). Delivery routes included the following: arm 1, intratumoral following biopsy (ICT-Biopsy); arm 2, intratumoral following maximal surgical resection (ICT-Resection); arm 3, intraventricular (ICV); and arms 4 and 5, ICT and ICV delivery (Dual ICT/ICV). In addition, two manufacturing platforms (Supplementary Fig. 1) were utilized in this trial differing in T cell subsets enriched for CAR engineering: arms 1–4 utilized CD62L+, CD45RA− central memory T cells (Tcm); and arm 5 utilized CD62L+ enriched naive, stem cell memory and central memory T cells (Tn/mem).

**Extended Data Fig. 1:**
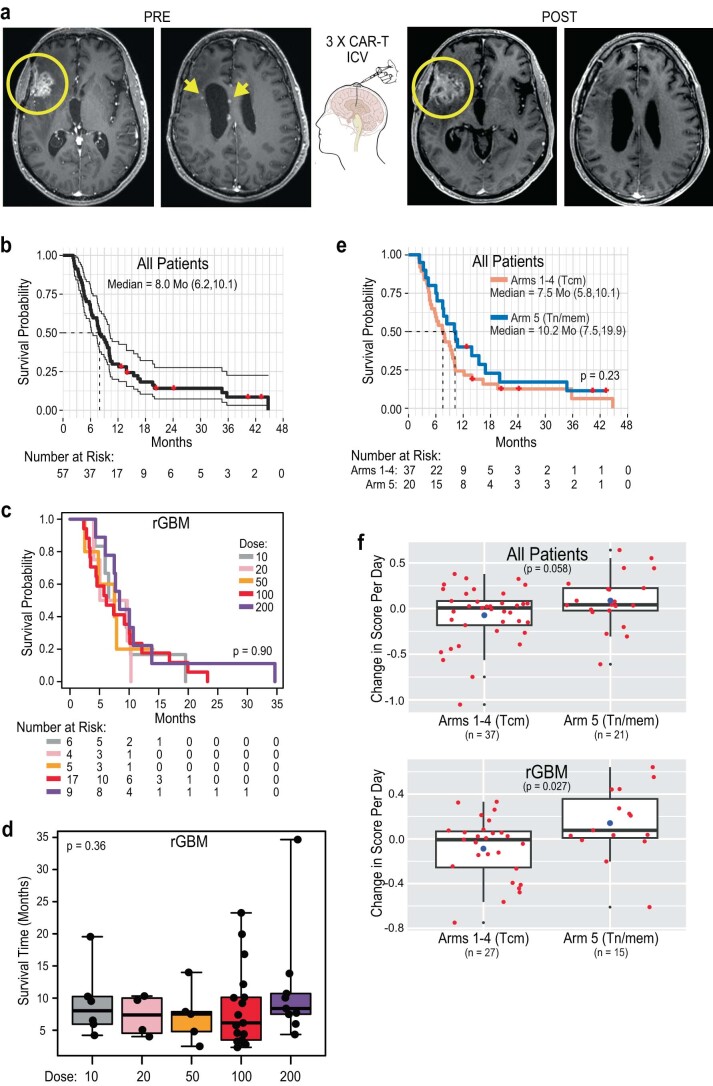
Effect of ICV delivered CAR-T cells for UPN145 and patient overall survival and quality-of-life evaluations. **a**, MR images of UPN145 under Arm 3 before (PRE, left panels) and after (POST, right panels) three ICV infusions of CAR-T cells at DS1 show that while the subpial lesions (yellow arrows) resolved, the parenchymal tumor (yellow circle) progressed. **b**, Overall survival of all evaluable patients from date of surgery. Thin lines denote 95% CIs; dashed lines depict medians in months (Mo). Median survival times with 95% CIs in parentheses are indicated. **c, d**, Survival comparisons of rGBM patients by dose schedule utilized for the DLT period. Legend of (c) and x-axis of (d) indicate maximum infusion cycle dose x 10^6^. Box and whisker plots of (d) depict the median and interquartile range with whiskers extending to the minimum and maximum values. P-values were determined using the two-sided Log-rank test (c) or an ANOVA test (d) across the different dose levels (n = 6, 4, 5, 17, and 9, left to right). **e**, Survival comparison of all evaluable patients that were infused with either Tcm- or Tn/mem-derived cell products. Red dots indicate censored participants (that is, lost to follow-up but hadn’t passed away). P-values for survival comparisons were determined using the Log-rank test. **f**, EROTC QLQ-C30 summary score slopes (change in QOL scores per day) from pre-surgery to ~ day 38 (encompassing the three infusions and DLT period) for all evaluable participants (top) or evaluable participants with rGBM (bottom) are shown as box and whisker plots. The median and interquartile range with whiskers extending to the last point within 1.5 times the interquartile range are depicted for Arms 1-4 vs. Arm 5. The means are indicated as blue filled circles. Note the pre-cycle 1 score was used as baseline for four participants who did not have pre-surgery scores, and for 3 participants, 1 question for the timepoint was imputed with cycle 1 results. P-values were calculated using the two-sided *t*-test.

The trial opened as a two-arm study treating patients ICT following either biopsy (arm 1) or resection (arm 2). Arms 3–5 were added by protocol amendments based on clinical observations (Methods). ICV delivery (arm 3) was added on the basis of clinical experience with UPN109, in which ICV administered IL-13Rα2-CAR-T cells mediated a CR against multifocal rGBM^6^, along with preclinical data suggesting ICV was more effective against multifocal tumors^16,20^. Subsequently, the trial transitioned to dual delivery combining attributes of both ICV and ICT (arms 4 and 5)^16,20,21^, and after noting that ICV delivery eliminated small, multifocal subpial lesions, whereas large intraparenchymal tumor progressed (Extended Data Fig. 1a). The protocol was amended to include the Tn/mem manufacturing platform (arm 5) based on data suggesting superior activity against hematological malignancies for Tn/mem- versus Tcm-derived CAR-T cell products^22–27^, along with feasibility challenges with generating sufficient Tcm-derived CAR products for the highest dose schedule (DS3) in arm 4 (refs. ^22–27^). Arm 5, (Tn/mem manufacturing and dual ICT/ICV delivery), therefore, represents the foundation for ongoing and future clinical testing (NCT04003649, NCT04510051 and NCT04661384).

**Fig. 2:**
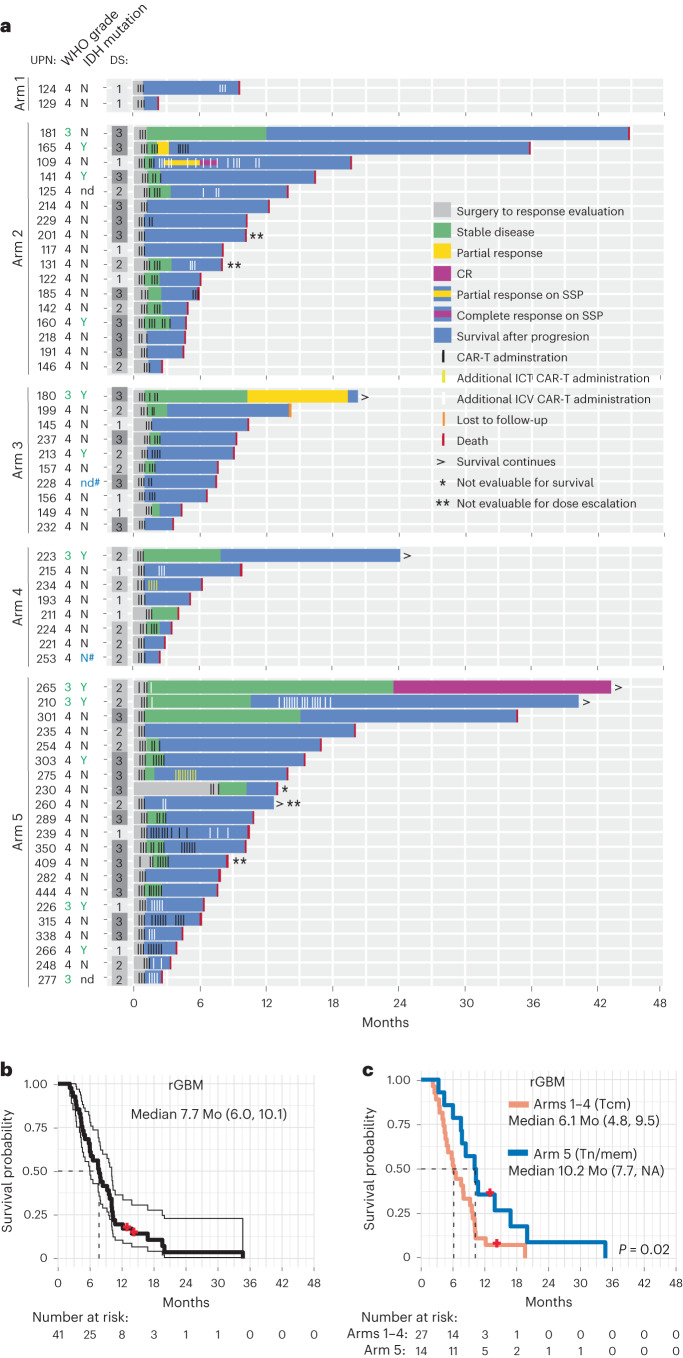
Clinical activity of locoregionally delivered IL-13Rα2-CAR-T cells. **a**, Swimmer plot of evaluable patients and their clinical outcomes. WHO grade is at time of treatment. Tumor IDH mutations are indicated by yes (Y) or no (N); ND, not determined; DS, dose schedule. Black lines indicate CAR-T cell cycles administered to route dictated by arm (arm 1: ICT-Biopsy; arm 2: ICT-Resection; arm 3: ICV; arms 4 and 5: dual ICT/ICV). White lines indicate additional CAR-T cell cycles administered ICV. Yellow lines indicate additional CAR-T cell cycles administered ICT. Bold UPN numbers, evaluable rGBM patients (*n* = 42, evaluable for survival *n* = 41); #, diffuse midline glioma, H3 K27-altered. **b**, OS of evaluable rGBM patients from date of surgery. Thin lines denote 95% CIs; dashed line depicts median in months (Mo). Median survival with 95% CI in parentheses also indicated. **c**, Survival comparison of evaluable rGBM patients who were infused with either Tcm- or Tn/mem-derived cell products. Dashed lines depict medians in Mo. Median survival times with 95% CIs in parentheses also indicated; NA means infinity. *P* value for survival comparison was determined using the log-rank test. Red dots indicate censored participants (that is, lost to follow-up but had not passed away).

A total of 92 patients were enrolled between June 2015 and February 2020. Patients underwent apheresis for CAR manufacturing, and CAR-T products were successfully produced from 97% (89/92) (Fig. 1c). Due to rapid tumor progression, 24 patients did not receive their manufactured CAR-T product. Sixty-five patients received at least one CAR-T infusion (Extended Data Table 1). Fifty-eight patients received at least three CAR-T infusions and were evaluable for disease response (*n* = 58), overall survival (OS; *n* = 57) or dose escalation (*n* = 54) (Figs. 1c and 2a, and Supplementary Table 1). UPN230 was not evaluable for survival due to the extended wait between surgery and CAR-T treatment; and four participants (UPN201, UPN131, UPN260 and UPN409) were not evaluable for dose escalation due to either receipt of <80% of the CAR-T cell dose or disallowed therapy (‘Study design’ section in Methods).

### Toxicity

Primary objectives of this trial were to evaluate feasibility and safety, and to determine a maximum tolerated dose (MTD) and/or maximum feasible dose (MFD) schedule for informing a recommended phase 2 dose (RP2D) plan of locoregionally delivered IL-13Rα2-CAR-T cells. Primary endpoints monitored grade 3 toxicities, DLTs and all other toxicities. No DLTs were noted for any treatment arm or dose schedule. The most common toxicities with possible or higher attribution to CAR-T cells were fatigue, headache and hypertension. Grade 3 and above toxicities with possible or higher attribution to CAR-T cells were seen in 35% (95% confidence interval (CI) 24–48%) of patients (Tcm arms 1–4: 17/43, 40%, 95% CI 25–56%; Tn/mem arm 5: 6/22, 27%, 95% CI 11–50%), with one grade 3 encephalopathy and one grade 3 ataxia with probable attribution to CAR-T cells. Although serious adverse events associated with brain inflammation (such as cerebral edema; an exploratory endpoint) was not a common toxicity associated with CAR-T cell administration, two patients with extensive residual/recurrent tumors did experience transient grade 4 cerebral edema shortly after receiving cycle 1 with possible attribution to CAR-T cells (Table 2). Patients’ symptoms improved within a few days of increasing dexamethasone (16 mg per day UPN243 and 36 mg per day UPN288); however, steroid levels could not be reduced to the protocol limit of 6 mg per day due to continued tumor progression, and therefore additional CAR-T cell infusions were not allowed. Toxicities with possible or higher attribution to the Rickham catheter or surgery are reported in Extended Data Table 2. Twenty-two of the 58 participants received additional CAR-T cells off protocol therapy (that is, after progressing or after going on disallowed treatment). Overall, all routes of delivery (ICT, ICV and dual ICT/ICV) and infusion dose levels (2–200 × 10^6^ CAR + T cells) were generally well tolerated, with toxicities clinically managed by increasing systemic steroids (up to 6 mg of dexamethasone per day) and palliative care (hydration, antipyretics, analgesics and antiemetics). Together these findings support a clinical MFD of 200 × 10^6^ CAR-T cells per infusion cycle as achieved for arm 5; however, since toxicity alone may not be the best benchmark for cellular immunotherapies, additional studies are needed to refine the RP2D plan on the basis of biological and/or response endpoints.

**Table 2:** Summary of toxicities attributed to CAR-T cells*

CTCAE v4.0 AE category	AE detail	Arms 1 and 2 (ICT) *N* = 20	Arm 3 (ICV) *N* = 11		Arm 4 (Dual) *N* = 12		Arm 5 (Dual Tn/mem) *N* = 22
		Grade 3^ No. (%)	Grade 3 No. (%)	Grade 4 No. (%)	Grade 3 No. (%)	Grade 4 No. (%)	Grade 3^ No. (%)
**Gastrointestinal disorders**	Nausea	0	1 (9)	0	2 (17)	0	0
	Vomiting	1 (5)	0	0	2 (17)	0	0
**General disorders and administration site conditions**	Fatigue	1 (5)	1 (9)	0	0	0	0
	Gait disturbance	0	0	0	1 (8)	0	0
**Investigations**	Alanine aminotransferase increased	0	0	0	0	0	1 (5)
	Lymphocyte count decreased	2 (10)	0	0	0	0	0
**Metabolism and nutrition disorders**	Hyponatremia	0	0	0	0	0	1 (5)
**Musculoskeletal and connective tissue disorders**	Back pain	0	0	0	0	0	1 (5)
	Neck pain	0	0	0	0	0	1 (5)
	Generalized muscle weakness	0	0	0	1 (8)	0	0
**Nervous system disorders**	Ataxia	0	0	0	0	0	1 (5)**
	Depressed level of consciousness	0	1 (9)	0	0	0	0
	Dysphasia	1 (5)	1 (9)	0	0	0	0
	Edema cerebral	0	0	1 (9)	0	1(8)	0
	Encephalopathy	0	0	0	1 (8)**	0	0
	Headache	3 (15)	1 (9)	0	3 (25)	0	2 (9)
	Hydrocephalus	0	1 (9)	0	0	0	1 (5)
	Nervous system disorders—left side Hemiparesis	0	0	0	1 (8)	0	0
	Peripheral motor neuropathy	0	1 (9)	0	0	0	0
**Vascular disorders**	Hypertension	2 (10)	4 (36)	0	1 (8)	0	2 (9)

^*^Events of grade 3 or higher, according to the NCI Common Toxicity Criteria, with possible or higher attribution to CAR-T cell administration while on protocol therapy are reported.

^^^No events of grade 4 or higher with possible or higher attribution to CAR-T cell administration while on protocol therapy were observed on arms 1, 2 or 5.

^**^Events (grade 3 or higher) that were probably attributed to CAR-T cell administration while on protocol therapy.

### Patient outcomes

Secondary objectives of this trial included the assessment of disease response rates, OS and changes in QOL for patients that received the full schedule of three CAR-T cell doses. Of the 58 patients evaluable for disease response, 29 (50%) achieved stable disease (SD) or better (range 51 to >1,316 days), and 13 (22%) achieved confirmed SD or better for ≥90 days, with 8 of the 13 having rHGG grade 4 (Fig. 2a and Supplementary Table 1). Two patients achieved a partial response (UPN165 and UPN180), and one patient achieved a CR (UPN265); of note, all three tumors were IDH-mutated and two were grade 3. An additional therapy-mediated CR for a rGBM (IDH wildtype) was achieved for UPN109 after ICV CAR-T cell administration on an SSP, and, importantly, this individual received no other therapy between and/or during CAR-T cell treatments^6,19^.

Median OS for all patients was 8 months, and 7.7 months for the subset with rGBM (Table 1, Fig. 2b and Extended Data Fig. 1b). Median OS was not strongly influenced by dose (Extended Data Fig. 1c,d), thereby providing rationale for combining dose schedules for survival comparisons. Arm 5 achieved the best OS of 10.2 months independent of disease stage (Fig. 2c and Extended Data Fig. 1e). Indeed, post hoc analysis of patients with rGBM revealed that those on arm 5 exhibited superior survival as compared to patients enrolled on arms 1–4 (arm 5: 10.2 months; arms 1–4: 6.1 months; *P* = 0.02) (Fig. 2c). Although this was a nonrandomized study, inclusion criteria remained consistent over trial execution, and participants on arms 1–4 versus arm 5 had similar baseline characteristics for age, sex, KPS, IDH mutation status, O^6^-methylguanine DNA methyltransferase (MGMT) methylation status, H score, multifocal disease, tumor resections and steroid administration at registration (Table 1 and Extended Data Table 3). Noted differences between groups included arm 5 having lower median tumor volumes pre-CAR-T treatment; however, correcting for this covariate the survival benefit remains significant (*P* = 0.024). Arm 5 also had fewer participants with prior bevacizumab treatment based on increased concerns with wound healing. We could not statistically adjust for this difference since there were too few participants in arm 5 with prior bevacizumab^28^; however, we note that UPN301, the only rGBM participant treated with prior bevacizumab on arm 5, had the longest survival.

As a secondary endpoint, changes in QOL were also scored throughout treatment on the basis of a 0–100 scale using the QLQ-C30 summary. Post hoc analysis of QOL scores from presurgery through the DLT period revealed a modest, but significant, increase in slopes for arm 5 over arms 1–4 for rGBM participants (*P* = 0.027) (Extended Data Fig. 1f), consistent with the longer OS for arm 5 (Fig. 2c).

Overall, IL-13Rα2-CAR-T cells mediated SD or better in 50% of heavily pretreated patients with rHGG, with arm 5 patients showing better OS as compared to arms 1–4.

### Superiority of Tn/mem-derived CAR-T cell products

**Extended Data Fig. 2:**
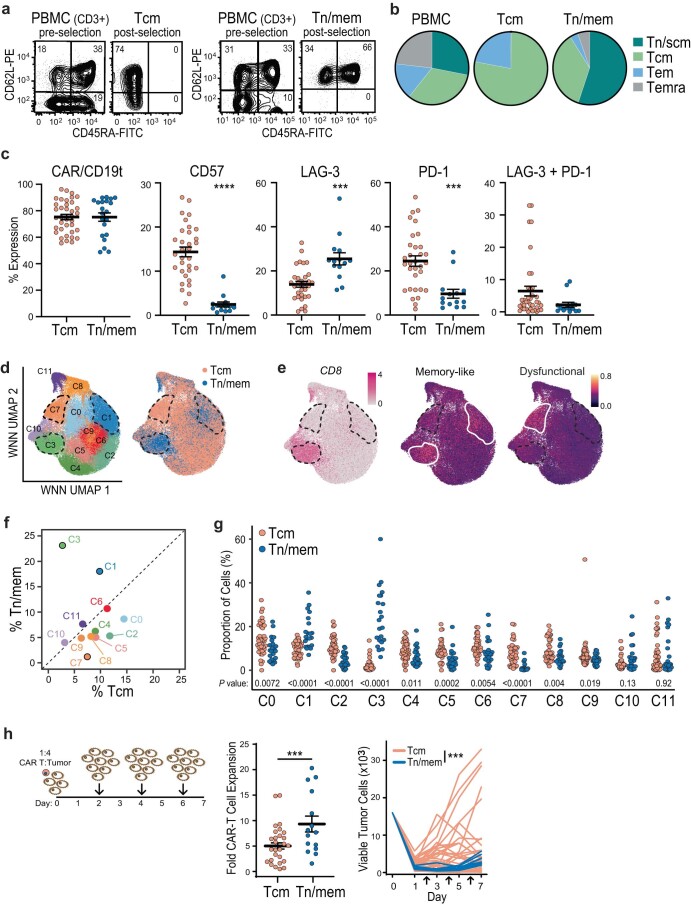
Characterization of Tcm- and Tn/mem-derived CAR-T cell products. **a**, Representative flow cytometry of CD62L/CD45RA before and after selection of either Tcm (left) or Tn/mem (right). **b**, Average distribution of T cell subpopulations in starting PBMC, or Tcm- or Tn/mem-selected cells based on CD62L/CD45RA analysis shown in (a). Tn/scm, CD62L + CD45RA + ; Tcm, CD62L + CD45RA-; Tem, CD62L- CD45RA-; Temra, CD62L- CD45RA + . **c**, Expression of CAR (using CD19t marker), CD57, LAG-3, PD-1 and co-expression of LAG-3 and PD-1 on Tcm- and Tn/mem-derived final CAR + T cell products as determined by flow cytometry, with means ( ± S.E.) indicated by black bars. ****, P < 0.0001; ***, P ≤ 0.0003 using a two-sided unpaired *t*-test. Due to limited cell product availability, only 46 products (n = 32 Tcm, 14 Tn/mem) were assayed for CD57, LAG-3 and PD-1. **d**, UMAP projection and clustering of 80,374 cells across 40 Tcm-derived and 22 Tn/mem-derived CAR-T products, colored by 12 T cell clusters (left), or by product type (right). Clusters 1, 3, and 7 are outlined. **e**, Log-normalized, scaled expression of *CD8* (left), and enrichment of gene signatures for memory-like and dysfunctional T cell subsets. **f**, Mean percentages of cells in each of the 12 clusters identified by scRNA sequencing (C0-C11, reference **d**) in Tcm-derived and Tn/mem-derived CAR-T cell product samples. **g**, Percentage proportions of Tcm- vs. Tn/mem-derived CAR-T cell products in each of the 12 clusters (C0-C11). Two-sided Wilcox-test p-values when comparing Tcm- and Tn/mem-derived products in each cluster are indicated. **h**, Schematic of *in vitro* tumor rechallenge assay (left). Tcm- or Tn/mem-derived CAR-T product expansion (mean±S.E.; middle) and tumor count (right). ***, P ≤ 0.0031 using either a two-sided unpaired *t*-test (left) or a two-sided *t*-test for the log area under the curve (right). Note, due to limited cell product availability, only 49 products (n = 34 Tcm, 15 Tn/mem) were used in the expansion assay, and 45 products (n = 32 Tcm, 13 Tn/mem) were used in the killing assay.

Patient specific factors, such as age, prior treatments and GBM-induced T cell dysfunction^29^, likely contribute to both reduced functional potency and increased variability in CAR-T product characteristics. We therefore immunomagnetically enriched less-differentiated memory T cells for CAR engineering with the goal of improving consistency and potency of autologous products. We initially implemented our Tcm (CD62L+, CD45RA−) GMP manufacturing platform (arms 1–4)^25,30^, and then transitioned to our clinically validated Tn/mem (CD62L+) platform (arm 5)^25^. Selection of patient peripheral blood mononuclear cells (PBMCs) for either Tcm or Tn/mem increased the homogeneity and early memory phenotype of the T cell population used for gene engineering. Tcm selection enriched for T cells with central memory (CD62L+, CD45RA−) and effector memory (CD62L−, CD45RA−) phenotypes, and Tn/mem selection enriched for T cells with naive, stem cell and central memory phenotypes (CD45RA+/− CD62L+) (Extended Data Fig. 2a,b). While both manufacturing platforms were successful in generating clinical products (95% feasibility for Tcm (56 of 59 products) and 100% feasibility for Tn/mem (33 of 33)) (Fig. 1c), the Tn/mem platform consistently yielded greater numbers of T cells available for CAR engineering, and therefore the total number of CAR-T cells manufactured per patient.

**Fig. 3:**
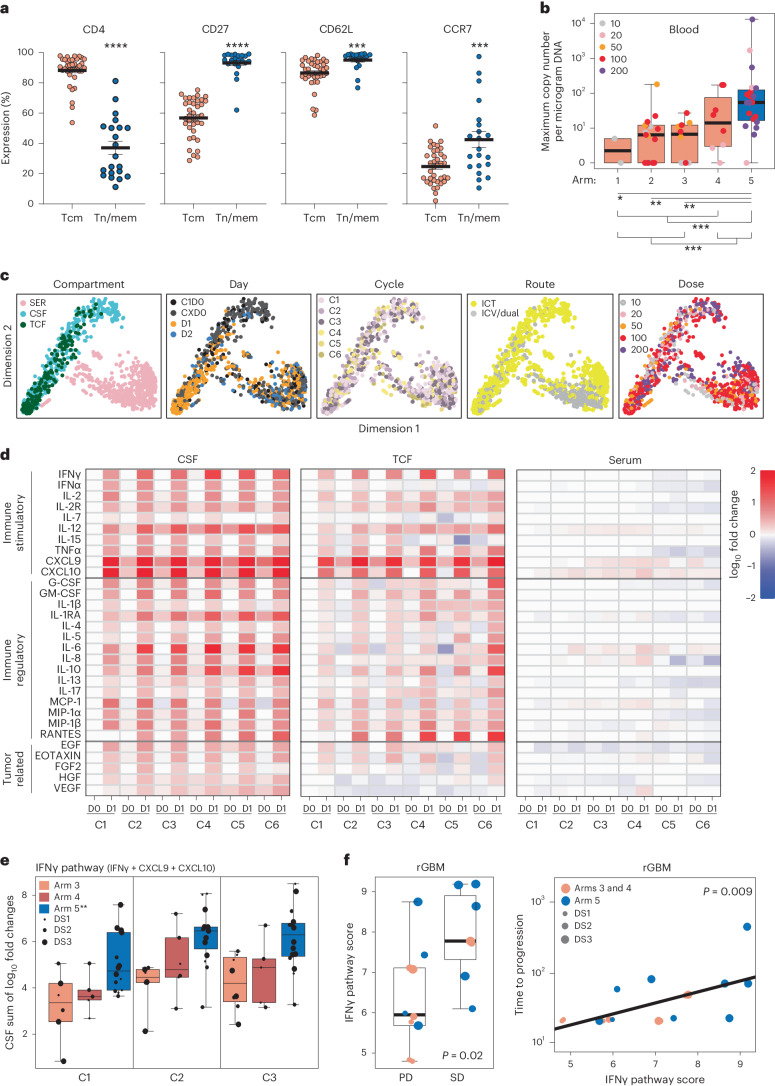
Correlative assessment of CAR-T persistence and patient cytokine profiles. **a**, Expression of CD4, CD27, CD62L and CCR7 on Tcm- and Tn/mem-derived CAR + T cell products (*n* = 58 cell products) as determined by flow cytometry, with means (± standard error of the mean) indicated by black bars. ****P < 0.0001; ***P ≤ 0.0005 using a two-sided unpaired *t*-test. **b**, Box-and-whisker plot of maximum WPRE copy number per microgram of PBMC DNA by arm (*n* = 2, 17, 10, 8 and 20, respectively, for arms 1–5; reference Extended Data Fig. 4e). The median and interquartile range with whiskers extending to the minimum and maximum values are depicted. *P = 0.075, **P ≤ 0.003 and ***P ≤ 0.0003 when using a two-sided *t*-test. Legend indicates maximum infusion dose per dose schedule (×10^6^). **c**, Low-dimensional representations of cytokine measurements colored by compartment (SER, CSF or TCF), day of any given cycle (up to six cycles, with CXD0 being day 0 of cycles 2–6), cycle number, delivery route or dose (legend indicates maximum infusion dose × 10^6^). **d**, Heatmap of cytokine levels across cycles (C1–6), preinfusion (D0) and postinfusion (D1) in either the CSF (left), TCF (middle) or serum (right), with the median log_10_ fold change from baseline (C1D0) shown. **e**, Box-and-whisker plot of the change in IFNγ pathway score (log_10_(IFNγ) + log_10_(CXCL9) + log_10_(CXCL10)) from baseline (C1D0) to the corresponding CXD1 within the CSF for survival evaluable patients treated on either arm 3, 4 or 5, for the first three cycles (at C1, *n* = 6, 5 or 14, respectively; at C2, *n* = 6, 6 or 15, respectively; at C3, *n* = 8, 5 or 16, respectively). The median and interquartile range with whiskers extending to the minimum and maximum values are depicted. Dose schedule (DS) is indicated by dot size; **P ≤ 0.0006 compared to each of the other arms using ANOVA. **f**, CSF IFNγ pathway score at C1D1 (log_10_(IFNγ) + log_10_(CXCL9) + log_10_(CXCL10)) in patients with rGBM (*n* = 15) evaluated relative to response. Left: box-and-whisker plot of CSF IFNγ pathway scores for PD versus SD or better following CAR-T treatment. The median and interquartile range with whiskers extending to the minimum and maximum values are depicted. Right: CSF IFNγ pathway scores plotted against time to progression in days, with the regression line for all arms depicted. Dose schedule (DS) indicated by dot size and *P* values determined by two-sided *t*-test.

We next phenotypically and functionally compared the final Tcm- and Tn/mem-derived CAR-T products, which was originally a secondary endpoint of our study and in the final protocol was redefined as an exploratory endpoint. While both platforms showed comparable levels of CAR-positive T cells, Tn/mem-derived CAR-T cells exhibited a more balanced proportion of CD4+ and CD8+ subsets, higher proportions of T cells expressing memory markers CD27, CD62L and CCR7, and lower expression of the senescence marker CD57 (Fig. 3a and Extended Data Fig. 2c). This more favorable phenotype for the Tn/mem platform was consistent with findings from healthy donor products (Supplementary Fig. 3a).

**Extended Data Fig. 3:**
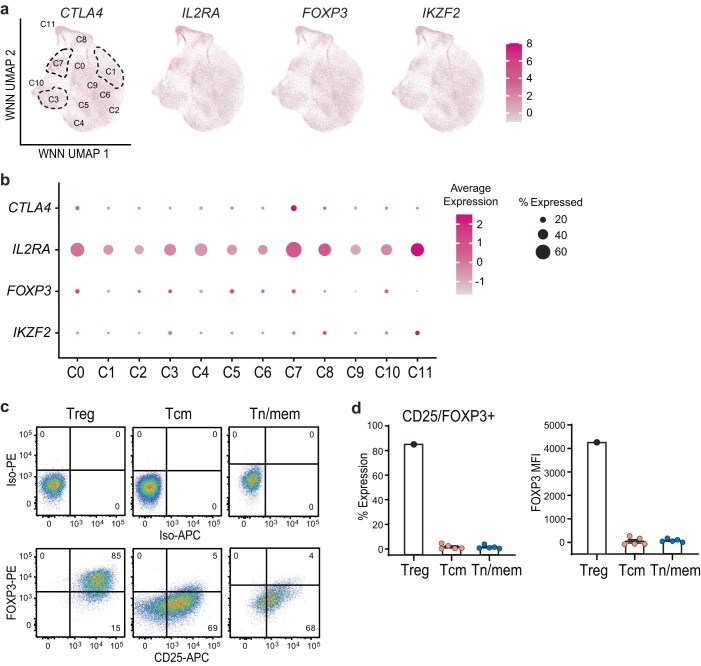
Evaluation of Tcm- and Tn/mem-derived CAR-T cell products for Treg cells. **a**, UMAP projection and log-normalized, scaled expression of *CTLA4* (with 12 clusters indicated as in Fig. 2b), *IL2RA*, *FOXP3* and *IKZF2* genes. **b**, Average expression, and the percentages of cells expressing *CTLA4*, *IL2RA*, *FOXP3* or *IKZF2* genes in each of the 12 clusters identified by scRNA sequencing (C0-C11). **c**, Representative flow cytometry of CD25/FOXP3 on viable, CD3+/CD4+/CAR(CD19t)+ -gated cells of either Tcm- or Tn/mem-derived CAR-T cell products as compared to a Treg CAR-T cell line control. Isotype control staining is depicted at top to confirm quadrant placement for each analysis. **d**, Mean (±S.E.) percentages of CD25/FOXP3+ cells (left) or mean fluorescence intensities (MFI) of FOXP3 expression (right) on viable, CD3+/CD4+/CAR(CD19t)+ -gated cells of n = 5 representative Tcm- or Tn/mem-derived CAR-T cell products as compared to a Treg CAR-T cell line control.

Gene expression profiles from 62 patient products (40 Tcm derived and 22 Tn/mem derived) by single-cell RNA sequencing (scRNA-seq) also reveal differences between Tcm- and Tn/mem-derived CAR-T products. We identified 12 clusters, and found that five clusters (C1, C2, C3, C5 and C7) showed the greatest differences in abundance between Tcm and Tn/mem products. In agreement with flow cytometry, Tn/mem-derived CAR-T products had higher expression of genes associated with a memory phenotype (CD8+ cells, C3; CD4+ cells, C1), and Tcm-derived CAR-T products showed enrichment of CD4+ cells expressing genes associated with T cell dysfunction (C7) (Extended Data Fig. 2d–g and Supplementary Tables 3 and 4). In contrast to other reports^31,32^, both Tcm and Tn/mem CAR-T products did not exhibit a distinct CD4+ regulatory T cell (Treg) population by either scRNA-seq or flow cytometry (Extended Data Fig. 3), possibly due to the CD25-depletion step in our manufacturing process.

Functional comparisons using a recursive tumor challenge assay revealed that Tn/mem-derived products exhibited superior proliferation and more potent target killing at high tumor:effector ratios as compared to Tcm-derived products (Extended Data Fig. 2h). Further, healthy donor Tn/mem-derived CD19-CAR and IL-13Rα2-CAR-T cell products exhibited improved anti-tumor activity in mice, as compared to Tcm-derived products (Supplementary Fig. 3b,c). Together, these data suggest that selection for Tn/mem cells yields autologous CAR-T products with more favorable and homogeneous phenotype and function for patients with glioma.

### Persistence and peripheral trafficking of CAR-T cells

**Extended Data Fig. 4:**
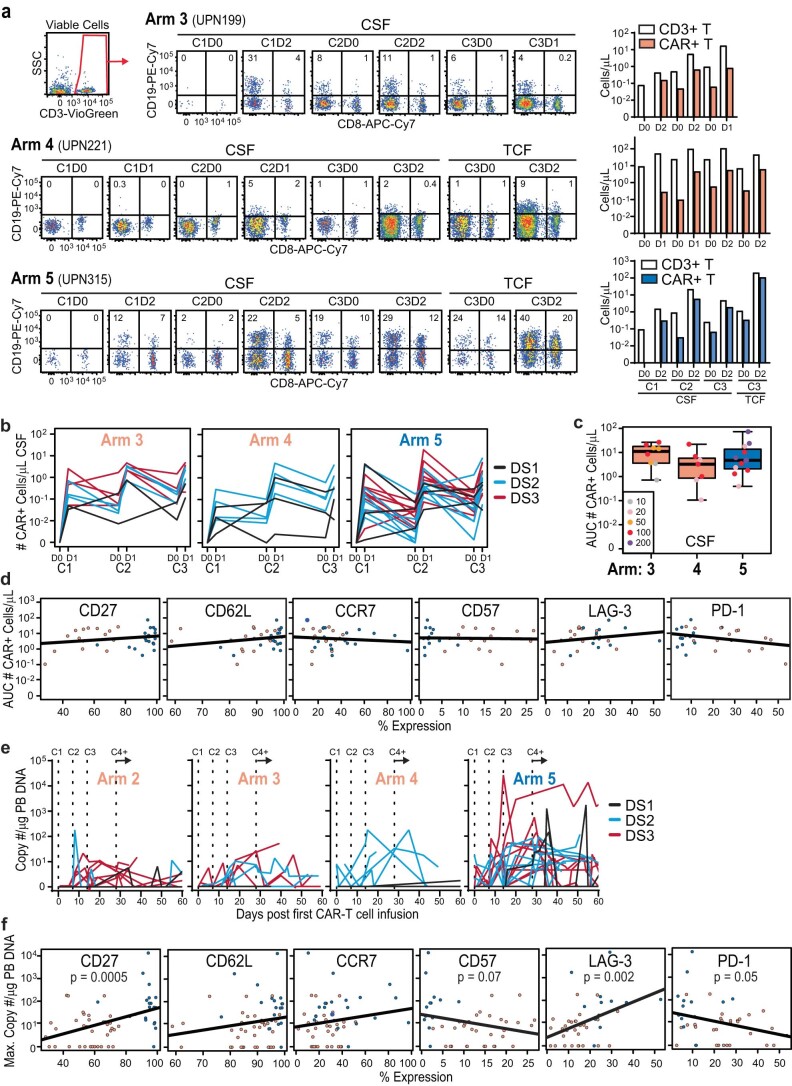
Detection of CAR-T cells in the CNS and blood. **a**, Representative flow cytometric analyses (left) and quantitation of viable CD3+ and CAR + T cells per µl (right), in CSF and TCF samples over the first three cycles (C1-C3) in Arm 3 (top), Arm 4 (middle) and Arm 5 (bottom). **b**, Graph summarizing numbers of CAR + T cells as determined by flow cytometry in the CSF for Arms 3-5 over the first three treatment cycles (C1-C3). D0, day 0 of that cycle prior to CAR-T cell infusion; D1, sample taken one or two days after CAR-T cell administration; DS, dose schedule. **c**, Box and whisker plot of AUC for number of CAR-T cells per µL in the CSF from cycles 1, 2 and 3 by arm (reference **a, b**; n = 9 for Arm 3, n = 7 for Arm 4, n = 19 for Arm 5). The median and interquartile range with whiskers extending to the minimum and maximum values are depicted. All comparisons between arms using *t*-test were nonsignificant. **d**, Percentages of the indicated surface marker expression of each CAR-T cell product were plotted relative to levels of detected CAR-T cells in the CSF (AUC measurements over cycles 1-3, reference **b,c**). Tcm- and Tn/mem-derived CAR-T cell products are depicted as peach and blue dots, respectively. Regression lines for all products are also depicted in each plot. **e**, WPRE copy numbers per µg of PBMC DNA are depicted over time. Cycles of CAR-T cell administration are indicated by dashed lines; note that for C4 + , day 28 to day 60, not everyone received 4 or more cycles of CAR-T cells, reference Fig. 2a. **f**, Percentages of the indicated surface marker expression of each CAR-T cell product were plotted relative to levels of detected CAR-T cells in the blood (maximum copy numbers of WPRE per µg of DNA observed over the first six cycles, reference **e** and Fig. 3b). Tcm- and Tn/mem-derived CAR-T cell products are depicted as peach and blue dots, respectively. Regression lines for all products, with significant p-values for each regression (via *t*-test), are also depicted in each plot.

As a secondary objective of this trial, IL-13Rα2-CAR-T cell persistence in cerebrospinal fluid (CSF), tumor cavity fluid (TCF) and blood was monitored throughout treatment (Extended Data Fig. 4). The availability of TCF and CSF varied with route of administration (TCF: arms 1, 2, 4 and 5; and CSF: arms 3–5) and clinical feasibility of liquid biopsy (Supplementary Fig. 5). Samples were collected at each cycle, before (day 0, D0) and 1 or 2 days after CAR-T administration (D1, D2), with cycle 1 (C1) D0 representing the pretreatment baseline and D0 of subsequent cycles representing 7+ days after the prior infusion. CAR-T cells were detected in the CSF and TCF for the majority of patients 1 day post infusion for at least one cycle, and, for a subset of patients, ≥7 days post infusion (Extended Data Fig. 4a,b), which is noteworthy since CSF volume turns over approximately four times per day^33^. CAR-T levels in the CSF modestly increased with higher dose schedules (Extended Data Fig. 4b and Supplementary Fig. 6a); however, there was no significant correlation with treatment arm (Extended Data Fig. 4c), or product marker expression (for example, CD27, CCR7, LAG-3 or PD-1) (Extended Data Fig. 4d).

Studies in mice suggest that CAR-T cells delivered into the CSF can traffic to the periphery^34^; however, in humans whether CAR-T cells delivered ICT or ICV can traffic to the periphery has not been established. Here we detected CAR-T cells in the blood, which was positively associated with dual delivery and highest in arm 5 (Fig. 3b and Extended Data Fig. 4e). CAR-T levels in the blood showed positive association with product CD27 and LAG-3 expression, and negative association with exhaustion markers PD-1 and CD57 (Extended Data Fig. 4f), but did now show a significant relationship with increased dose schedule (Supplementary Fig. 6b). These findings demonstrate that IL-13Rα2-CAR-T cells can persist in the CSF and TCF, and when delivered to the central nervous system (CNS) can traffic to the periphery, an observation that could have clinical implications.

### Locoregional spikes in inflammatory cytokines

**Extended Data Fig. 5:**
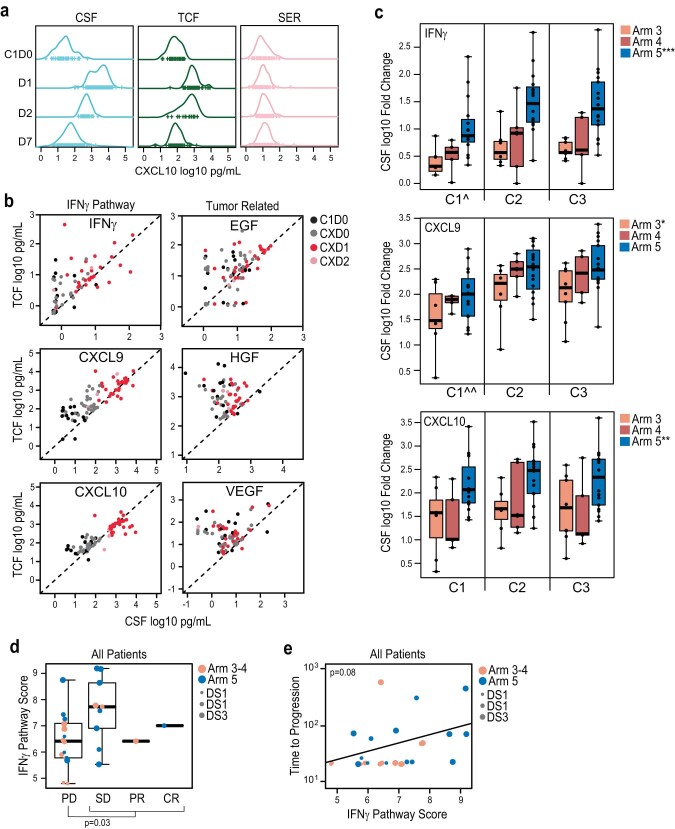
Cytokine profiles after IL-13Rα2-CAR-T cell therapy. **a**, CXCL10 levels depicted as density plots prior to CAR-T cell infusion (C1D0) and one, two- or seven-days post-infusion (D1, D2, D7) of cycles 1-6 in either the CSF (left), TCF (middle) or Serum (right). **b**, Plots of TCF (y-axis) versus CSF (x-axis) levels (log_10_) of IFNγ pathway cytokines (IFNγ, CXCL9 and CXCL10) and tumor-related cytokines (EGF, HGF, VEGF). Legend shows baseline (C1D0; black), subsequent pre-infusion (CXD0; grey), day 1 post-infusion (CXD1, red) and day 2 post infusion (CXD2, pink). Only measurements when a patient had concurrent CSF and TCF observations (reference Supplementary Fig. 5) are shown. **c**, Box and whisker plots of the fold changes from patients’ baseline to the corresponding CxD1 in each of the indicated individual IFNγ pathway cytokines within the CSF for patients treated on either arm 3, 4 or 5, for each of the first three cycles (at C1, n = 6, 5, or 14, respectively; at C2, n = 6, 6, or 15, respectively; at C3, n = 8, 5, or 16, respectively). The median and interquartile range with whiskers extending to the minimum and maximum values are depicted. By ANOVA: ***, p ≤ 0.00002; **, p ≤ 0.0003; and *, p ≤ 0.03 compared to each of the other arms in that graph; and ^^, p ≤ 0.0002; and ^, p ≤ 0.02 compared to each of the other cycles in that graph. **d**, Box and whisker plots of CSF IFNγ pathway score at C1D1 (log_10_(IFNγ) + log_10_(CXCL9) + log_10_(CXCL10)) in all evaluable, appropriately sampled patients who exhibited either PD (n = 15), SD (n = 9), PR (n = 1) or CR (n = 1) upon CAR-T cell treatment. The median and interquartile range with whiskers extending to the minimum and maximum values are depicted. P-value determined by two-sided *t*-test. **e**, CSF IFNγ pathway score in all evaluable patients was plotted against time to progression in days, and the regression line for all arms, with p-value (via two-sided *t*-test), is depicted. Arm 3 and 4 vs. Arm 5 patients are depicted as peach vs. blue dots, respectively, with dose schedule (DS) indicated by dot size (**d, e**).

We previously reported that IL-13Rα2-CAR-T administration induced spikes of inflammatory cytokines in the CSF of a patient with rGBM^6^. As a secondary objective of this trial, treatment-related cytokine dynamics in this larger cohort were explored in available CSF, TCF and serum samples (Supplementary Fig. 5). Low-dimensional visual representation of the 30 measured cytokines revealed global differences by compartment—CSF and TCF being distinct from serum, but together comprising a continuous band, with CSF clustering toward one end and TCF the other (Fig. 3c). When separated by day, a strong time dependence post-treatment is observed for CSF and TCF, with D1 measurements clustered closer to the TCF end and D0 closer to the CSF end (Fig. 3c). By day 7 after treatment, CSF and TCF cytokine levels decrease toward baseline (Extended Data Fig. 5a). Serum cytokine levels did not show an obvious time dependence. Cytokine dynamics were not strongly impacted by cycle number, suggesting limited additive or sensitizing effects with successive treatment cycles (Fig. 3c). While delivery route and dose levels (Fig. 3c) were also not a contributing factor to cytokine dynamics in the CSF and TCF, serum cytokines differentially clustered on the basis of delivery route (Fig. 3c), which is consistent with the detection of dual ICT/ICV delivered CAR-T cells in the periphery (Fig. 3b).

A qualitative overview of cytokine-specific fold change dynamics revealed infusion-dependent increases (CXD1) for almost every measured cytokine in the CSF and TCF (Fig. 3d). Of potential interest, IFNγ-pathway and Th1 immune-stimulatory cytokines, including IFNγ, CXCL9, CXCL10, IL-2R and IL-12 were strongly induced following treatment, and maintained appreciable levels 7+ days post-infusion (that is, day 0 of the next cycle). Modest decreases in tumor-related cytokines HGF and VEGF were observed in the TCF over treatment cycles (Fig. 3d, middle). Comparing changes in absolute cytokine levels for IFNγ and the IFNγ-inducible cytokines CXCL9 and CXCL10 to the tumor-related cytokines EGF, HGF and VEGF highlights the strong treatment related effects for IFNγ-pathway related cytokines (Extended Data Fig. 5b). In serum, by comparison, few treatment-dependent changes were observed, except for modest increases in immune stimulatory/regulatory cytokines IL-12, CXCL9, CXCL10 and IL-6, and modest decreases in IL-8, IL-13, MIP-1α, TNFα, IFNα, IL-2R, EGF and HGF (Fig. 3d).

Given that IFNγ-signaling pathway cytokines (IFNγ, CXCL9 and CXCL10) in both CSF and TCF showed some of the highest treatment-related increases, we explored whether this signature may be associated with treatment outcomes. We focused on CSF cytokine levels, as CSF volume is relatively similar between patients, and more consistently collected as compared to TCF (Supplementary Fig. 5). We note that, over the first three cycles of CAR-T administration, arm 5 patients showed a significantly higher CSF IFNγ signature as compared to those treated under arms 3 and 4 (Fig. 3e and Extended Data Fig. 5c), consistent with the improved median OS for this cohort (Fig. 2c). When comparing IFNγ-signature elevations to overall best response and time to progression, there is a positive correlation with patient outcomes, even for patients with the worst prognosis (that is, rGBM) (Fig. 3f and Extended Data Fig. 5d,e). Together, these findings suggest that the CSF IFNγ signature may be a useful biomarker for CAR-T cell activity.

### Intratumoral T cell levels correlate with clinical outcomes

**Fig. 4:**
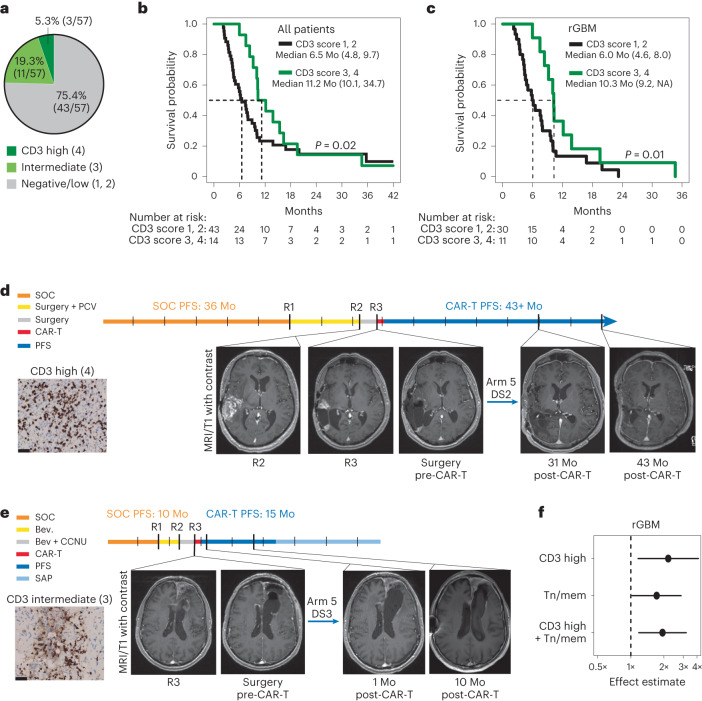
Pretreatment tumor T cell infiltrates and responses to CAR-T therapy. **a**, Distribution of tumors with high, intermediate or negative/low CD3 infiltrates in pretreatment tumors from patients evaluable for survival (*n* = 57). **b**,**c**, Survival comparison of all evaluable rHGG (**b**) or rGBM (**c**) patients with either negative/low (1, 2) or intermediate/high (3, 4) tumor CD3 IHC scores. Dashed lines depict medians in months (Mo). Median survival times with 95% CIs in parentheses are also indicated; NA means infinity. *P* values comparing survival distribution of each group using the two-sided peto–peto test are depicted. **d**,**e**, MRI images from CD3 high UPN265 (**d**) and CD3 intermediate UPN301 (**e**). Ticks in timelines indicate 6-month intervals; SOC, standard of care involving surgery, radiation and temozolomide; PCV, procarabaxine, CCNU and vincristine; Bev., bevacizumab; CCNU, lomustine; SAP, survival after progression. **f**, Linear regression model of log survival time for survival evaluable rGBM patients (*n* = 41) showing estimates of the survival effect for CD3 intermediate/high (3, 4), Tn/mem product (arm 5) or both. Parameters were compared to a CD3 low/negative (1, 2) and Tcm (arms 1–4) patient reference group. Point estimates of the effect of each variable, or both, are depicted as a multiplicative factor (center dot, with 95% CIs as horizonal lines) that is applied to survival time; 95% CI horizontal lines do not cross vertical dashed line, demonstrating *P* < 0.05 for each effect; reference Methods for statistical analysis details.

Secondary objectives of this trial aimed to identify tumor and tumor microenvironment (TME) biomarkers associated with response to CAR-T cell therapy. Given the role of the IFNγ pathway in CAR-T cell antitumor activity^13,35,36^, pretreatment tumors were evaluated for T cell infiltration, and scored for negative/low or intermediate/high CD3 levels (Fig. 4a and Supplementary Fig. 7). The majority of tumors had low CD3 infiltrates (43/57; scores of 1 or 2), consistent with the well-documented ‘cold’ TME of GBM^37^. By contrast, 25% of tumors had intermediate/high CD3 infiltration (14/57; scores of 3 or 4). CD3 levels also generally correlated with CD8 infiltration, and CD8 T cells were more abundant than FOXP3+ Tregs (Supplementary Fig. 8).

Evaluating pretreatment intratumoral T cell levels with survival following CAR-T therapy revealed that intermediate/high CD3 scores were associated with improved OS for all patients (*P* = 0.02) and the rGBM subset (*P* = 0.01) (Fig. 4b,c). Prognostic factors for patient survival (that is, age, gender, KPS, IDH or MGMT status) were similar between CD3-scoring cohorts (Extended Data Tables 4 and 5). There was a greater proportion of grade 4 tumors for the CD3 high cohort (13 of 14) when considering all patients (although not statistically significant), and despite this, CD3 levels remained associated with better OS. Of note, two of the three patients with the highest CD3 score of 4 (UPN109 and UPN265; Supplementary Fig. 7a) achieved a CR following IL-13Rα2-CAR-T therapy, and, for both of these patients, progression-free survival (PFS) was greater than that following SOC at initial diagnosis (Fig. 4d)^6^. The patient with rGBM with the longest survival post-CAR-T therapy (UPN301) had intermediate CD3 levels (score of 3), and despite treatment at third recurrence post-progression on bevacizumab, PFS following CAR-T therapy was again longer than following SOC (Fig. 4e).

Our results suggest that both product fitness and tumor contexture (CD3 score) are important parameters impacting CAR-T therapy. However, the proportions of intermediate/high CD3 tumors were not equally distributed between treatment arms. Arm 5 had more rGBM tumors with intermediate/high CD3 scores (7 of 14) versus arms 1–4 (4 of 27) (Extended Data Table 5). Conversely, arms 1–4 had more rGBM tumors with the highest CD3 score of 4 (2 of 4) versus arm 5 (0 of 7). To uncouple the effects of CD3 status and product (Tcm versus Tn/mem), we performed linear regression analyses with interaction term for CD3 score (1, 2 versus 3, 4) and product on log survival time (Fig. 4f). Both higher tumor CD3 levels and product composition (Tn/mem) positively correlated with better survival. The most significant survival benefit was observed with intermediate/high CD3 scores; these patients are estimated to survive twice as long in response to CAR-T therapy as compared to those with low CD3 scores. For ‘cold’ tumors, Tn/mem treatment was estimated to give a 1.6× benefit over Tcm treatment. However, no additive survival benefit was observed for CD3 intermediate/high patients receiving Tn/mem products (Supplementary Fig. 8e), indicating that the response of ‘hot’ tumors to CAR-T therapy is independent of product type.

## Discussion

To our knowledge, we report the largest CAR-T clinical trial in brain tumors, assessing the feasibility, safety and bioactivity of IL-13Rα2-CAR-T cells in rGBM and other HGGs. Three routes of locoregional delivery were evaluated (ICT, ICV and dual ICT/ICV) along with two manufacturing platforms (Tcm versus Tn/mem). Key findings include the following: (1) repetitive locoregional administration of IL-13Rα2-CAR-T cells is feasible and safe with no DLTs; (2) clinical benefit was observed in a subset of patients, including improved QOL, extended SD and transient radiographic response; (3) elevations in inflammatory and immune modulatory cytokines in the CSF and TCF occurred after each infusion, with the IFNγ pathway as a potential biomarker of CAR activity; (4) patients treated on arm 5 (dual ICT/ICV; Tnmem) displayed the greatest IFNγ-pathway induction in the CSF, and statistically significant improvement in OS as compared to other treatment arms; and (5) high pretreatment tumor T cell levels correlated with a significant survival benefit.

CAR-T products with a less-differentiated memory phenotype are positively associated with clinical response in hematological malignancies^26,38,39^. Products used in this clinical trial were manufactured from less-differentiated memory T cell subsets (Tcm and Tn/mem) with the intent of reducing levels of dysfunctional T cells reported in GBM^29^, improving potency and decreasing interpatient product heterogeneity. The Tn/mem platform improved manufacturing feasibility, increased product homogeneity, promoted a favorable less-differentiated phenotype (CD62L+, CCR7+ and CD27+), and displayed the highest CAR-T persistence in blood. Arm 5 utilizing Tn/mem-derived CAR-T products, exhibited the best OS compared to other treatment arms, consistent with previous reports of Tn/mem-derived outperforming Tcm-derived CAR-T products and mediating encouraging clinical benefit against hematological tumors^22,23,25,27^.

We chose to move forward with dual delivery in arms 4 and 5 to incorporate potentially unique attributes for each delivery route, with ICV delivery being advantageous for treating multifocal tumors, and ICT being beneficial for eradicating unifocal tumors^16,20,21^. However, mechanistic underpinnings for CAR-T trafficking based on delivery routes warrants further investigation. Locoregionally delivered CAR-T cells were detected in the peripheral blood, and CAR-T trafficking between the CSF and blood could be of clinical significance as CAR-T cells would be expected to maintain their effector activity^34^. Despite the detection of systemic CAR-T cells in the blood, we did not observe systemic toxicities. However, future studies will measure testosterone levels following IL-13Rα2-CAR-T therapy in males to specifically evaluate potential on-target, off-tumor activity, as IL-13Rα2 is a cancer-testis antigen^40^.

Analysis of cytokine dynamics revealed treatment related upregulation in proinflammatory/immune stimulatory cytokines in the CSF, as reported for other brain tumor studies evaluating CAR-T therapy^7,11,12^. Of note, IFNγ-pathway-related cytokines/chemokines (IFNγ, CXCL9 and CXCL10) were elevated in the CNS (CSF and TCF), with the highest levels in arm 5 patients. When evaluating clinical outcomes, the IFNγ pathway positively correlated with time to progression and best response. IFNγ-inducible chemokines CXCL9 and CXCL10 are ligands for CXCR3, a receptor expressed by diverse immune populations and reported to play important roles in immune cell recruitment and tumor immunity^41^. Overall, our findings demonstrate that liquid biopsy of the CNS was instructive for understanding treatment-related effects and suggest that CAR-T-induced IFNγ, CXCL9 and CXCL10 could be a clinically meaningful biomarker for patient response.

While the prognostic and predictive roles of the TME have been described for some solid tumors in the context of immunotherapy^42,43^, only recently has the importance of pretreatment TME for CAR-T therapy been reported for hematological malignancies^35^. The relationship between tumor contexture and responsiveness to CAR-T therapy has not been reported in more immunosuppressive solid tumors, such as GBM. Previously we reported that a patient with rGBM who achieved a CR to IL-13Rα2-CAR-T therapy despite heterogenous IL-13Rα2 expression had a T-cell-rich TME and, following CAR-T treatment, showed evidence for activation of host immunity^6,13^. Leveraging tumor samples from 57 evaluable patients on this trial, we demonstrate that pretreatment tumor CD3+ T cell levels positively correlated with enhanced survival following CAR-T therapy. While some studies have reported that T cell infiltrates positively correlate with survival of patients with GBM^44–46^, the participants treated on this trial had recurred through prior therapies and were actively progressing at the time of CAR-T treatment. Despite this, tumors with high/intermediate T cell infiltrates not only showed improved outcomes to CAR-T therapy, but in some instances, response (that is, PFS) was longer than initial response to SOC at diagnosis. Taken together, our findings suggest that a ‘hot’ TME plays a critical role in response to CAR-T therapy^13^.

Limitations of our study include the multiple treatment arms and dose schedules for correlative comparisons and the relatively small number of tumors with intermediate/high CD3 infiltrates. Our statistical analyses, however, aimed to account for these limitations. For instance, our logistic modeling allowed us to uncouple product fitness (Tn/mem) and tumor contexture (CD3), two parameters identified in our trial to positively impact CAR-T therapy. Our modeling also confirmed that tumor ‘hotness’ defined by CD3 density is a dominant feature impacting patient survival following CAR-T therapy. Of interest, clinical benefit was independent of manufacturing process, with Tn/mem-derived CAR products showing no benefit over Tcm. By comparison, our modeling suggests that CAR product was an important determinant for OS in ‘cold’ tumors, with Tn/mem outperforming Tcm. Future randomized studies in larger patient cohorts are needed to confirm and build on our findings related to the critical parameters for successful CAR-T therapy.

In summary, primary objectives of this phase I clinical study were met, establishing feasibility and safety of locoregionally delivered IL-13Rα2-CAR-T cells for treatment of rHGG and rGBM. Although we do not intend to proceed with arms 1–4 for future phase trials, these initial arms provided critical insights to the application of CAR-T cells for the treatment of malignant brain tumors, as no other study has compared the feasibility and safety of various CNS delivery routes. Instead, our intention is to move forward with arm 5, which achieved a MFD of 200 × 10^6^ CAR-T cells per infusion and displayed the best OS of 10.2 months. Our post hoc analysis found that arm 5 survived longer than treatment arms 1–4, which provided a reasonable benchmark control group since enrollment criteria remained consistent over trial execution and participants on arms 1–4 had similar baseline patient characteristics as arm 5. However, due to limitations for post hoc analyses and because this was a nonrandomized trial a survival benefit cannot be inferred. While it will be important to confirm our findings in later-phase trials, OS for the heavily pretreated rGBM cohort, the majority of which were treated at second recurrence or later, compared favorably with historical controls. Median OS for GBM at first recurrence ranges from 5.5 to 12.6 months^47^ and post bevacizumab from 3.3 to 3.4 months^48^. The phase III trial evaluating tumor treating fields versus chemotherapy, which, as with our study, treated the majority of patients at second recurrence or later and allowed prior bevacizumab, reported a median OS of 6.6 and 6.0 months, respectively^49^. Current efforts are now aimed at next-generation CAR designs and combination therapies to address known barriers to more effective therapy, and thus enhance response rates and durability of the arm 5 platform.

## Methods

### Study design

Patients with rGBM and other HGGs enrolled on this phase I study between June 2015 and February 2020 (ClinicalTrials.gov Identifier: NCT02208362). This study (final protocol included in Supplementary Information) was conducted in accordance with the Institutional Review Board, Data Safety Monitoring Committee and Independent Ethics Committee at The City of Hope (COH) National Medical Center as well as the US Food and Drug Administration. All subjects provided written informed consent in accordance with local regulatory review. Patients were not compensated for their participation in the study.

Enrollment criteria included the following: age 18–75 years, which was modified to 12–75 years by protocol amendment; diagnosis of progressive/recurrent grade 3 or 4 malignant glioma^18^; IL-13Rα2+ tumor expression by immunohistochemistry (IHC); KPS ≥60; life expectancy >4 weeks; appropriate venous access; defined washout periods from previous therapies; steroid dependency ≤6 mg dexamethasone a day; adequate organ function, including white blood cell count >2,000 dl^−1^ (or absolute neutrophil cell count >1,000 dl^−1^), platelets ≥100,000 µl^−1^ and international normalized ratio <1.3; creatinine <1.6 mg dl^−1^, bilirubin <1.5 mg dl^−1^, alanine transaminase and aspartate transaminase <2.5× upper limits of normal, oxygen saturation ≥95% and a lack of radiographic abnormalities on chest X-ray that are progressive, lack of symptomatic cardiac arrhythmias and does not require pressor support, lack of uncontrolled seizure activity; negative blood cultures for bacteria, fungus or virus and no indications of meningitis; and use of adequate contraception in patients of child-bearing age. Sex or gender was not considered in the study design as rHGG occurs in both males and females.

Exclusion criterial included the following: requires supplemental oxygen to keep saturation greater than 95%, and the situation is not expected to resolve within 2 weeks; symptomatic arrythmia requiring intervention; requires dialysis; uncontrolled seizure activity and/or clinically evident progressive encephalopathy; patient and/or legal guardian unable to understand basic elements and risks/benefits of participating on the study; poorly controlled or severe intercurrent illness or active infection including hepatitis B or C; another active malignancy; recovering from major surgery until recovery deemed complete by the investigator; and confirmed human immunodeficiency virus positivity within 4 weeks of screening.

This five-arm trial evolved to evaluate three routes of locoregional delivery (ICT, ICV and dual ICT/ICV) and two manufacturing platforms (Tcm and Tn/mem). In May 2015, the trial opened as a two-arm study treating patients ICT following either biopsy (arm 1) or resection (arm 2). The rationale for two arms was based on concerns that the higher tumor burden for nonresectable tumors may result in greater toxicities; however, after treating the first two participants on arm 1, we found that the therapy was well tolerated with no DLTs. We therefore amended the protocol to close enrollment on arm 1 and enrolled biopsy patients on other treatment arms. We have combined arm 1 and arm 2 for safety and survival analyses, since both were treated with ICT administration. Concomitant with closing arm 1, we amended the protocol to open arm 3 to evaluate ICV delivery of CAR-T cells. The rationale for arm 3 (ICV) was based on our clinical experience with the first participant treated on arm 2 who received ICV-delivered CAR-T cells on an SSP and achieved a complete remission of his multifocal rGBM^6^. We then enrolled participants with unifocal tumors on arm 2 and multifocal tumors on arm 3. Subsequently, we amended the protocol to open arm 4 to evaluate delivery of CAR-T cells both ICT and ICV (dual), as preclinical and clinical data suggested benefits to both delivery routes^6,16,20,21^. Enrollment between arms 2, 3 and 4 was based on slot availability due to patient staggering, disease presentation (unifocal tumors were primarily enrolled on arm 2), and efforts to establish a MFD for each delivery route. Lastly, the protocol was amended for arm 5 to evaluate our modified Tn/mem manufacturing process with dual ICT/ICV delivery based on preclinical and clinical experience (Supplementary Fig. 3 and refs. ^22–27^) and increased manufacturing feasibility. We prioritized for enrolling patients on arm 5 and began to close enrollment on all other treatment arms.

Upon enrollment, research subjects were assigned a UPN, and their PBMCs were collected at the COH Donor Apheresis Center for CAR-T cell manufacture. The median time from apheresis to first CAR-T cell infusion was 50 days (minimum 22 days, maximum 1,241 days). Following radiographic evidence of progression, research subjects then underwent a stereotactic biopsy or resection followed by placement of a Rickham catheter(s)—those in arms 1 and 2 had one catheter placed in or proximal to the magnetic resonance imaging (MRI)-defined tumor site (ICT); those in arm 3 had one catheter placed in the lateral cerebral ventricle (ICV); and those in arms 4 and 5 had two catheters placed both ICT and ICV. Following surgery and within 1–2 weeks before the first CAR-T cell infusion, research subjects underwent baseline MRI and fluorodeoxyglucose-positron emission tomography (FDG-PET) imaging. The median time from surgery and catheter placement to the first CAR-T cell infusion (C1) was 9 days (minimum 6 days, maximum 29 days). Patients ineligible for treatment as indicated in Fig. 1c included those who never went to surgery or those who went to surgery but did not receive CAR-T cells.

On the morning of each T cell infusion, the IL-13Rα2-CAR-T cells were thawed, washed and reformulated in 0.5 ml preservative-free normal saline/2% human serum albumin (HSA) and delivered manually using a syringe to inject into the Rickham catheter. This was followed by a 0.5 ml preservative-free normal saline flush over approximately 5 min through the Rickham catheter. Participants on dual ICT/ICV arms (arms 4 and 5) simultaneously received the intended dose into each ICT and ICV catheter. Note that CAR-T cells were administered without prior lymphodepleting chemotherapy.

Dose escalation within each arm followed a 3 + 3 design. Participants were deemed evaluable for dose escalation if they received 80% of each of their assigned three weekly infusions and were followed for one additional week or experienced a DLT and did not receive disallowed therapy or experienced a delay between doses of more than 21 days. Participants were evaluable for response or survival if they received three doses of CAR-T cells in the DLT period. UPN230 was not evaluable for survival due to the extended wait between surgery and the first CAR-T cell infusion. Four participants were not evaluable for dose escalation due to either receipt of <80% of the CAR-T cell dose at cycle 3 (UPN201) or cycle 2 (UPN131), receipt of disallowed therapy on day 24 (UPN260), or a longer than allowable delay between cycles 1 and 2 (UPN409).

Toxicities reported here are limited to those that occurred on protocol therapy. Toxicities were followed at least twice a week during the 28-day DLT period. After the DLT period participants were followed for worst grade toxicities monthly, then every 3 months for the first year and yearly thereafter. Adverse events were graded according to the CTCAE v4.0, as well as the revised cytokine release syndrome (CRS) grading system and the modified neurological grading system. DLTs were defined as serious adverse events probably or definitely attributed to CAR-T cell infusion, specifically any grade 3 allergic reaction or autoimmune reaction, grade 3 CRS lasting >72 h, any two grade 3 toxicities at the same dose lasting >72 h, and any grade 4 toxicities except those associated with CRS lasting <72 h. Full details on toxicity evaluation and DLTs are provided in the final protocol. Participants were imaged to assess disease response using modified response assessment in neuro-oncology (RANO) criteria at the end of the DLT period and every 2–3 months thereafter as clinically indicated.

Participants could continue receiving CAR-T cell infusions at a rate no more frequent than once a week and at less than or equal to the highest tolerated cell dose in the initial dose schedule, provided that the participant continued to meet eligibility criteria and there were cell doses available from the already manufactured cell product. If a research participant on arms 1 or 2 (ICT) progressed after the first three CAR-T cell infusion cycles, they were allowed to receive their optional CAR-T cell infusions ICV. For research participants on arms 4 or 5 (dual ICT/ICV), based on clinical response after the first three infusions, optional infusions could occur at either one or both sites (instead of requiring injections at both ICT and ICV sites) at less than or equal to the highest dose deemed safe for that delivery site.

### MRI and PET acquisition and analysis

MRI of the brain and spine were acquired on a Siemens MAGNETOM Verio 3.0 Tesla scanner. Pre, post, dynamic contrast enhanced, dynamic susceptibility contrast, gadolinium T1-weighted, diffusion and T2-weighted sequences were acquired. Tumor foci were measured on axial T1 multi-planar reconstruction (MPR)-weighted images obtained after the administration of MultiHance (gadobenate dimeglumine). Response assessment was recorded using the modified RANO criteria for GBM. Imaging with ^18^F-fluorodeoxyglucose was performed using a GE Discovery DST HP60 PET-CT scanner (70 cm axial field of view, slice thickness 3.75 mm). Maximal standardized uptake values were obtained utilizing Vital Images Vitrea version 6.7.2 software. For the calculation of contrast-enhancing tumor volumes, T2-weighted, T2-weighted Fluid Attenuated Inversion Recovery, T1-weighted pre- and post- contrast images were coregistered and resampled to 1 mm × 1 mm × 3 mm voxel sizes using BraTumIA software^50^. The registered, non-skull-stripped images were then imported into ITK-SNAP (v3.8.0) for segmentation^51^. Trained readers generated initial masks of the tumor volumes with final review and volume selection by a radiologist with over 10 years of experience in neuroradiology.

### QOL assessment

Research participant QOL assessment was evaluated using EROTC QLQ-C30 summary scores on a scale of 0–100 (the higher the score the better) with three to six scores collected from each patient over 38 days. The patient-reported multidimensional health related QOL questionnaire was composed of 13 items—five functional scales (physical, role, emotional, cognitive and social), three symptom scales (fatigue, nausea and vomiting, and pain) and five additional single items (dyspnea, insomnia, appetite loss, constipation and diarrhea).

### Clinical vector and IL-13Rα2-CAR-T cell manufacturing

The codon optimized CAR sequence contains a membrane-tethered human IL-13 ligand mutated at a single site (E13Y) to reduce potential binding to IL-13Rα1 (refs. ^52,53^), a human IgG4 Fc spacer containing two mutations (L235E; N297Q) that prevent Fc receptor-mediated recognition^54^, a human CD4 transmembrane domain, a human costimulatory 4-1BB cytoplasmic signaling domain, and a human CD3ζ cytoplasmic signaling domain. A T2A ribosome skip sequence^55^ then separates this IL-13Rα2-targeting CAR sequence from a truncated human CD19 sequence (CD19t), an inert, nonimmunogenic cell surface marker. Details for the generation of the lentiviral vector encoding the IL-13Rα2-CAR and the CD19t transgene are available upon request.

For IL-13Rα2-CAR-T cell manufacturing, on the day of leukapheresis, PBMCs were isolated by density gradient centrifugation over Ficoll-Paque (GE Healthcare) in either a centrifugation or Sepax cell separation system followed by two washes in phosphate-buffered saline (PBS)/ethylenediaminetetraacetic acid (EDTA). PBMCs were then washed once in PBS, resuspended in X Vivo15 medium (Bio Whittaker) containing 10% fetal calf serum (FCS; Hyclone), and stored on a 3-D rotator overnight at room temperature. The following day, PBMCs were incubated with clinical-grade anti-CD14 and anti-CD25 microbeads with (for Tcm) or without (for Tn/mem) anti-CD45RA microbeads (Miltenyi Biotec). CD14/CD25+ (for Tn/mem) or CD14/CD25/CD45RA+ (for Tcm) cells were then depleted using the CliniMACS depletion mode according to the manufacturer’s instructions (Miltenyi Biotec). After centrifugation, the unlabeled negative fraction of cells was resuspended in CliniMACS PBS/EDTA buffer (Miltenyi Biotec) containing 0.5% HSA (CSL Behring) and then labeled with clinical grade biotinylated-DREG56 mAb (COHNMC CBG). The cells were then washed and resuspended in CliniMACS PBS/EDTA containing 0.5% HSA and then incubation with anti-biotin microbeads (Miltenyi Biotec). The CD62L+ fraction was purified with positive selection on CliniMACS according to the manufacturer’s instructions, and resuspended in X Vivo15 containing 10% FCS.

Following enrichment, Tcm (CD14/CD25/CD45RA−, CD62L+) or Tn/mem (CD14/CD25−, CD62L+) were stimulated with GMP Dynabeads Human T expander CD3/CD28 (Invitrogen) at a 1:3 ratio (T cell:bead), and transduced with clinical-grade *IL-13BBζ-T2A-CD19t* lentivirus at an MOI of 0.3 in X Vivo15 containing 10% FCS with 5 μg ml^−1^ protamine sulfate (APP Pharmaceutical), 50 U ml^−1^ rhIL-2 and 0.5 ng ml^−1^ rhIL-15. Cultures were then maintained at 37 °C, 5% CO_2_ with addition of X Vivo15 10% FCS as required to keep cell density between 4 × 10^5^ and 2 × 10^6^ viable cells ml^−1^, with cytokine supplementation (final concentration of 50 U ml^−1^ rhIL-2 and 0.5 ng ml^−1^ rhIL-15) three times per week. CD3/CD28 Dynabeads were removed approximately 7 days after transduction using the Dynal ClinEx Vivo Magnetic Particle Concentrator bag magnet. Cultures were propagated until sufficient cell numbers were generated as determined by Guava PCA, at which time cultures were collected, washed in Isolyte (Braun) with 2% HSA, then resuspended in Cryostor CS5 (BioLife Solutions) for cryopreservation. Overall, this manufacturing process was completed in 10+ days (schema depicted in Supplementary Fig. 1). Quality control tests on freshly thawed cells included viability, potency (CD19t expression), identity (CD3 expression), transgene copy number (woodchuck hepatitis virus posttranscriptional regulatory element (WPRE) quantitative polymerase chain reaction (qPCR)), replication competent virus testing (vesicular stomatitis virus G protein (VSV-G) qPCR and formal replication competent lentivirus (RCL) testing at the University of Indiana), residual bead count and sterility. Cell products were further analyzed by flow cytometry as described below. Manufacturing failures as indicated in Fig. 1c consisted of two inadequate aphereses and one failed quality control release.

### Patient sample processing

Tumor resection material was collected through the COH Department of Pathology according to the clinical protocol. Peripheral blood samples were collected in vacutainer tubes ±EDTA. Samples with EDTA were ficolled immediately upon receipt, and PBMCs were frozen in Crystor CS5 at −80 °C, followed by transfer to liquid nitrogen for long-term storage. Samples without EDTA were allowed to coagulate for 2–3 h at room temperature; serum was collected by centrifugation, aliquoted in single-use 100–200 µl aliquots and stored at −80 °C. TCF was collected from the ICT reservoir, and CSF was collected from the ICV reservoir in a 3cc syringe, spun down, and cell-free supernatants were aliquoted and stored at −80 °C. The CSF cells were resuspended in HBSS−/− (Corning CellGro) with 2% FCS and sodium azide for immediate flow cytometric analysis as described below, with the remaining cells resuspended and frozen in Cryostor CS4 at −80 °C and then transferred to liquid nitrogen for long-term storage.

### Immunohistochemistry

IL-13Rα2 IHC was performed in the COH Clinical Pathology CLIA laboratory using 5-µm sections of formalin-fixed paraffin-embedded specimens. Slides were loaded on a Ventana DISCOVERY ULTRA IHC automated stainer (Ventana Medical Systems, Roche Diagnostics), where deparaffinization, rehydration, endogenous peroxidase activity inhibition and antigen retrieval (using TRIS buffer pH 8) were first performed. The slides were then incubated with a monoclonal rabbit anti-human IL-13Rα2 (E7U7B, Cell Signaling Technology, diluted 1:100) for 32 min followed by incubation with reagents from the OptiView DAB IHC Detection Kit. The stains were counterstained with hematoxylin and coverslipped. The stained slides were scanned using a NanoZoomer 2.0-HT digital slide scanner, a NanoZoomer S360 Digital Slide Scanner (Hamamatsu Corporation), or directly acquired on an Olympus BX46 transmitted light microscope with an SC-180 Olympus camera. IL-13Rα2 immunoreactivity was scored by a clinical neuropathologist and quantified based on the percentage of tumor cells exhibiting weak (1+), moderate (2+) or strong (3+) intensity of cytoplasmic and golgi-like staining. The H score is obtained by the formula: (3 × percentage of strongly staining cells) + (2 × percentage of moderately staining cells) + (percentage of weakly staining cells), giving a range of 0 to 300 (modified from ref. ^56^). The H score can be translated into the intensity scoring system described in the enrollment criteria as follows: 0 representing negative (H score 0), 1+ low (H score 1–100), 2+ moderate (H score 101–200) and 3+ high (H score 201–300). Appropriate positive (testicular) and negative (prostate) controls were employed for IL-13Rα2 IHC staining. While the criteria for patient inclusion on the trial was at least 20% of the cells scoring 1+ staining intensity (an H score of 20) at the time of enrollment, Supplementary Table 1 reports the highest H score from multiple blocks at time of surgery, with the ‘+’ sign reflecting the presence of membranous staining. This test was performed at the Department of Pathology, COH National Medical Center and is regarded as investigational for research. This laboratory is certified under the Clinical Laboratory Improvement Amendments of 1988 (CLIA) as qualified to perform high-complexity clinical laboratory testing.

CD3 IHC was performed similar to IL-13Rα2 IHC with the exceptions that slides were loaded on a Leica BOND autostainer, and the BOND Ready-To-Use Primary Antibody CD3 (LN10) was used according to manufacturer recommendations. Qualitative scoring for CD3 was performed by a clinical neuropathologist as follows: tumors with few detectable T cells were given a CD3 IHC score of 1; those with scattered CD3+ cells throughout, with possible occasional perivascular aggregates or hot spots, were given a score of 2; those with many CD3+ cells throughout and dense hot spots were given a score of 3; and those with many dense CD3+ cell infiltrates were given a score of 4.

Four-plex and 3-plex IHC was performed similar to IL-13Rα2 IHC with each antibody staining being carried out to completion using the Ventana DISCOVERY ULTRA IHC automated stainer, and with heat inactivation performed to prevent any cross-reactivity between each antigen detection. Stains were visualized with DISCOVERY Purple Kit, Yellow Kit, Teal Kit and Red kit (for 4-plex) or DISCOVERY DAB Kit, Purple Kit and Green Kit (for 3-plex) before counterstain with haematoxylin. Reference Supplementary Fig. 8 for chromogen colors, and Supplementary Reporting Summary for antibody information. Cell quantification was performed using Visiopharm v2023.01 software.

Images depicted in Fig. 4d,e and Supplementary Figs. 7a–c and 8a–c are representative of the whole tumor section/slide (that is, one per patient) that was stained by IHC and scored or quantified as described above.

### Flow cytometry

Cells were washed and immunophenotyped by flow cytometry using fluorochrome-conjugated antibodies specific for either CCR7, CD3, CD4, CD8, CD19, CD25, CD27, CD45RA, CD57, CD62L, FOXP3, LAG-3 or PD-1 (Supplementary Reporting Summary). Gating strategies are depicted in Supplementary Fig. 2. For flow-cytometry-based recursive killing assays, patient CAR-T cell products were cocultured with patient-derived glioma tumor cells, and absolute numbers of viable tumor cells and CAR-T cells were enumerated by staining for CD45 and CD19 to distinguish between target cells and CAR-T cells^57^. All samples were acquired on MACSQuant Analyzer 10 (Miltenyi Biotec) and analyzed with FlowJo software (v10.1, TreeStar) and GraphPad Prism Software (v9).

### Cytokine profiling

Serum, CSF and TCF samples were analyzed by the Analytical Pharmacology Core Facility at COH using the Human Cytokine 30-Plex Panel kit (Invitrogen) and a FLEXMAP 3D (Luminex).

### qPCR for CAR-T cell persistence

Assessment of CAR-T cell persistence in peripheral blood was determined by quantification of the WPRE region of the lentiviral transgene by qPCR. Genomic DNA (gDNA) was extracted from frozen 0.3-ml aliquots of whole blood and tested for the WPRE copy number by TaqMan qPCR. Average copy numbers are presented if ≥2 of 3 replicates generated a cycle threshold (Ct) value. Participants were measured for WPRE before and at least once after every CAR-T cell infusion.

### Orthotopic xenograft models

Raji-ffluc and patient-derived glioma line PBT030-2-ffluc-IL-13Rα2+ were maintained as previously described^16,58^. All tumor lines were authenticated for the desired antigen/marker expression by flow cytometry, tested for mycoplasma using the MycoAlert PLUS Mycoplasma Detection Kit (Lonza), and maintained in culture for less than 1–2 months. Tcm or Tn/mem products were enriched from healthy donors and transduced with lentiviral vectors encoding the CD19- or IL-13Rα2-targeting CAR as described above and previously described^16,59,60^. The resulting CAR-T cells were cultured for 18–21 days and analyzed by flow cytometry as described above.

All animal studies were approved by the COH Institutional Animal Care and Use Committee with mice housed in rooms with a 12 h:12 h light:dark cycle, ambient temperature of 68–75 °F, and 30–70% humidity. For the lymphoma studies, NOD/Scid IL2RγCnull (NSG) mice (9–10 weeks old) were injected with 0.5 × 10^6^ Raji-ffluc cells (CD19+) intravenously on day 0. Three days after tumor inoculation, mice were treated intravenously with Tcm- or Tn/mem-derived CD19-CAR-T cells or mock-transduced controls (1 × 10^6^). For GBM studies, NSG mice (6–10 weeks old) were injected with 0.1 × 10^6^ patient-derived glioma cell line (IL-13Rα2+) intracranially as described before^16^. Tcm- or Tn/mem-derived IL-13Rα2-CAR-T cells or mock-transduced controls (0.1 × 10^6^ cells) were administered intratumorally on day 8. OS was assessed using Kaplan–Meier methods (GraphPad Prism Software v9).

### scRNA-seq

#### RNA library preparation and single-cell sequencing

Single-cell sequencing of cryopreserved samples was carried out using the 10x Chromium platform. scRNA-seq was carried out on excess available CAR-T cell product samples from 62 of the 65 treated patients (40 Tcm, 22 Tn/mem). Additionally, expression levels of cell surface proteins of 27 CAR-T product samples (14 Tcm, 13 Tn/mem) were quantified using cellular indexing of transcriptomes and epitopes sequencing (CITEseq).

For batch 1, 51 patient PBMC exomes were collected via Illumina exome panel and sequenced at 20 M read pairs per patient for sample deconvolution. For batches 2–19, Biolegend Total Seq-C hashtag antibodies were used to allow sample deconvolution after pooled processing. Eight barcoded samples were sorted at equal proportions into a single collection tube, and the eight batches (batches 2–6, 14, 17 and 18) were treated with BioLegend Total Seq-C Human Universal Cocktail V1.0 for cell surface protein expression. Batch 1 was treated with TS-C #99328 with 198 antibodies, batches 2–6 with TS-C #99814 with 192 antibodies, and batches 14, 17 and 18 with TS-C #399905 with 137 antibodies (Supplementary Table 2). A total of 60,000 cells were loaded to a single Gel Bead-in-Emulsion (GEM) reaction onto the Chromium instrument. scRNA-seq and feature barcode libraries were prepared according to manufacturer protocols and sequenced on Illumina Iseq100 for cell count validation and NovaSeq6000 at the recommended depth for relevant library type. Sequence data were processed using 10x Genomics Cell Ranger V5.0 and Ensemble 98.

#### scRNA-seq bioinformatics

Single-cell sequencing data were analyzed using Seurat v4 (ref. ^61^). CellRanger objects for each batch were imported to create a Seurat object for each of the 19 batches. For batch 1, sample identities were deconvoluted and multiplets were identified using Demuxlet^62^. Exome sequencing FASTQ reads were chunked to 40 M reads, aligned to GRCh38 using the Burrows-Wheeler Aligner (BWA)^63^, and processed with Samtools Fixmates and Samtools Sort^64^. Individual chunks were merged, and PCR and optical duplicates were marked with Samtools. Genotypes were called with DeepVariant^65^. The single-cell data were filtered to retain singlets with >500 unique RNA features detected, >1,000 RNA feature counts and less than 10% of reads mapping to mitochondrial genes. Outliers with >10,000 protein feature counts were excluded.

Batches with both scRNA-seq and CITEseq gene expression data were normalized with the SCTransform function, protein data log-transformed, and each data type was integrated using reciprocal principal component analysis (rPCA). The number of principal components (PCs) to be included in dimensionality reduction and unsupervised clustering were determined by calculating the difference between the proportion of variation associated with each PC and their subsequent PC and selecting the last point where the difference is more than 0.1%. For each data type, Uniform Manifold Approximation and Projection (UMAP) was used for dimensionality reduction and 2D visualization of cell clusters. After clustering based on surface protein expression, data were filtered to exclude cells expressing high levels of control antibodies. Clustering based on both gene expression and surface protein expression levels was carried out using the weighted nearest neighbor analysis. For the batches with only scRNA-seq, gene expression data were similarly SCT normalized and rPCA integrated. After dimensionality reduction with principal component analysis (PCA), cluster labels and protein data were transferred from the reference object (RNA-seq + CITEseq) to the query (only RNA-seq) by projecting the query data onto the UMAP structure of the reference. The two objects were merged using the weighted nearest neighbor UMAP. The SCT assay was replaced with data integrated across all 62 samples. Three clusters with low numbers of cells (353, 257 and 2) were excluded from downstream analyses.

Highly expressed marker features for each cluster were identified using the presto implementation of the Wilcoxon rank test^66^. Clusters were annotated on the basis of cluster markers and canonical T cell marker expression (Supplementary Table 3). To further annotate the clusters on the basis of the expression of previously defined T cell expression signatures^67–69^ (Supplementary Table 4) we conducted a single-cell gene-set variation analysis (scGSVA) using the scGSVA R package^70^.

### Statistical analysis

The sample sizes for each arm were based on estimating the DLT rate and was done for 6 and 12 participants. A sample size of 6 at the MTD/MFD (1) provided a maximum margin of error of 0.35 for a 90% CI for the DLT rate, and (2) allowed us to detect a toxicity with a true rate of 0.25 in 82% of trials. A sample size of 12 (1) provided a maximum margin of error of 0.25 for a 90% CI, and (2) allowed us to detect a toxicity with a true rate of 0.25 in 96% of trials.

Survival calculations and estimates were generally performed using Kaplan–Meier methods, with post hoc tests of survival curve differences being from the Harrington–Fleming G\rho family as indicated in figure legends. To adjust for potential differences in tumors size between arm 5 and arms 1–4 for survival, a weighted log rank test with stabilized weights^71^ was used. The weights were based on propensity scores calculated using a logistic regression model predicting treatment assignment based on log_2_ pre-CAR-T cell tumor volume. The estimate of effects of CD3 score and Tn/mem product on expected survival times for patients with rGBM (Fig. 4f), was performed using a linear regression model of log survival time with covariates of CD3 score (high versus low), product (Tcm versus Tn/mem) and interaction. Within the rGBM group, there is only one censored observation, which was accounted for by imputation, noting that in general log-survival times among the rGBM group are well modeled through a normal distribution. Robustness of the imputation results was checked by observing that this single censored observation would need to be an extreme outlier before it would affect the significance of the 95% CIs obtained.

QLQ-C30 was used to assess QOL, and descriptive statistics were estimated for baseline score and slopes were determined over a 38-day period that included the three CAR-T infusions in the DLT period. Post hoc comparisons were made between the Tcm arms 1–4 and Tn/mem arm 5 for QOL using a two-sided two-sample *t*-test. No corrections were made for multiple testing.

Area under the curve of CAR-T cells in CSF was calculated using linear interpolation between time points, with the linear *x*-axis scale in units of days.

Low-dimensional representation of the 30-dimensional cytokine data was obtained by the application of multidimensional scaling to the proximity matrix generated by a random forest trained on the data.

Density plots were constructed using kernel density estimators with gaussian kernels and bandwidth estimated through cross-validation.

Statistical significance tests between groups with a single factor were generally performed using two-sided *t*-tests after appropriate checks on normality assumptions. Where the variable of interest failed this test and normality assumptions (that is, H scores), a nonparametric Mann–Whitney test was used. No corrections were made for multiple testing. Significance tests between mutifactorial groups (cytokine changes in the CSF over both cycles and arms) were performed with analysis of variance (ANOVA) to obtain significance levels for the factor of interest (arm).

Shown linear regressions were performed with standard least squares methods and significance of associations found by standard *t*-test.

We defined the absolute IFNγ pathway score of available C1D1 CSF samples as the sum of the log-transformed measured values of IFNγ, CXCL9 and CXCL10. Figure 3e shows the fold change in this score from baseline (C1D0).

### Reporting summary

Further information on research design is available in the Nature Portfolio Reporting Summary linked to this article.

## Online content

Any methods, additional references, Nature Portfolio reporting summaries, source data, extended data, supplementary information, acknowledgements, peer review information; details of author contributions and competing interests; and statements of data and code availability are available at 10.1038/s41591-024-02875-1.

### Supplementary information

Supplementary Information Supplementary Figs. 1–8 and final protocol.

Reporting Summary

Supplementary Tables 1–4 Supplementary Table 1. Response-evaluable patient characteristics. Supplementary Table 2. CITEseq antibodies. Supplementary Table 3. Differentially expressed genes that define scRNA-seq clusters. Supplementary Table 4. Gene signatures.

## Figures and Tables

**Extended Data Table 1:**
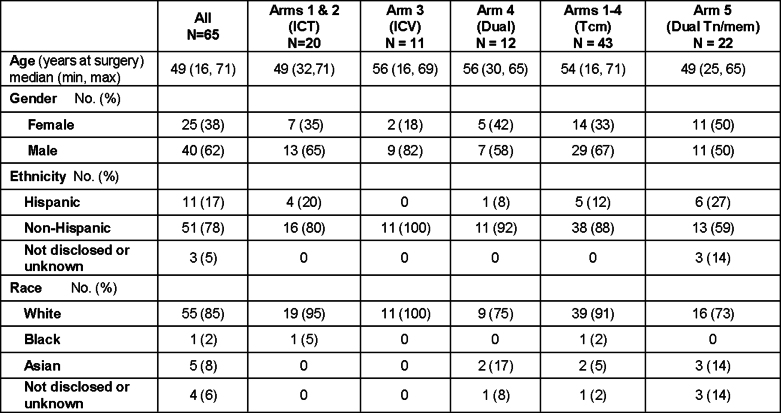
Patient Demographics

**Extended Data Table 2:**
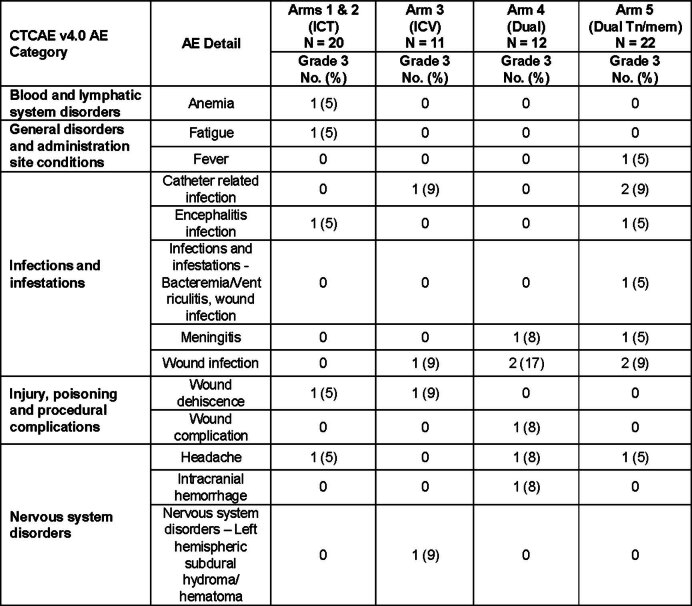
Grade 3 or Higher Toxicities with Possible or Higher Attribution to Surgery and/or Rickham Catheter while on Protocol Therapy

**Extended Data Table 3:**
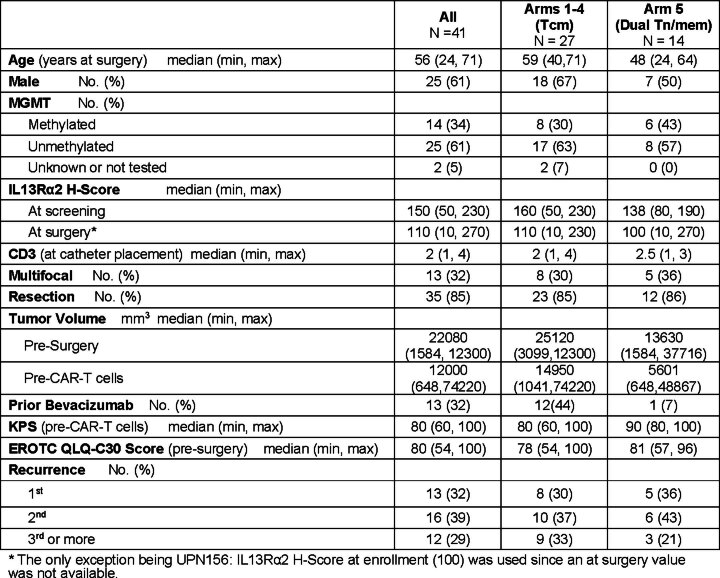
Characteristics of rGBM Patients that were Evaluable for Survival

**Extended Data Table 4:**
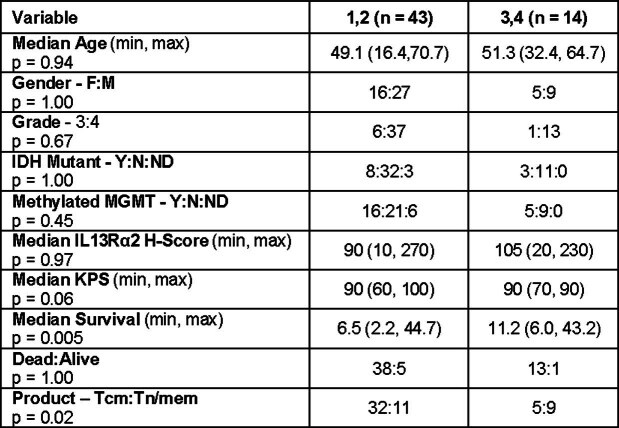
Breakdown of Variables by CD3 Level

**Extended Data Table 5:**
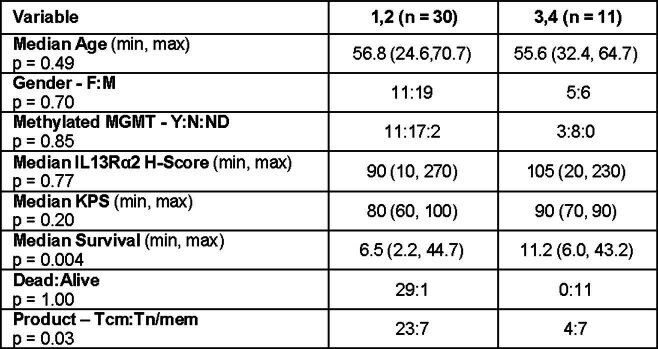
Breakdown of Variables in rGBM Patients by CD3 Level

## Data Availability

All requests for raw and analyzed data and materials will be promptly reviewed by the corresponding author and appropriate COH committees to verify if the request is subject to any intellectual property or confidentiality obligations. Requests may be made to cbrown@coh.org; response time will be within approximately 30 business days. Release of individual-level data may be restricted due to patient confidentiality considerations. Any data and materials that can be shared will be de-identified and released via a data or material transfer agreement. The RNA-sequencing data are deposited on the National Center of Biotechnology Information Gene Expression Omnibus (NCBI GEO) under the accession number GSE255850.

## Code availability

Scripts to conduct the RNAseq analyses are publicly available on GitHub at https://github.com/Banovich-Lab/13384_IL-13Ra2_CART_product ref. ^72^.
