# Author Correction: Locoregional delivery of IL-13Rα2-targeting CAR-T cells in recurrent high-grade glioma: a phase 1 trial

**DOI:** 10.1038/s41591-024-02928-5

**Published:** 2024-03-21

**Authors:** Christine E. Brown, Jonathan C. Hibbard, Darya Alizadeh, M. Suzette Blanchard, Heini M. Natri, Dongrui Wang, Julie R. Ostberg, Brenda Aguilar, Jamie R. Wagner, Jinny A. Paul, Renate Starr, Robyn A. Wong, Wuyang Chen, Noah Shulkin, Maryam Aftabizadeh, Aleksandr Filippov, Ammar Chaudhry, Julie A. Ressler, Julie Kilpatrick, Paige Myers-McNamara, Mike Chen, Leo D. Wang, Russell C. Rockne, Joseph Georges, Jana Portnow, Michael E. Barish, Massimo D’Apuzzo, Nicholas E. Banovich, Stephen J. Forman, Behnam Badie

**Affiliations:** 1 Department of Hematology & Hematopoietic Cell Transplantation (T Cell Therapeutics Research Laboratories), City of Hope Beckman Research Institute and Medical Center, Duarte, CA USA; 2 Department of Computational and Quantitative Medicine, City of Hope Beckman Research Institute and Medical Center, Duarte, CA USA; 3 The Translational Genomics Research Institute, Phoenix, AZ USA; 4 Department of Neurosurgery, City of Hope Beckman Research Institute and Medical Center, Duarte, CA USA; 5 Department of Diagnostic Radiology, City of Hope Beckman Research Institute and Medical Center, Duarte, CA USA; 6 Department of Clinical Research, City of Hope Beckman Research Institute and Medical Center, Duarte, CA USA; 7 Departments of Immuno-Oncology and Pediatrics, City of Hope Beckman Research Institute and Medical Center, Duarte, CA USA; 8 Department of Medical Oncology, City of Hope Beckman Research Institute and Medical Center, Duarte, CA USA; 9 Department of Stem Cell Biology & Regenerative Medicine, City of Hope Beckman Research Institute and Medical Center, Duarte, CA USA; 10 Department of Pathology, City of Hope Beckman Research Institute and Medical Center, Duarte, CA USA; 11 Present Address: Bone Marrow Transplantation Center, the First Affiliated Hospital, and Liangzhu Laboratory, Zhejiang University School of Medicine, Hangzhou, China

**Keywords:** Cancer immunotherapy, CNS cancer, Immunotherapy

Correction to: *Nature Medicine* 10.1038/s41591-024-02875-1, published online 7 March 2024.

In the version of the article initially published, two California Institute for Regenerative Medicine grant numbers, TRAN1-TR3-05641 and CLIN2-10248 (to C.E.B.), were inadvertently omitted from the Acknowledgements section and have now been added to the HTML and PDF versions of the article.

