# Supplementary material for: Locoregional delivery of IL-13Rα2-targeting CAR-T cells in recurrent high-grade glioma: a phase 1 trial
Source: Nat Med. 2024 Mar 7;30(4):1001–12. doi: 10.1038/s41591-024-02875-1 (PMC11031404; doi:10.1038/s41591-024-02875-1)
Supplement: Supplementary file 1 — Supplementary Figs. 1–8 and final protocol. [file 41591_2024_2875_MOESM1_ESM.pdf]

nature medicine

Article https://doi.org/10.1038/s41591-024-02875-1

Locoregional delivery of IL-13Rα2-targeting CAR-T cells in recurrent high-grade glioma: a phase 1 trial

In the format provided by the authors and unedited

Supplementary Information

Supplementary Fig. 1. Schema of CAR-T cell manufacturing process.

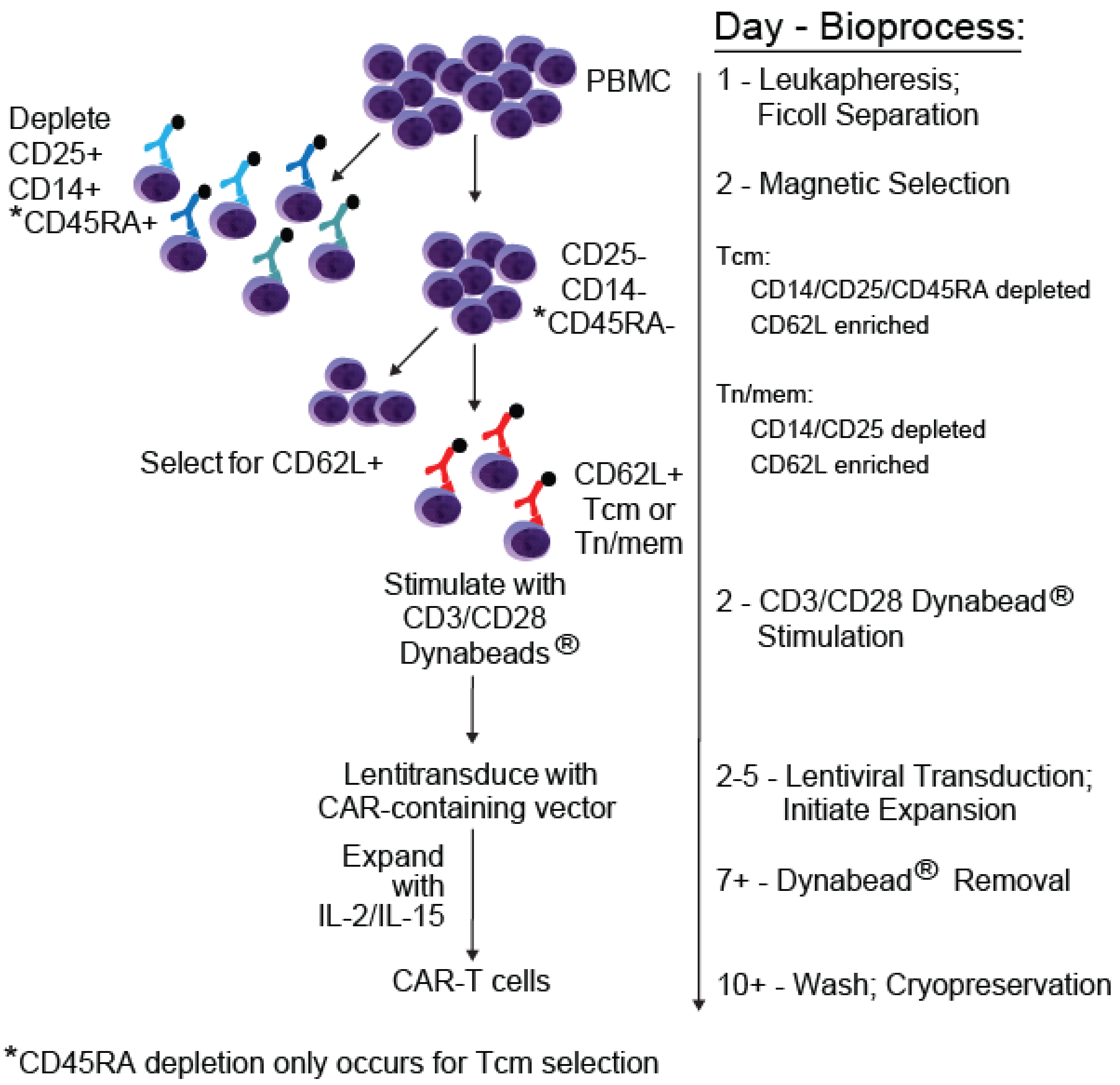

Supplementary Fig. 2. Flow cytometric gating strategies. a, PBMC or CAR-T cell products were gated first to exclude debris by FSC vs SSC (left) then for viability using DAPI (middle), and, when indicated, further for the CD3+ population (right). **b**, For Treg analyses, CAR-T cell products were gated first for viability, then for the CD3+ population, then for the CD4+ population, and finally for the CAR/CD19t+ population. **c**, Cells in the CSF/TCF were gated first to exclude debris by FSC vs SSC (left) then for viability using DAPI (middle), and then further for the CD3+ population (right).

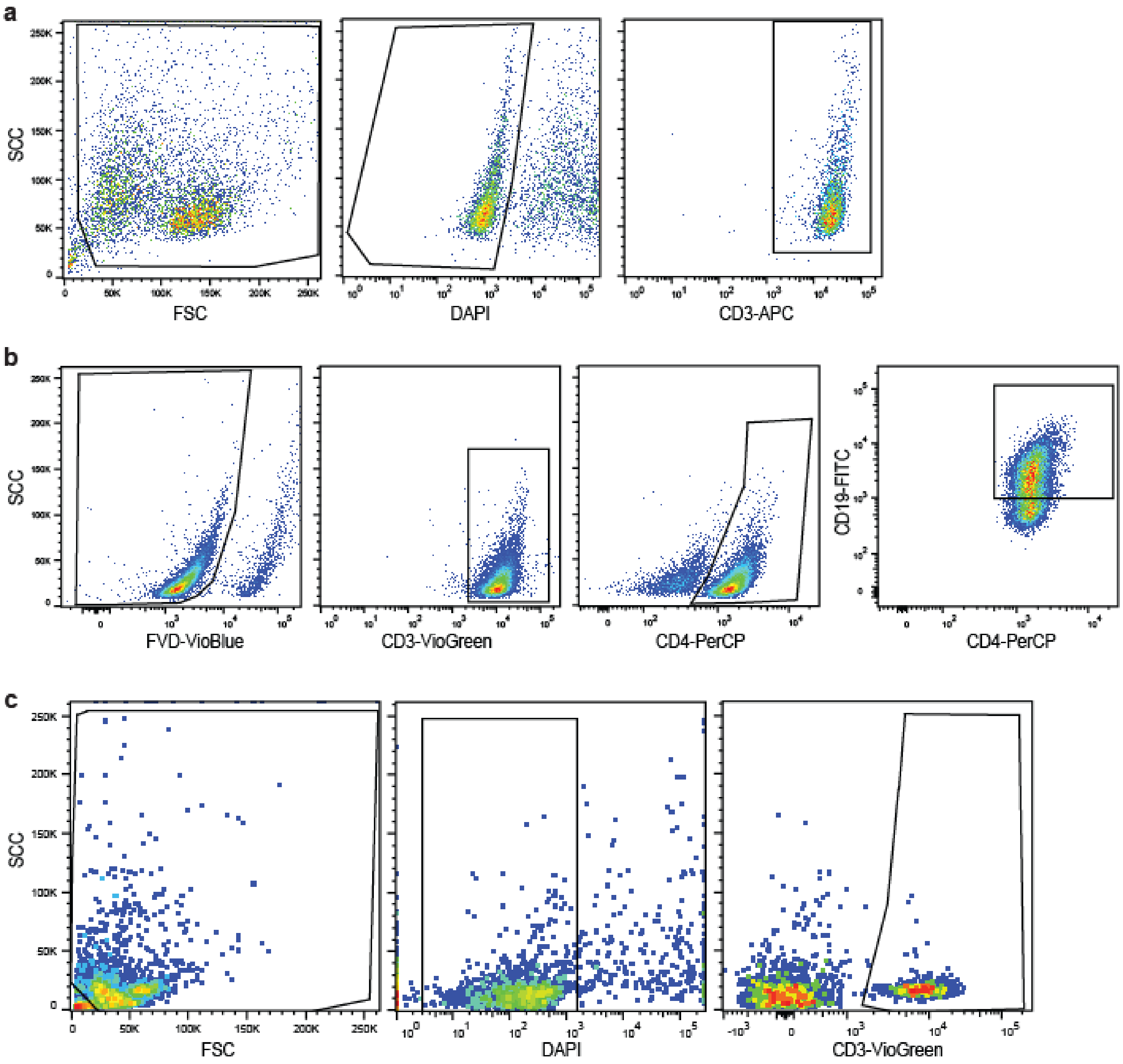

Supplementary Fig. 3. Preclinical evaluation of Tcm- vs. Tn/mem-derived CAR-T cells. a, Flow cytometric phenotype of healthy donor Tcm- and Tn/mem-derived CAR-T cell products. **b**, NSG mice were engrafted with Raji tumor cells (0.5x10^{6}), treated with intravenous (IV) delivery of 1 million (M) CD19-targeting CAR+ T cells 3 days later, and then followed for survival. *P* = 0.0002 when comparing those treated with Tcm- vs. Tn/mem-derived CAR-T cells using the log rank (Mantel-Cox) test. **c**, NSG mice were intracranially (IC) engrafted with human primary brain tumor (PBT) cells (0.1x10^{6}), treated with intratumoral (ICT) delivery of 0.1 M mock-transduced (Mock) or IL13Rα2-targeting CAR+ T cells, and then followed for survival. *P* = 0.15 when comparing those treated with Tcm- vs. Tn/mem-derived CAR-T cells using the log rank (Mantel-Cox) test.

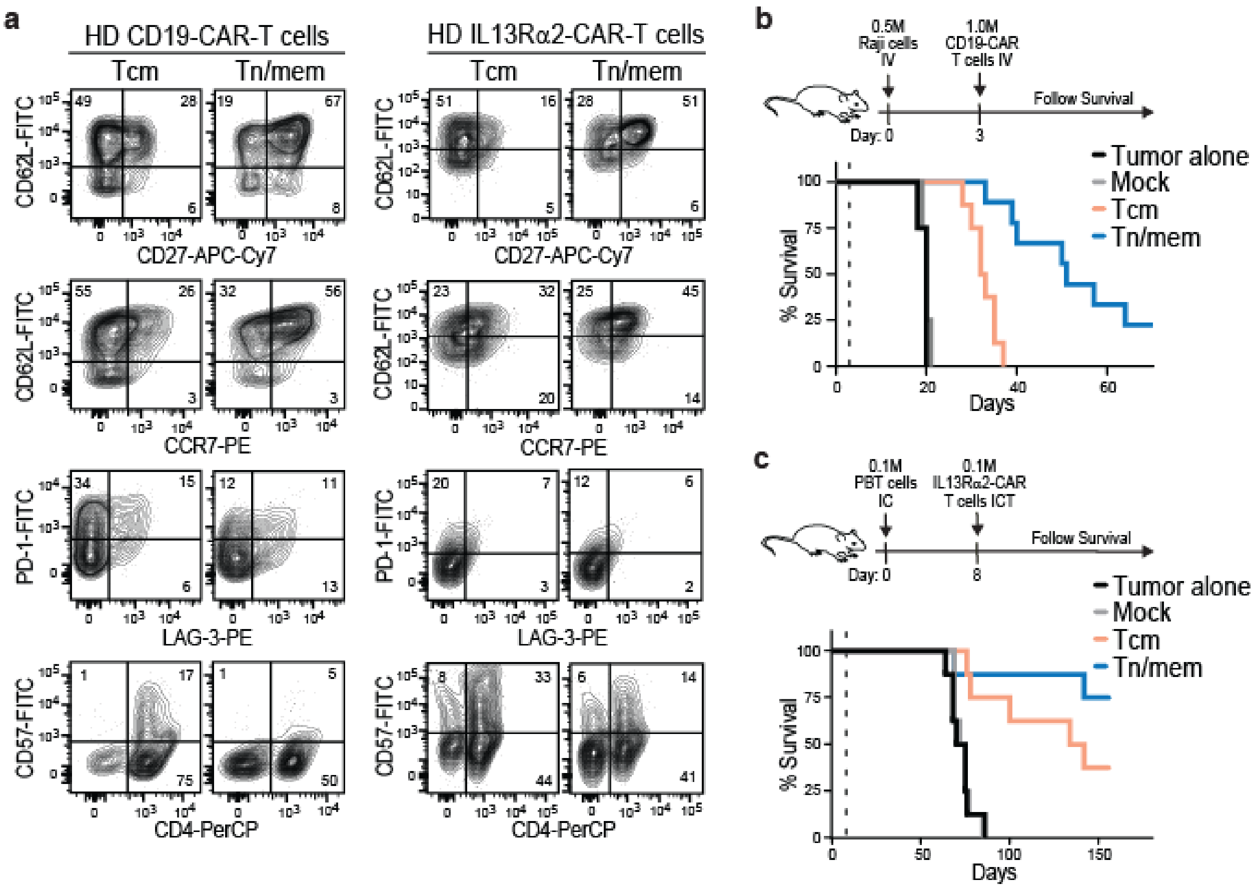

Supplementary Fig. 4. scRNA sequencing analyses. Heat map and unsupervised clustering of gene expression in the infusion products of 62 enrolled patients. Log2-normalized, scaled and quantile-normalized expression of 247 RNA features (rows) – including 89 canonical T cell markers, markers that maximally separate the 12 clusters, and markers differentiating Tn/mem- and Tcm-derived CAR-T cell products – across a random subset of 30,000 CAR-T product cells (columns). Cluster, batch, patient outcome, cell cycle phase, the percentage of reads mapping to mitochondrial (MT) genes, as well as memory-like and dysfunctional gene signature scores are indicated at the top. 42 canonical T cell markers included in the gene signature analysis are labeled at right. Rows are clustered at left based on Euclidean distances with k=10.

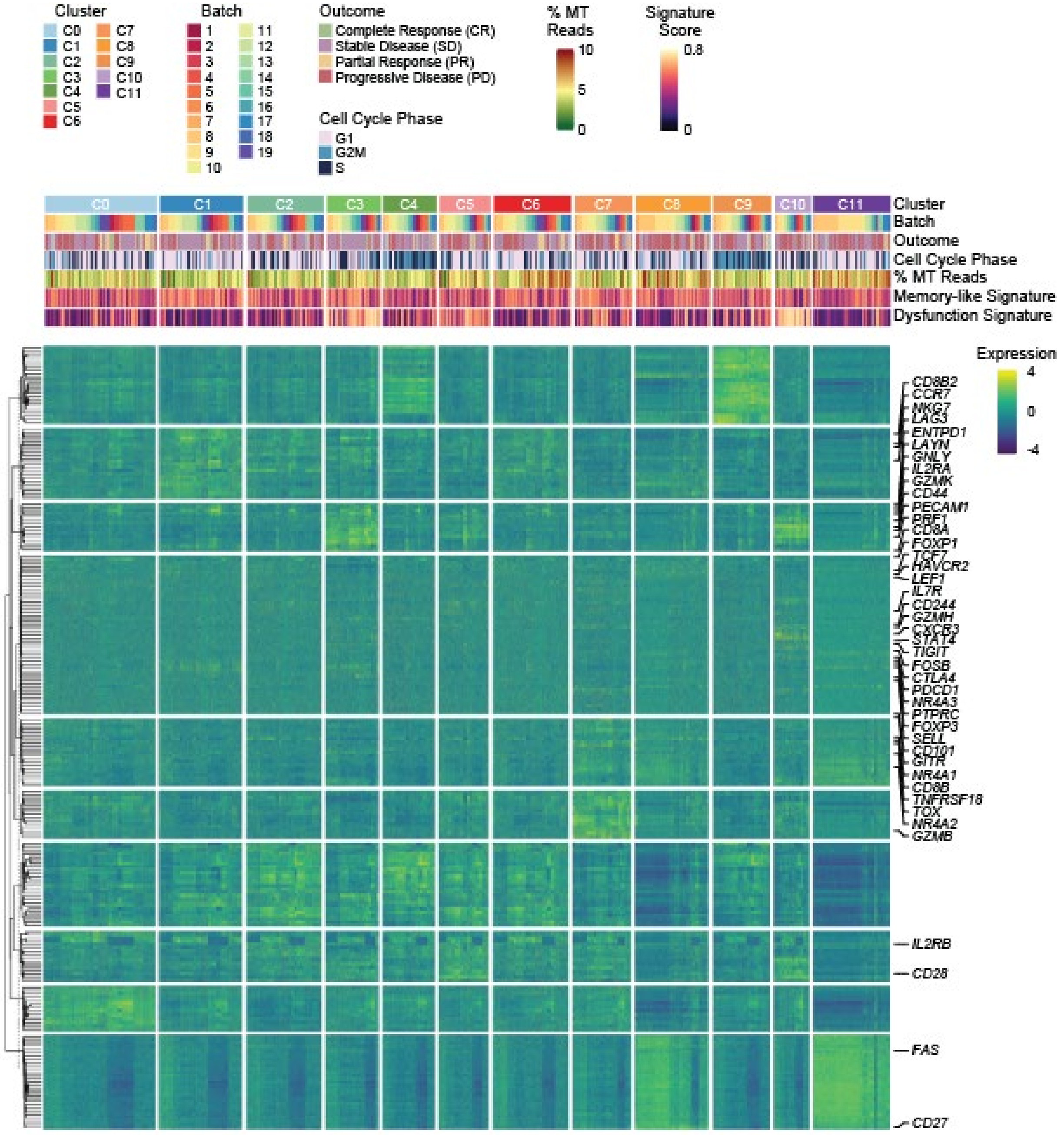

Cluster
C0
C1
C2
C3
C4
C5
C6
C7
C8
C9
C10
C11
Batch
1
2
3
4
5
6
7
8
9
10
11
12
13
14
15
16
17
18
19
Outcome
Complete Response (CR)
Stable Disease (SD)
Partial Response (PR)
Progressive Disease (PD)
Cell Cycle Phase
G1
G2M
S
% MT Reads
10
5
0
Signature Score
0.8
0
Cluster
Batch
Outcome
Cell Cycle Phase
% MT Reads
Memory-like Signature
Dysfunction Signature
Expression
4
0
-4
CD8B2
CCR7
NKG7
LAG3
ENTPD1
LAYN
GNLY
IL2RA
GZMK
CD44
PECAM1
PRF1
CD8A
FOXP1
TCF7
HAVCR2
LEF1
IL7R
CD244
GZMH
CXCR3
STAT4
TIGIT
FOSB
CTLA4
PDCD1
NR4A3
PTPRC
FOXP3
SELL
CD101
GITR
NR4A1
CD8B
TNFRSF18
TOX
NR4A2
GZMB
IL2RB
CD28
FAS
CD27

Supplementary Fig. 5. Summary of patient samples available for analyses. Graphs depicting CSF (top), TCF (middle) and serum (SER; bottom) samples available for evaluation and broken down by infusion cycle (y-axis) and patient (UPN; x-axis) on each treatment arm. Blocks are shaded dark if both pre-treatment (day 0) and post-treatment (day 1 or 2) samples were available at any given cycle. Blocks were shaded lighter if there is either a pre-treatment or post-treatment sample (not both). For some patients, the TCF catheter communicated with the CSF, which enabled TCF to be collected during later treatment cycles.

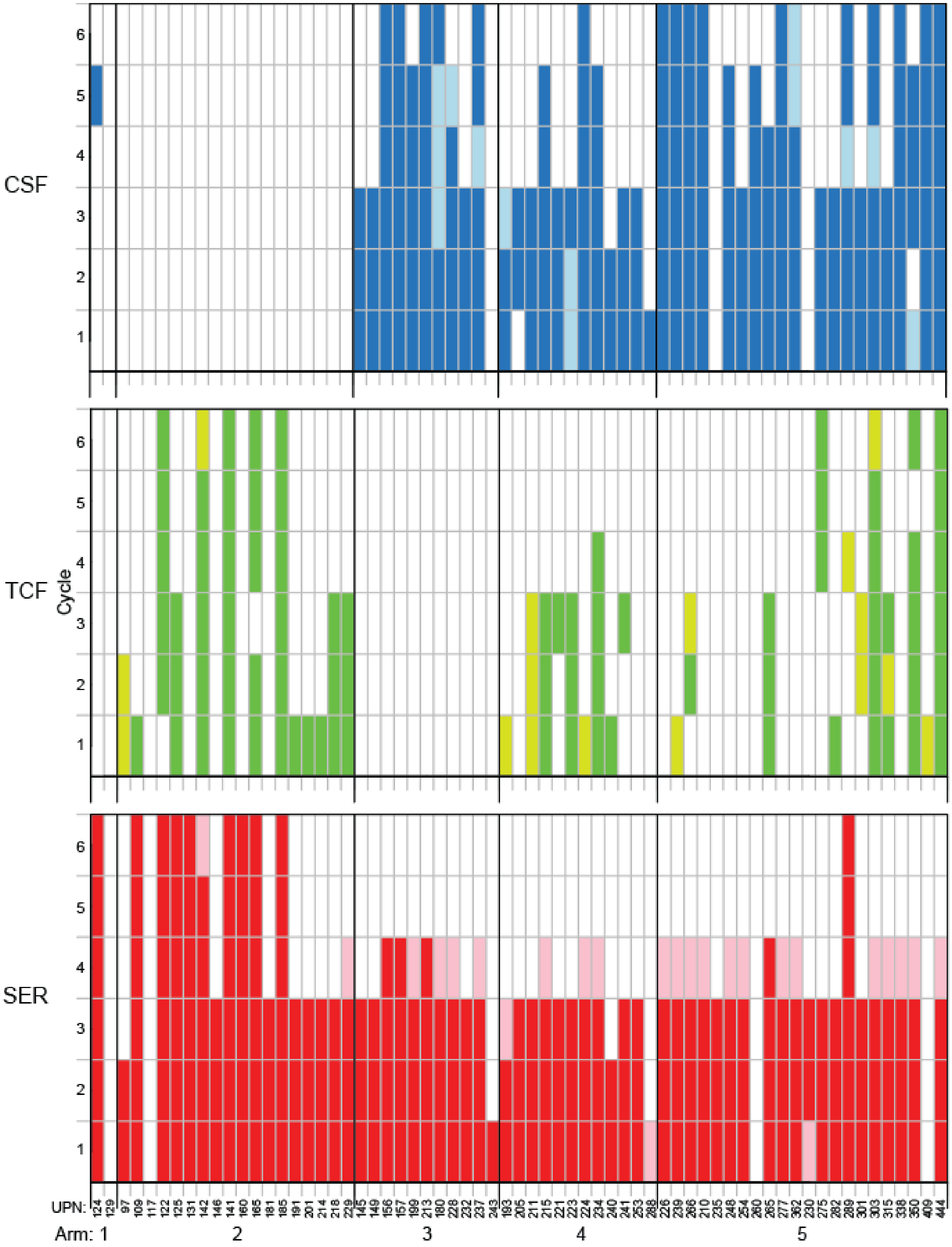
CSF
TCF
SER
Cycle
1
2
3
4
5
6
UPN:
Arm: 1
2
3
4
5

Supplementary Fig. 6. CAR-T cell persistence analysis by dose. Box and whisker plots depict AUC for number of CAR-T cells per µL in the CSF from cycles 1, 2 and 3 (**a**) or maximum WPRE copy number per µg of PBMC DNA (**b**) by arm and dose schedule. The median and interquartile range with whiskers extending to the minimum and maximum values are depicted.

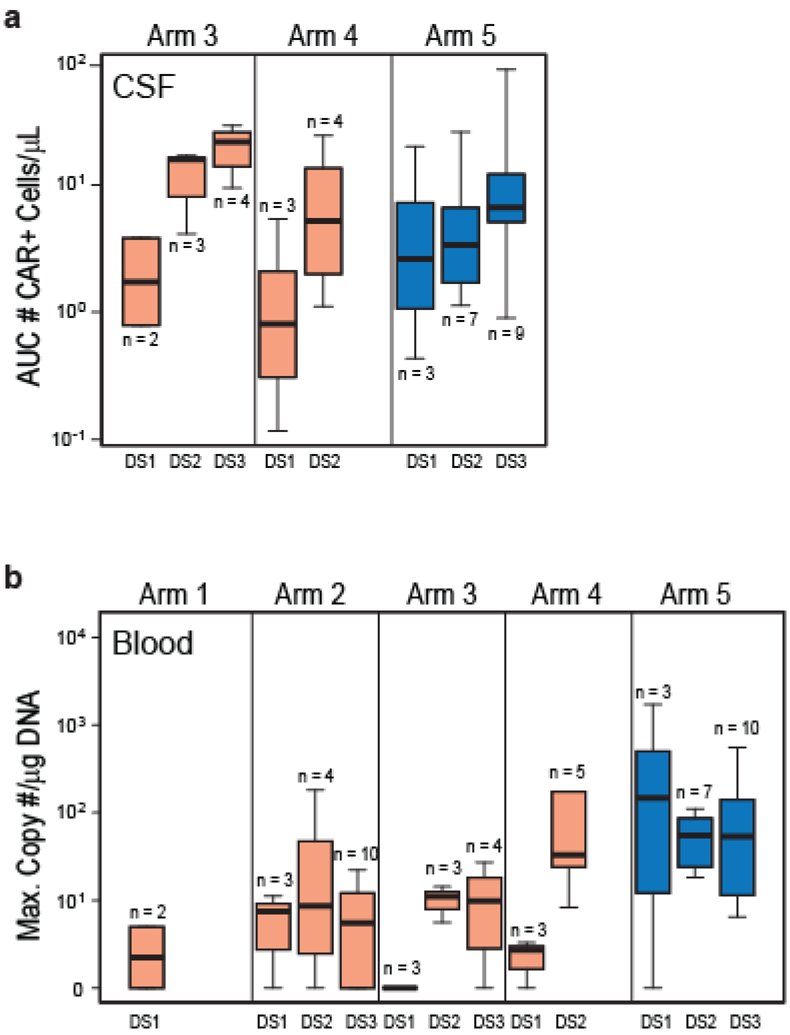

Supplementary Fig. 7. CD3 staining of patients' tumors. Representative CD3 IHC staining patterns of tumors at time of surgery, from each patient that is evaluable for survival, and divided into those that were qualitatively scored as high (**a**), intermediate (**b**) or negative/low (**c**). UPN numbers are provided for each image, and bars in first image of each panel indicate 50 μm. Qualitative scoring for CD3 was as follows: tumors with few detectable T cells were given a CD3 IHC score of 1; those with scattered CD3+ cells throughout (e.g., UPN224), with possible occasional perivascular aggregates or hot spots (e.g., UPN181) were given a score of 2; those with many CD3+ cells throughout and dense hot spots were given a score of 3 (**b**); and those with many dense CD3+ cell infiltrates were given a score of 4 (**a**).

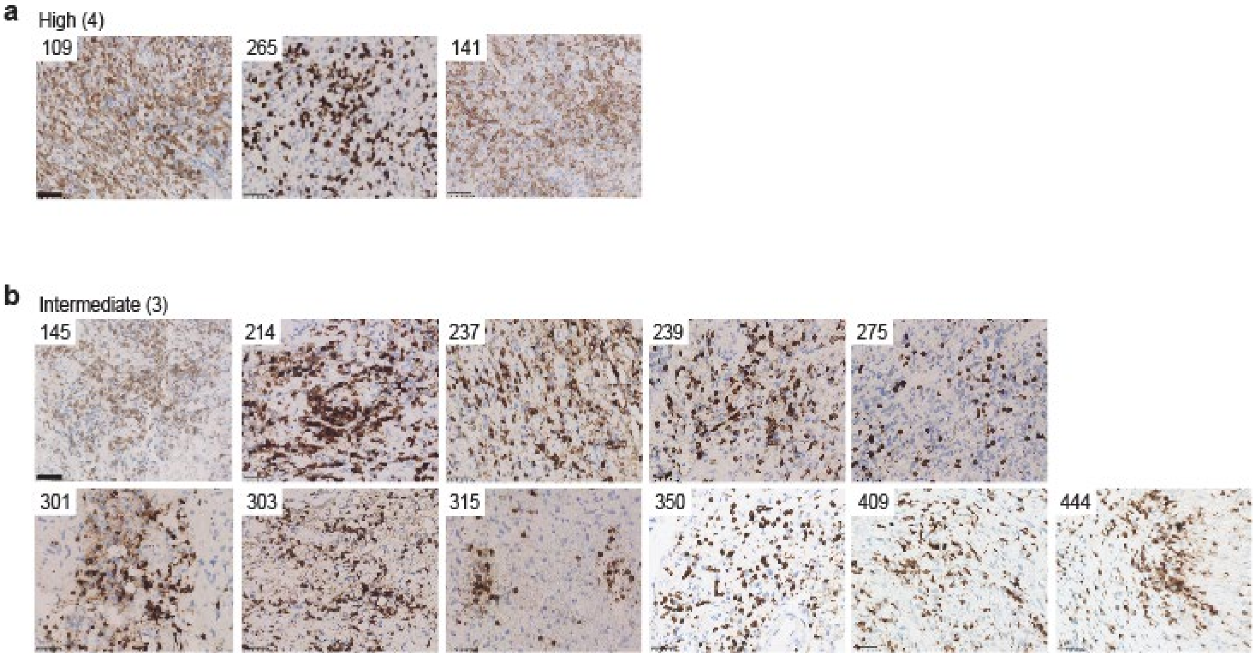

c

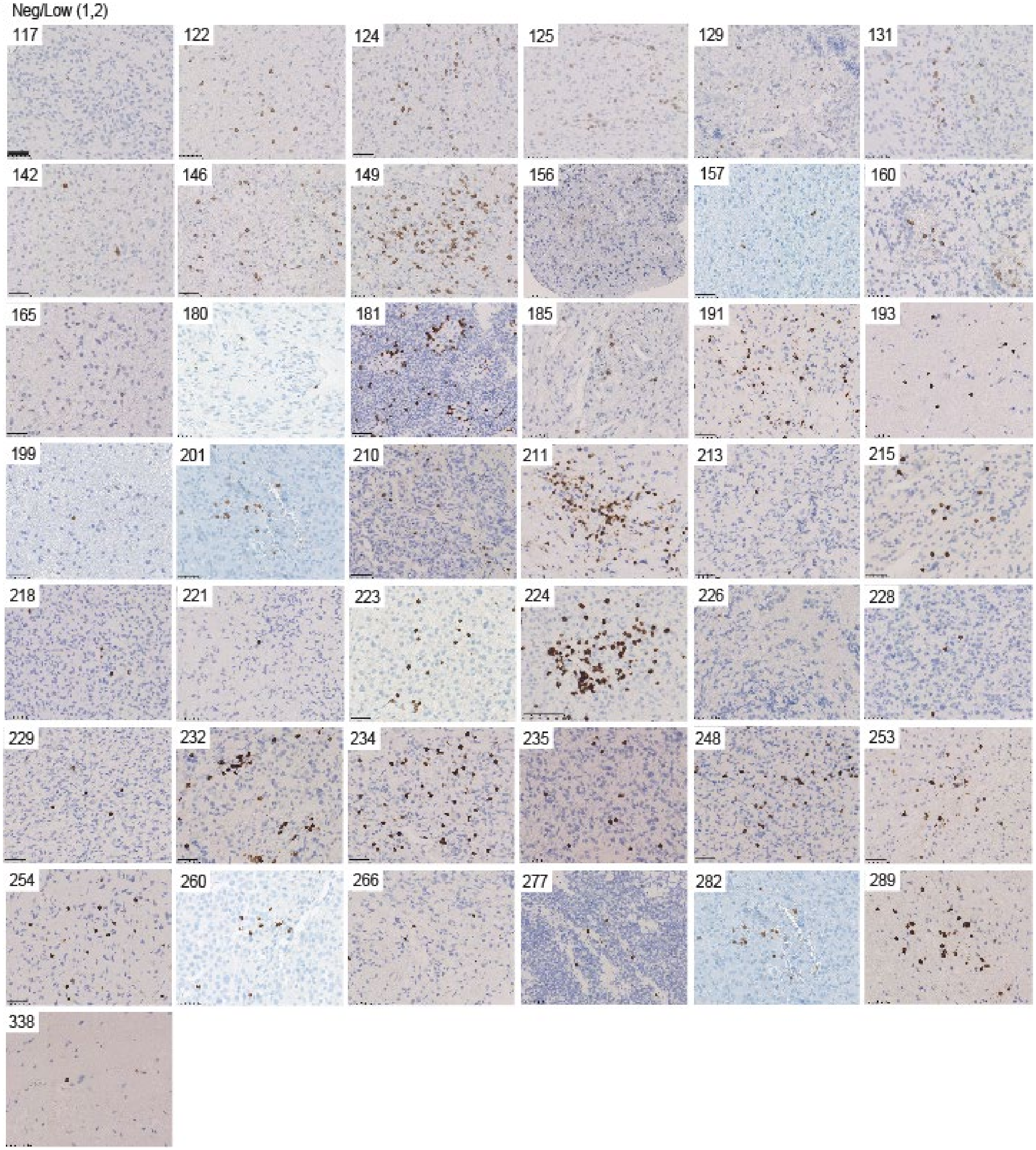
Neg/Low (1,2)
117
122
124
125
129
131
142
146
149
156
157
160
165
180
181
185
191
193
199
201
210
211
213
215
218
221
223
224
226
228
229
232
234
235
248
253
254
260
266
277
282
289
338

Supplementary Fig. 8. Tumor contexture evaluation by immunohistochemistry. a, Representative IHC staining patterns of UPN109 tumors at time of surgery (pre-treatment at left; CD3 high) and at first recurrence after IL13Rα2-CAR-T therapy (post-treatment at right; CD3 low). Top images, CD3 staining depicted in brown. Bottom images, 4-Plex staining shows CD4 staining in teal; CD8 staining in red/magenta; CD66b staining in purple (granulocytes); and CD68 staining in yellow (macrophages/monocytes/microglia). **b**, Representative IHC staining patterns of pre-treatment UPN265 (left) and UPN301 (right) tumors. Top images, CD8 staining depicted in brown. Bottom images, 4-Plex staining shows CD4 staining in teal; CD8 staining in red/magenta; CD66b staining in purple (granulocytes); and CD68 staining in yellow (macrophages/monocytes/microglia). **c**, IHC staining for Treg cells in pretreatment UPN109 (left), UPN265 (middle) and UPN301 (right) tumors. CD3 staining depicted in green, CD4 staining depicted in purple, FOXP3 staining depicted in brown. **d**, Box and whisker plots of cell counts (Log10) of CD3+, CD8+ or FOXP3+ cells quantified from the IHC stained tumor sections for tumors with CD3 score of negative/low (1,2) (n=8 tumors), intermediate (3) (n=9 tumors), or high (4) (n=2 tumors). The median and interquartile range with whiskers extending to the minimum and maximum values are depicted. *, $P \leq 0.027$; and **, $P = 0.0078$ using a two-sided Mann-Whitney test, and paired for cell types within a given tumor. **e**, Estimated survival effect of switching treatment from Tcm to Tn/mem products sub-analyzed by tumor CD3 score: CD3-high (3,4) and CD3-low (1,2) for survival evaluable rGBM patients (n = 41). Dots give point estimates of the mean effect, bars represent 95% confidence intervals. Findings show that the survival effect for hot tumors is not statistically different when switching from Tcm to Tn/mem. By contrast for cold tumors, moving from Tcm to Tn/mem products is estimated to have an approximate 1.8-fold benefit for survival. Reference methods for statistical analysis details.

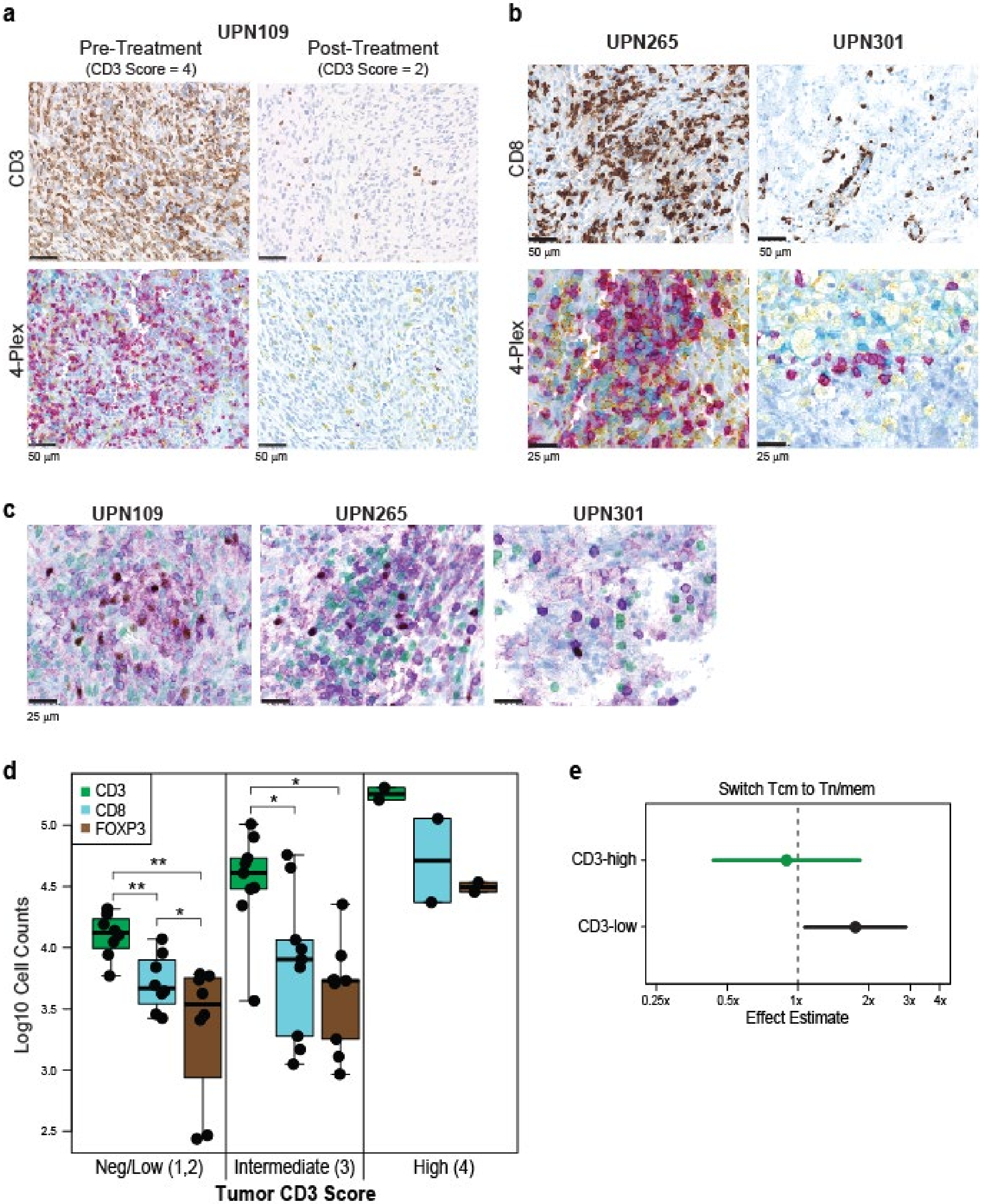
a
UPN109
Pre-Treatment
(CD3 Score = 4)
Post-Treatment
(CD3 Score = 2)
CD3
4-Plex
50 μm
50 μm
b
UPN265
UPN301
CD8
4-Plex
50 μm
50 μm
25 μm
25 μm
c
UPN109
UPN265
UPN301
25 μm
d
CD3
CD8
FOXP3
Log10 Cell Counts
5.0
4.5
4.0
3.5
3.0
2.5
**
**
*
*
*
Neg/Low (1,2)
Intermediate (3)
High (4)
Tumor CD3 Score
e
Switch Tcm to Tn/mem
CD3-high
CD3-low
0.25x
0.5x
1x
2x
3x
4x
Effect Estimate

CITY OF HOPE NATIONAL MEDICAL CENTER
1500 E. DUARTE ROAD
DUARTE, CA 91010

DEPARTMENT OF HEMATOLOGY/ HEMATOPOEITIC CELL TRANSPLANTATION
&
DIVISION OF NEUROSURGERY

TITLE: PHASE I STUDY OF CELLULAR IMMUNOTHERAPY USING MEMORY ENRICHED T CELLS LENTIVIRALLY TRANSDUCED TO EXPRESS AN IL13Rα2-SPECIFIC, HINGE-OPTIMIZED, 41BB-COSTIMULATORY CHIMERIC RECEPTOR AND A TRUNCATED CD19 FOR PATIENTS WITH RECURRENT/REFRACTORY MALIGNANT GLIOMA

**SPONSOR/IND NUMBER:**	**BB IND 16226**
**DISEASE SITE:**	Brain
**STAGE (if applicable):**	N/A
**MODALITY:**	Intracavitary/Intratumoral/Intraventricular Administration of Cellular Immunotherapy Using Autologous IL13(EQ)BBζ/CD19t+ T cells
**PHASE/TYPE:**	Phase I/Safety

PRINCIPAL INVESTIGATOR

Behnam Badie, M.D.
City of Hope National Medical Center
Department of Neurosurgery
T: 626-456-4673 x87100
Email: bbadie@coh.org

Experimental Design Schema

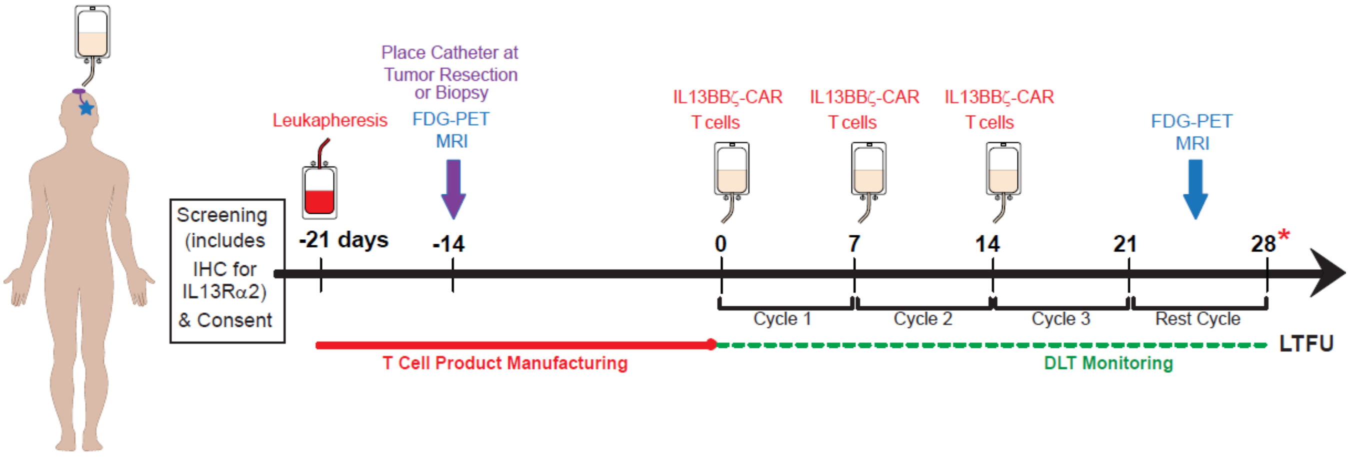

*Optional additional cycles of CAR T cell infusions may be initiated after day 28.

Table 13.1: Dose Schedule (CAR+) for Arms 1 to 5^						
Planned Cycle	**Dose schedule 1de**	**Dose schedule 1**	**Dose schedule 2de**	**Dose schedule 2**	**Dose Schedule 3de**	**Dose schedule 3**
1	A: 2x10^6	A: 2x10^6	C: 10x10^6	C: 10x10^6	C:10x10^6	F: 20x10^6
2	B: 5x10^6	C: 10x10^6	D: 25x10^6	E: 50x10^6	G:75x10^6	H:100x10^6
3	B: 5x10^6	C: 10x10^6	D: 25x10^6	E: 50x10^6	G:75x10^6	H:100x10^6
Total Dose	*12x10^6*	*22x10^6*	*60x10^6*	*110x10^6*	*160x10^6*	*220x10^6*
	Evaluation/Restaging					
Optional Cycle#	≤5x10^6	≤10x10^6	≤25x10^6	≤50x10^6	≤75x10^6	≤100x10^6

^ Infusion doses should not exceed 2.5x10^8 per 0.5mL (0.5x10^6/µL); a dose schedule consists of a participant receiving 3 infusions. Reference **Section 5.2.1**.

de = de-escalation schedule

The dose listed at each cycle is the CAR+ T cell dose intended for each delivery method, therefore in Arms 4 (T_{CM} Dual Delivery) and 5 ($T_{N/MEM}$ Dual Delivery) 2 infusions doses will be prepared per infusion cycle, 1 for each delivery method.

Protocol Synopsis

**Protocol Title:**
Phase I Study of Cellular Immunotherapy using Memory Enriched T Cells Lentivirally Transduced to Express an IL13Rα2-specific, Hinge-optimized, 41BB-costimulatory Chimeric Receptor and a Truncated CD19 for Patients with Recurrent/Refractory Malignant Glioma
**Brief Protocol Title for the Lay Public (if applicable):**
IL13(EQ)BBζ/CD19t+ T cells for the treatment of malignant glioma
**Study Phase:**
Phase I
**Participating Sites:**
Not applicable
**Rationale for this Study:**
The treatment of high-grade malignant glioma (MG), including anaplastic astrocytoma (AA-grade III) and glioblastoma multiforme (GBM-grade IV), remains a significant therapeutic challenge. Currently available therapeutic options have limited curative potential and only less than 5% of patients survive more than five years after initial diagnosis. Adoptive cellular immunotherapy (ACIT) utilizing chimeric antigen receptor (CAR) engineered T cells may provide a safe and effective way to reduce recurrence rates of MG, since CAR T cells can be engineered to specifically recognize antigenically-distinct tumor populations,[1] and T cells can migrate through the brain parenchyma to target and kill infiltrative malignant cells.[2-4] We have developed a CAR T cell immunotherapy for MG targeting IL-13 receptor α2 (IL13Rα2), a cell surface receptor over-expressed by a high percentage of MGs, but not expressed at significant levels on normal brain tissue. Pre-clinical studies have demonstrated that IL13Rα2-targeting CAR+ T cells exhibit potent MHC-independent, IL13Rα2-specific cytolytic activity against both stem-like and differentiated glioma cells, and induce regression of established glioma xenografts *in vivo*.[5,6] Our experience with two FDA-authorized clinical trials evaluating intracranial administration of both autologous and allogeneic IL13Rα2-targeting CAR+ T cell clones in 9 research participants with recurrent/refractory glioblastoma has demonstrated the feasibility of this approach, the absence of serious therapy-related side-effects, and has provided evidence for transient anti-glioma responses for patients with IL13Rα2-expressing MG.<br><br>The phase I clinical study proposed here now aims to build on this clinical experience by examining the feasibility and safety of intracavitary/intratumoral/intraventricular administration of autologous *ex vivo* expanded central memory-enriched T cells (T_{CM}) that are genetically modified using a self-inactivating (SIN) lentiviral vector to express an IL13Rα2-specific, hinge-optimized, 41BB-costimulatory chimeric antigen receptor (CAR) as well as a truncated human CD19 (IL13(EQ)BBζ/CD19t+ T_{CM} or $T_{N/MEM}$) to patients with recurrent or refractory MG. The use of T_{CM} or $T_{N/MEM}$ for genetic modification is based on studies using both murine xenograft models and a non-human primate model relevant for human translation,[7,8] which suggest that autologous IL13(EQ)BBζ/CD19t+ T_{CM}-derived effectors will retain their memory phenotype for persistence after their genetic modification and *ex vivo* propagation, and, upon adoptive transfer, will produce robust anti-tumor responses against IL13Rα2+ MG.<br><br>In this protocol, research subjects with documented IL13Rα2+ recurrent/refractory high-grade glioma (Grades III and IV) will be enrolled and undergo PBMC collection. Concurrent with the manufacturing process to generate IL13(EQ)BBζ/CD19t+ T_{CM} or $T_{N/MEM}$, research participants will undergo biopsy or resection of their tumor, as applicable, followed by placement of a Rickham catheter and baseline imaging. Following recovery from surgery, research participants will then

receive an initial low dose (cycle 1) followed by 2 additional infusions at the 5-fold higher cell dose (cycles 2 and 3) of autologous IL13(EQ)BBζ/CD19t+ T_{CM} or $T_{N/MEM}$, potentially followed by additional cycles at up to the highest tolerated cell dose (cycles 4+). T cells will be administered in one of four ways: (1) directly into the tumor (Arm 1 = intratumoral), (2) into the tumor resection cavity (Arm 2 = intracavitary), (3) into the lateral ventricles (Arm 3 = intraventricular), or dual delivery (Arm 4= both intratumoral [includes delivery in to either the tumor OR tumor resection cavity] and intraventricular). Arm 5 ($T_{N/MEM}$ Dual Delivery) will investigate autologous IL13(EQ)BBζ/CD19t+ $T_{N/MEM}$ administered via Dual Delivery.

Objectives:

The *Primary Objectives* are:

- Assess the feasibility and safety of cellular immunotherapy utilizing *ex vivo* expanded autologous memory-enriched T cells that are genetically modified using a self-inactivating (SIN) lentiviral vector to express a IL13Rα2-specific, hinge-optimized, 41BB-costimulatory CAR, as well as a truncated human CD19 for participants with recurrent/refractory malignant glioma in one of the following ways: Arm 1 (intratumoral delivery of IL13(EQ)BBζ/CD19t+ T_{CM}), Arm 2 (intracavitary delivery of IL13(EQ)BBζ/CD19t+ T_{CM}), Arm 3 (intraventricular delivery of IL13(EQ)BBζ/CD19t+ T_{CM}), Arm 4 (dual delivery [both intratumoral and intraventricular] of IL13(EQ)BBζ/CD19t+ T_{CM}) or Arm 5 (dual delivery [both intratumoral and intraventricular] of IL13(EQ)BBζ/CD19t+ $T_{N/MEM}$), and
- Determine maximum tolerated dose schedule (MTD)/maximum feasible dose schedule (MFD) and a recommend Phase II dosing plan (RP2D) for each arm based on dose limiting toxicities (DLTs) and the full toxicity profile.

The *Secondary Objectives* are

- In research participants who receive the full schedule of 3 CAR T cell doses:
  - Estimate disease response rates,
  - Estimate median overall survival, and
  - Estimate the mean change from baseline in quality of life using the EORTC QLQ-C30 during and post treatment;
- Describe cytokine levels (CSF, tumor cavity fluid, peripheral blood) over the study period;
- Describe CAR T cell and endogenous immune populations (CSF, tumor cavity fluid, peripheral blood) over the study period; and
- Identify tumor and tumor micro-environment markers associated with response to CAR T cells.

The *Exploratory Objectives* are

- Assess the timing and extent of brain inflammation following CAR T cell administration;
- Evaluate CAR T cell product characteristics; and
- For research participants who undergo a second resection or autopsy:
  - Evaluate CAR T cell persistence in the tumor micro-environment and the location of the CAR T cells with respect to the injection site, and
  - Evaluate IL13Rα2 antigen expression levels pre and post CAR T cell therapy

Study Design:

This protocol is a Phase I feasibility/safety study of IL13(EQ)BBζ/CD19t+ T cells which implements both inter- and intra-patient dose escalation in five patient arms (T_{CM} intratumoral, intracavity, intraventricular, and dual delivery [both intratumoral/intracavity and

intraventricular], as well as $T_{N/MEM}$ dual delivery [both intratumoral/intracavity and intraventricular]) for recurrent/refractory malignant glioma.
**Endpoints:**
Primary: Grade 3 toxicities, DLTs, and all other toxicities.<br>Secondary: CAR T cell and cytokine levels in peripheral blood, CSF, and tumor cavity fluid; disease response by RANO criteria; endogenous immune cell detection; overall survival; scale, domain, and item scores from the EORTC QLQ-C30; and tumor/tumor micro-environment marker detection.<br>Exploratory: Timing and extent of brain inflammation; CAR T cell product characteristics; Persistence and location of CAR T cells in tumor micro-environment; and IL13Rα2 antigen expression levels.
**Sample Size:**
As of 03/02/2018, we have closed Arm 1 (biopsy with an N=2) as biopsy participants can be included in the ICV or dual arms. Based on simulation results, we expect to study 15 participants in the dose escalation portion of the trial, plus 6 in the expansion portion plus 2 for replacement of unevaluable participants, giving an expected sample size of 23 per arm for each of the 4 open arms for a total of 92.
**Estimated Duration of the Study**
Accrual is expected to be 6 evaluable research participants per year per arm. Thus, we expect to complete accrual in 5years.
**Summary of Subject Eligibility Criteria:**
<u>Inclusion Criteria:</u><br>1. Participant has a prior histologically-confirmed diagnosis of a grade III or IV glioma, or has a prior histologically-confirmed diagnosis of a grade II glioma and now has radiographic progression consistent with a grade III or IV MG after completing standard therapy.<br>2. Radiographic evidence of recurrence/progression recurrence of the measurable disease more than 12 weeks after the end of the initial radiation therapy.<br>3. COH Clinical Pathology confirms IL13Rα2+ tumor expression by immunohistochemistry (≥ 20%, 1+).<br>4. Male or female participants between 12 and 75 years of age at time of screening.<br>5. KPS ≥60%<br>6. Life expectancy > 4 weeks<br>7. Negative serum pregnancy test for women of childbearing potential<br>8. Informed Consent/Assent: All subjects must have the ability to understand and the willingness to sign a written informed consent<br><u>Exclusion Criteria:</u><br>1. Failure of research participant to understand the basic elements of the protocol and/or the risks/benefits of participating in this phase I study. A legal guardian may substitute for the research participant.<br>2. Basic organ function testing as follows:<br>• <u>Pulmonary</u>: Research participant requires supplemental oxygen to keep saturation greater than 95% and the situation is not expected to resolve within 2 weeks.<br>• <u>Cardiac</u>: Research participant requires pressor support and/or has symptomatic cardiac arrhythmias.

• Renal: Research participant requires dialysis.<br>• Neurologic: Research participant has uncontrolled seizure activity and/or clinically evident progressive encephalopathy.<br>3. Research participants with any non-malignant intercurrent illness which is either poorly controlled with currently available treatment, or which is of such severity that the investigators deem it unwise to enter the research participant on protocol shall be ineligible<br>4. Research participants with any other active malignancies.<br>5. Research participants being treated for severe infection or who are recovering from major surgery are ineligible until recovery is deemed complete by the investigator<br>6. Research participants with any uncontrolled illness including ongoing or active infection. Research participants with known active hepatitis B or C infection; research participants with any signs or symptoms of active infection, positive blood cultures or radiological evidence of infections.<br>7. Research participants who have confirmed HIV positivity within 4 weeks of screening.
**Investigational Product Dosage and Administration:**
Autologous IL13(EQ)BBζ/CD19t+ T cells will be administered through a Rickham catheter either (1) directly into the tumor (Arm 1 = intratumoral), (2) into the tumor cavity (Arm 2 = intracavitary), (3) into the lateral ventricles (Arm 3 = intraventricular), or (4) into both the tumor/tumor cavity (intratumoral) and into the lateral ventricles (intraventricular) (Arms 4 and 5 = dual delivery). Research participants will receive an initial low dose (cycle 1) followed by 2 additional infusions at the 5-fold higher cell dose (cycles 2 and 3) of autologous IL13(EQ)BBζ/CD19t+ T_{CM} or $T_{N/MEM}$, potentially followed by additional cycles at up to the highest tolerated cell dose (cycles 4+) as long as they continue to meet eligibility criteria AND have doses available for infusion. Depending on the participant's disease status, T cells will be administered one of four ways: (1) directly into the tumor (Arm 1 = intratumoral), (2) into the tumor resection cavity (Arm 2 = intracavitary), (3) into the lateral ventricles (Arm 3 = intraventricular), or (4) into both the tumor/tumor cavity (intratumoral) and into the lateral ventricles (intraventricular) (Arm 4 = IL13(EQ)BBζ/CD19t+ T_{CM} dual delivery and Arm 5 = IL13(EQ)BBζ/CD19t+ $T_{N/MEM}$ dual delivery).
**Clinical Observations and Tests to be Performed:**
Clinical observation will include assessment of performance and relapse status, CTCAE grading, and comprehensive metabolic and chemistry profiles, and EORTC QLQ-C30. Peripheral blood, tumor cavity aspirate, and CSF samples (when available) will also be collected from research participants at designated time points (defined in **Section 10**) for the purposes of correlative studies that will include, but are not limited to the following: 1) Antitumor responses following CAR T cell therapy; 2) determination of the frequency of CAR+ T cells in the tumor microenvironment and peripheral blood; 3) *ex vivo* immunophenotyping and functional analysis of infused cells; 4) serum cytokine analysis; and 5) evaluation of tumor IL13Rα2 expression and susceptibility to CAR+ T cells.
**Statistical Considerations:**
The statistical analysis will be done separately for each arm. Rate and associated 95% Clopper and Pearson binomial confidence limits (95% CI) will be estimated for participants' experiencing DLTs at the RP2D schedule. In study participants that received the full schedule of 3 T cell doses, we will estimate the rate (95% CI) with disease response, and Kaplan Meier methods will be used to estimate median PFS and OS and graph the results. In study participants that receive at least 1 dose

of T cells we will estimate the mean and standard error for change from baseline during treatment and post treatment in the quality of life functioning scale, symptom scale and item scores from the EORTC QLQ-C30. Statistical and graphical methods will be used to describe persistence and expansion of the CAR T cells (peripheral blood, CSF, and tumor cavity fluid) and cytokine levels (CSF, tumor cavity fluid, peripheral blood) over the study period. In study participants that undergo a second resection or following autopsy, T cells numbers, location, and antigen levels will be described. Tables will be created to summarize all toxicities and side effects by dose, time post treatment, organ, severity and arm. We will provide descriptive statistics for patient demographics.
**Sponsor/Licensee:**
City of Hope
**Case Report Forms**
Electronic Case Report Forms (eCRFs)<br>All data will be collected using study designed electronic case report forms (eCRFs)<br><br>*Administrative Forms (Numbering to be Assigned)*<br>UPN Assignment, DAC Leukapheresis Instructions and Requisition for T Cell Infusion.

Table of Contents

SECTION .... **PAGE**

Experimental Design Schema 2

Protocol Synopsis 3

1.0 Goals and Objectives (Scientific Aims) 15

1.1 Objectives: 15

1.1.1 *Primary Objectives*: 15

1.1.2 *Secondary Objective*: 15

1.1.3 *Exploratory Objectives* 15

2.0 Background 16

2.1 Introduction/Rationale for Development 16

2.1.1 Glioblastoma Multiforme 16

2.1.2 Treatment Options for GBM 16

2.1.3 IL13Rα2 expression and GBMs: 16

2.1.4 Adoptive Cellular Immunotherapy 16

2.1.5 Immunotherapy of Glioblastoma with Gene Modified T Cells 17

2.1.6 Route of Administration and Dosing. 19

2.2 Overview of Proposed Study 20

2.3 Preclinical Studies 21

2.3.1 IL13(EQ)BBζ/CD19t$^+$ T_{CM}. 21

2.4 Human Studies 23

2.4.1 Clinical ACIT Experience at COH Using Genetically Modified T cells (CAR T cells). 23

3.0 Patient Eligibility 26

3.1 Screening Inclusion Criteria 26

3.1.1 Disease Status 26

3.1.2 Age Criteria, Performance Status and Life Expectancy 27

3.1.3 Child Bearing Potential 27

3.1.4 Protocol-Specific Criteria 27

3.2 Screening Exclusion Criteria 28

3.2.1 Organ Function: 28

3.2.2 Study-Specific Exclusions 28

3.2.3 Non-Compliance 29

3.3 Inclusion of Women and Minorities 29

4.0 Screening and Registration Procedures 29

4.1 Screening Procedures 29
4.2 Informed Consent 29
4.3 Registration Requirements/Process 29
4.4 Randomization and/or Dose Level Assignment 30
5.0 Treatment Program 30
5.1 Treatment Overview 30
5.1.1 Schedule 30
5.2 Planned Duration of Therapy 33
5.3 Criteria for Removal from Treatment 34
5.4 Subject Follow-Up 36
5.4.1 Follow-Up Post CAR T cell Infusion 36
5.4.2 AE and ConMed data collection: 36
5.5 Supportive Care, Other Concomitant Therapy, Prohibited Medications 36
5.5.1 Supportive Care for Regimen Related Toxicities 36
5.5.2 Management of Constitutional Symptoms Associated with CAR T cell Infusion 37
5.5.3 Management of Acute Adverse Event(s) Attributable to This Study 37
5.6 Additional Studies 38
5.6.1 Laboratory Studies 38
5.6.2 Long-Term Follow-Up 38
5.7 Definition of Dose-Limiting Toxicity (DLT) 38
6.0 Dose Delays/Modifications for Adverse Events 39
7.0 Data and Safety Monitoring, Unanticipated Problems and Adverse Event Reporting 39
7.1 Data and Safety Monitoring 39
7.2 Monitoring and Personnel Responsible for Monitoring 39
7.3 Definitions 39
7.3.1 Adverse Events and Serious Adverse Events 39
7.3.2 Adverse event (AE): 39
7.3.3 Reporting Non-serious Adverse Events 39
7.3.4 Serious Adverse Event (SAE) **[**21 CFR 312.32**]:** 40
7.3.5 Reporting Serious Adverse Events 40
7.3.6 Adverse Event Name and Severity 40
7.3.7 Expected Adverse Event: 40
7.3.8 Unexpected Adverse Event [21 CFR 312.32 (a)]: 40
7.3.9 Adverse Event Attribution 40

7.3.10 COH Held IND 41
8.0 Agent Information and Risks 42
8.1 IL13(EQ)BBζ/CD19t+ T Cells (*BB-IND 16226*) 42
8.1.1 Description 42
8.1.2 Toxicology 43
8.1.3 Pharmacology – Handling, Storage, Dispensing and Disposal 44
8.2 ACTEMRA® (Tocilizumab) 45
8.2.1 Description: 45
8.2.2 Toxicology: 45
8.2.3 Pharmacology – Handling, Storage, Dispensing and Disposal 48
9.0 Correlative/Special Studies 48
9.1 Summary of Correlative Studies 48
9.1.1 Antitumor Responses Following Adoptive Transfer of IL13(EQ)BBζ/CD19t+ T Cells. 48
9.1.2 Determination of Frequency of IL13(EQ)BBζ/CD19t+ T Cells in the Tumor Microenvironment and Peripheral Blood 48
9.1.3 *Ex vivo* Immunophenotyping/Functional Analysis 48
9.1.4 Serum, CSF, and Tumor Cavity Cytokine Analysis 49
9.1.5 Evaluation of Tumor Antigen IL13α2 Expression and Susceptibility to Re-directed T Cells 49
9.1.6 Evaluation of human anti-CAR antibodies (HACA) 49
9.1.7 Evaluation of changes in frequency and phenotype of endogenous immune cell populations and tumor microenvironment 50
10.0 Study Calendar 51
10.1 Enrollment and Treatment Study Calendar: 51
10.2 Short-Term Follow-Up Calendar 54
10.3 Long-Term Follow-Up (or Modified Follow-Up for Participants that Progress):55
11.0 Endpoint Evaluation Criteria/Measurement of Effect 56
11.1 Response Criteria 56
12.0 Data Reporting/Protocol Deviations 56
12.1 Data Reporting 56
12.1.1 Confidentiality and Storage of Records 56
12.1.2 Subject Consent Form 56
12.1.3 Data Collection Forms and Submission Schedule 56
12.2 Protocol Deviations 57
12.2.1 Deviation Policy 57

12.2.2 Reporting of Deviations 57
12.2.3 Resolving Disputes 57
13.0 Statistical Considerations 57
13.1 Study Design 57
13.1.1 Study Participant Staggering Rules 58
13.2 Dose Escalation Rules (each arm will be followed separately): 58
13.2.1 Intra-patient dose escalation rules: 58
13.2.2 Inter-cohort dose escalation rules 59
13.3 Optional additional cycles 63
13.4 Study Endpoints: 64
13.5 Study Stopping Rules 64
13.5.1 Rules for Holding Accrual Due to Toxicity 64
13.6 Sample Size Accrual Rate 64
13.7 Statistical Analysis Plan 64
14.0 Human Subject Issues 65
14.1 Institutional Review Board 65
14.2 Recruitment of Subjects 65
14.3 Advertisements 65
14.4 Study location and Performance Sites 65
14.5 Confidentiality 65
14.6 Financial Obligations and Compensation 65
14.7 Informed Consent Processes 66
15.0 References 67
16.0 Appendices 72
16.1 APPENDIX A – Toxicity Grading 72
16.2 APPENDIX B – Response Criteria and Grading 73
16.2.1 Response Assessment in Neuro-Oncology (RANO) criteria (cited below). 73
16.2.2 Retrospective Research Analysis - Mathematical Models of Tumor Invasion and Proliferation 73
16.3 APPENDIX C: Cytokine Release Syndrome (CRS) in the Context of Cellular Immunotherapy (Clinical Symptoms & Revised Grading System) 74
16.3.1 Clinical signs and symptoms associated with CRS 74
16.3.2 Revised CRS Grading System 74
16.3.3 High-dose vasopressors (all doses are required for > 3 hours) 75
16.4 APPENDIX D – Case Report Forms 76

Abbreviations

Abbreviation	Meaning
AA	Anaplastic Astrocytoma
ACIT	Adoptive Cellular Immunotherapy
ADCC	Antibody-dependent Cell-mediated Cytotoxicity
ACT	Adoptive T Cell Therapy
AE	Adverse Event
AED	Antiepileptic Drug
ALT	Alanine Aminotransferase
ANC	Absolute Neutrophil Count
AST	Aspartate Aminotransferase
BM	Bone Marrow
CAR	Chimeric Antigen Receptor
CATD	Center for Applied Technology Development
CBG	Center for Biomedicine and Genetics
CD19t	Truncated Human CD19
CED	Convection Enhanced Delivery
CFR	Code of Federal Regulations
CofA	Certificate of Analysis
COH	City of Hope
CR	Complete Response
CRA	Clinical Research Associate
CRF	Case Report Form
CRP	C-Reactive Protein
CTCAE	Common Terminology Criteria for Adverse Events
CTEP	Cancer Therapy Evaluation Program
CTL	Cytolytic T Lymphocyte
DAC	Donor Apheresis Center
DLT	Dose Limiting Toxicity

DSMC	Data Safety Monitoring Committee
eCRFs	Electronic Case Report Forms
FcRs	Fc Receptors
FDA	Food and Drug Administration
GBM	Glioblastoma Multiforme
GCP	Good Clinical Practice
GMP	Good Manufacturing Practices
HACA	Human anti-chimeric antibodies
HAMA	Human anti-mouse antibodies
HSA	Human Serum Albumin
IB	Investigator Brochure
IC	Intracranial
ICF	Informed Consent Form
ICV	Intracranial ventricular
IDS	Investigational Drug Services
IL13Rα2	IL-13 receptor alpha 2
IND	Investigational New Drug
IRB	Institutional Review Board
LN	Lymph Node
LTR	Long-Terminal Repeat
MFD	Maximum Feasible Dose
MG	Malignant Glioma
MRI	Magnetic Resonance Imaging
MRS	Magnetic Resonance Spectroscopy
MTD	Maximum Tolerated Dose
NCI	National Cancer Institute
NIH OBA RAC	National Institute of Health Office of Biotechnology Activities, Recombinant DNA Advisory Committee
NSG	NOD scid gamma mice , (*NOD.Cg-Prkdc*^{scid} *Il2rg*^{tm1Wjl}*/SzJ*)
OIDRA	Office of IND Development and Regulatory Affairs

PBMC	Peripheral Blood Mononuclear Cell
PBT	Primary Brain Tumor
PD	Progressive Disease
PFNS	Preservative-free Normal-saline
PFS	Progression Free Survival
PI	Principal Investigator
PMT	Protocol Monitoring Team
PR	Partial Response
QA	Quality Assurance
Q-PCR	Quantitative Polymerase Chain Reaction
QS	Quality Systems
rhuIL-2	Recombinant Human Interleukin-2
RP2D	Recommended Phase 2 Dose
SAE	Serious Adverse Event
SD	Stable Disease
SEER	Surveillance, Epidemiology and End Results Database
SIN	Self-Inactivating
SSE	Single Subject Exemption
T_{CM}	Central Memory T cells
TCTRL	T Cell Therapeutics Research Laboratory
T_E	Cytolytic Effector Memory T cells
T_M	Memory T cells
T_N	Naïve T cells
$T_{N/MEM}$	Naïve and Memory T Cells
T_{Reg}	Regulatory T cells
TS	Tumor Sphere
UP	Unanticipated Problem
WBC	White Blood Cell
WPRE	Woodchuck Hepatitis Virus Post-Transcriptional Regulatory Element

1.0 Goals and Objectives (Scientific Aims)

1.1 Objectives:

1.1.1 *Primary Objectives*:

- Assess the feasibility and safety of cellular immunotherapy utilizing *ex vivo* expanded autologous memory-enriched T cells that are genetically modified using a self-inactivating (SIN) lentiviral vector to express a IL13Rα2-specific, hinge-optimized, 41BB-costimulatory CAR, as well as a truncated human CD19 for participants with recurrent/refractory malignant glioma in one of the following ways: Arm 1 (intratumoral delivery of IL13(EQ)BBζ/CD19t+ T_{CM}), Arm 2 (intracavitary delivery of IL13(EQ)BBζ/CD19t+ T_{CM}), Arm 3 (intraventricular delivery of IL13(EQ)BBζ/CD19t+ T_{CM}), Arm 4 (dual delivery [both intratumoral and intraventricular] of IL13(EQ)BBζ/CD19t+ T_{CM}), or Arm 5 (dual delivery of IL13(EQ)BBζ/CD19t+ $T_{N/MEM}$); and
- Determine maximum tolerated dose schedule (MTD)/maximum feasible dose schedule (MFD) and a recommend phase II dosing plan (RP2D) for each arm based on dose limiting toxicities (DLTs) and the full toxicity profile.

1.1.2 *Secondary Objective*:

- In research participants who receive the full schedule of three CAR+ T cell doses:
  - Estimate disease response rates,
  - Estimate median overall survival, and
  - Estimate the mean change from baseline in quality of life using the EORTC QLQ-C30 during and post treatment;
- Describe cytokine levels (tumor cavity fluid, CSF, peripheral blood) over the study period;
- Describe CAR T cell and endogenous immune populations (CSF, tumor cavity fluid, peripheral blood) over the study period; and
- Identify tumor and tumor micro-environment markers associated with response to CAR T cells.

1.1.3 *Exploratory Objectives*

- Assess the timing and extent of brain inflammation following CAR T cell administration;
- Evaluate CAR T cell product characteristics; and
- For research participants who undergo a second resection or autopsy:
  - Evaluate CAR T cell persistence in the tumor micro-environment and the location of the CAR T cells with respect to the injection, and
  - Evaluate IL13Rα2 antigen expression levels pre and post CAR T cell therapy.

2.0 Background

2.1 Introduction/Rationale for Development

2.1.1 Glioblastoma Multiforme

Glioblastoma multiforme (GBM), a malignant form of astrocytoma, is the most common primary intracranial neoplasm. The incidence of GBM increases steadily above 45 years of age with a prevalence of approximately 7500 cases in the USA. Despite numerous attempts to improve the outcome of patients with GBM, the 3-year survival of these patients is only 6% with median survival of 14.6 months (SEERs database).

GBM is a diffusely infiltrating tumor that spreads microscopically throughout the brain. Therefore, all local therapies, such as surgery and radiation, are inherently palliative. Long-term disease control requires therapies targeting tumor cells throughout the brain, such as chemotherapy, small molecule inhibitors or cellular immunotherapy.

2.1.2 Treatment Options for GBM

Patients with newly diagnosed GBM who are treated with maximal surgical resection, followed by radiotherapy and Temozolomide, and then maintenance Temozolomide for 6 months, have a median survival of 14.6 months.[9]

For recurrent GBM the prognosis is equally poor with a median survival of 3 to 9 months.[10] Gliadel wafers and Avastin have been approved for subjects with recurrent GBM, but neither of these therapies appear curative. Several clinical trials have tested the efficacy of inhibitors of EGFR, VEGFR TK, PDGFR, or mTOR for recurrent malignant glioma (MG) - but despite initial enthusiasm, treatment with these single pathway inhibitors has generally been disappointing, with response/survival rates not being superior to that of traditional chemotherapies.[10-19] In fact, analysis of pooled data from multiple phase II clinical trials for recurrent/progressive glioblastoma within the North American Brain Tumor Consortium was not encouraging. This study demonstrated an overall 6-month progression-free survival (PFS) rate of 7% for patients with recurrent/progressive glioblastoma treated with small molecule inhibitors as a single agent and represents an update to the study published in 1999.[10,20] Thus, due to the lack of salvage regimens with curative potential, patients with relapsed or refractory MG are prime candidates for referral to experimental studies such as the one proposed here.

2.1.3 IL13Rα2 expression and GBMs:

The 42-kDa monomeric high affinity IL-13 receptor, IL13Rα2, is an attractive target for brain tumor therapy. It has been shown to promote tumor migration and invasion[21], and protect tumor cells from apoptosis thereby contributing to tumor growth.[22] Furthermore, IL13Rα2 is expressed by a high percentage of high-grade gliomas (approximately 58% of GBM), and is a prognostic indicator of poor patient survival.[23] Importantly, IL13Rα2 is not expressed at significant levels on normal brain tissue.[24-27]

2.1.4 Adoptive Cellular Immunotherapy

One area of significant translational research in the cancer Adoptive Cellular Immunotherapy (ACIT) field is the genetic manipulation of T cells to endow them with tumor specificity and countermeasures against tumor-mediated immunosuppression.[28-37] Adoptive T cell therapy for melanoma is one example of the curative potential of cellular immunotherapy, even in settings of metastatic refractory disease.[38] However, there are still hurdles to be overcome – e.g., the major cause of treatment failure in ACIT is the inability of the transferred cells to engraft and persist.[36,39] This study intends to help address these issues.

2.1.4.1 Immunobiology of Memory Enriched T Cell Adoptive Transfer.

The *in vivo* persistence of cultured T cells is improved by depletion of host lymphocytes before cell transfer and by the administration of IL-2 after cell transfer; but in half of treated melanoma patients these

interventions still did not result in long-term persistence of transferred T cells or sustained tumor regression.[40,41] We hypothesize that intrinsic properties of T cells may be a critical determinant of their fate *in vivo* following adoptive transfer. The pool of peripheral blood lymphocytes from which T cell products are derived *ex vivo* consist of naïve T cells (T_N), and antigen experienced memory T cells (T_M), which include central memory T cells (T_{CM}). T_{CM} express CD62L and CCR7 which promote migration into lymph nodes, produce IL-2, and proliferate rapidly in response to antigen stimulation. In response to antigen *in vivo*, T_{CM} differentiate into cytolytic effector T (T_E) cells that express high levels of granzymes and perforin, but are otherwise short-lived.[42,43] Thus, differentiation to a terminal T_E phenotype during culture has been suggested to explain the poor survival of transferred T cells in clinical trials.[44,45]

In the normal host, T cell memory persists for life, suggesting that some T_M have the ability to self-renew or to revert to the memory pool after differentiating to T_E in response to antigen re-exposure.[46] Using an immunodeficient mouse model, as well as a non-human primate model (*Macaca nemestrina*) relevant for human translation, we have observed that T_E cells derived from sort-purified CD62L$^+$ T_{CM} persisted in the blood long-term after adoptive transfer, migrated to memory T cell niches in the lymph nodes (LN) and bone marrow (BM), reacquired phenotypic properties of T_M, and responded to antigen challenge *in vivo*.[7,8] Thus, we hypothesize that human T_{CM}-derived effectors will retain their programming for persistence after their genetic modification and *ex vivo* propagation, and, upon adoptive transfer to GBM patients shortly following tumor biopsy or resection, as applicable, and intracavitary catheter placement, will produce robust anti-tumor responses against IL13Rα2+ MG.

2.1.5 Immunotherapy of Glioblastoma with Gene Modified T Cells.

2.1.5.1 Redirected Specificity of T Cells Expressing Chimeric Antigen Receptors (CAR)

Chimeric antigen receptors (CAR) engineered to consist of an extracellular antigen-binding domain fused to the intracellular signaling domain of the T cell antigen receptor complex zeta chain have the ability, when expressed in T cells, to redirect antigen recognition and cytolytic activity.[1] The design of CAR with target specificities for tumor cell-surface epitopes is a conceptually attractive strategy to generate anti-tumor immune effector cells for adoptive therapy as it does not rely on pre-existing anti-tumor immunity. These receptors are "universal" in that they bind antigen in an MHC independent fashion; and, thus, one receptor construct can be used to treat a population of patients with antigen positive tumors. Several constructs for targeting human tumors have been described in the literature including receptors with specificities for CD19, Her2/Neu, CEA, ERB-B2 and CD44v6.[47] These epitopes all share the common characteristic of being cell-surface moieties accessible to binding by the CAR. *In vitro* studies have demonstrated that both CD4$^+$ and CD8$^+$ T cell effector functions can be triggered via these receptors to specifically lyse tumor targets and secrete an array of pro-inflammatory cytokines including IL-2, TNF, IFN-γ, and GM-CSF. Moreover, animal models have demonstrated the capacity of adoptively transferred CAR-expressing T cells to eradicate established tumors.

2.1.5.1 IL13Rα2-specific CAR

Based on the IL13Rα2 expression profile described in **Section 2.1.3**, we have been interested in genetically modifying T cells to express an IL13Rα2-specific CAR. The proposed IL13Rα2-specific CAR (IL13(EQ)BBζ), consisting of a membrane-tethered human IL-13 ligand mutated at a single site (E13Y) to reduce potential binding to IL13Rα1,[5] has the ability, when expressed in T cells, to redirect antigen recognition and targeting against IL13Rα2-expressing MG cells, including glioma cells with stem-like characteristics.[5,6] This IL13(E13Y) tumor targeting domain linked to a human CD3ζ signaling domain has also been previously evaluated in our recent glioma trials (COH Protocol Nos. 01020 and 07082). The similarities of the CAR used in these past MG studies versus the current proposed clinical study is depicted in **Figure 2.1**. Indeed, prior clinical experience - by our group using CAR T cells (reference **Section 2.4.1** below), and others using an IL13-immunotoxin delivered intratumorally [48] - provide evidence that immunotherapeutic approaches targeting IL13Rα2 for high-grade glioma can be safe and well-tolerated in patients.

Figure 2.1. Characterization of past and current IL13Rα2-specific CAR. The IL13(E13Y)-zetakine CAR used in IRBs 01020 and 07082 (Left) is composed of the IL13Rα2-specific human IL-13 mutein (huIL-13(E13Y)), human IgG4 Fc spacer (huγ4Fc), human CD4 transmembrane (huCD4 tm), and human CD3ζ chain cytoplasmic (huCD3ζ cyt) portions as indicated. The IL13(EQ)BBζ CAR of this study (Right) is the same as the IL13(E13Y)-zetakine with the exception of the two point mutations, L235E and N297Q indicated in red, that are located in the CH2 domain of the IgG4 spacer, and the addition of a costimulatory 4-1BB cytoplasmic domain (4-1BB cyt).

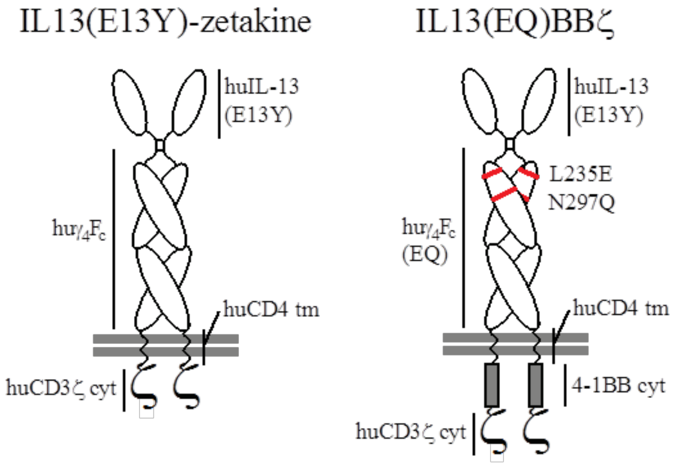

2.1.5.2 Hinge-optimized CAR

This protocol will use an IL13Rα2-specific CAR that has been modified at two sites within the CH2 region (L235E; N297Q) which have been known to be important for recognition and binding by Fc receptors (FcRs) (reviewed in Current Opinion Biotechnology, 2009[49]). FcRs on NK cells and macrophages are known to mediate various immune functions such as antibody-dependent cell-mediated cytotoxicity (ADCC) and phagocytosis. Our preclinical findings suggest that, while the L235E/N297Q mutations do not alter the antigen-specific functionality of the CAR, they do decrease the binding of recombinant soluble FcR molecules, and improve the engraftment and therapeutic efficacy of CAR-expressing cells in *in vivo* mouse models.

2.1.5.3 Second Generation Costimulatory CAR.

Our work and others have established that CAR containing the costimulatory signaling domain of CD137 (4-1BB) and/or CD28 in tandem with the cytolytic activating domain of CD3ζ exhibit enhanced proliferation and anti-tumor function as compared to CARs containing CD3ζ alone.[1,50,51] For this protocol we will incorporate the human 4-1BB costimulatory signaling domain in series with human CD3ζ as part of our CAR design since this costimulatory domain meets all the criteria for providing costimulation, including improvement of the survival and maintenance of memory T cells.[52,53] Such costimulatory CAR have been proven to be both safe and highly effective in the clinic.[54-56]

2.1.5.4 CD19t as a tracking marker

Finally, the proposed T cell product will also be modified to express a truncated human CD19 (CD19t), which lacks the cytoplasmic signaling tail (truncated at amino acid 323). Co-expression of CD19t provides an inert, non-immunogenic surface marker that will allow for accurate measurement of gene modified cells, as well as efficient cell tracking of the therapeutic T cells *in vivo* following adoptive transfer.

2.1.5.5 Use of 'bulk' T_{CM}

The rationale for using T_{CM} for genetic modification is based on studies described in **Section 2.1.4.1**, where, using both murine xenograft models and a non-human primate model relevant for human translation, it was found that effector T cells derived from sort-purified $CD62L^+$ T_{CM} persisted after adoptive transfer and responded to antigen challenge *in vivo.*[7,8]

Our use of a "bulk" T_{CM} population that includes both CD4+ and CD8+ cells for gene modification is based on ACIT studies in animal models and in the clinic that have documented the importance of including $CD4^+$ T helper cells for optimal $CD8^+$ T cell effector function, as well as persistence.[57-59] Furthermore, it has now become a standard in the field to include both $CD4^+$ and $CD8^+$ T cells in immunotherapies that use CAR-modified T lymphocytes for the treatment of malignancy (reviewed in Molecular Therapy 2011[60]). However, to reduce the presence of $CD4^+$ regulatory T cells (Tregs) that might inhibit the anti-tumor efficacy of the cell product,[61] it is important to note that, instead of a CD4-depletion, we are including a CD25-depletion step (i.e., since these Tregs are known to express high levels of CD25/IL-2 receptor). The feasibility of depleting functional $CD25^+$ Tregs in large scale under clinical-grade conditions for use in ACIT has also been previously reported.[62]

2.1.5.6 Use of 'bulk' $T_{N/MEM}$

As a separate arm for comparison to the use of T_{CM} as a starting T cell population, this study will use enriched CD62L+ naïve and memory T cells ($T_{N/MEM}$) for CAR engineering as an investigational cell product. $T_{N/MEM}$ cells include both the central memory (T_{CM}) and stem cell memory (T_{SCM}) populations along with naïve T cells (T_N). The enrichment methods for the two CD62L+ T cell starting populations differ only in the inclusion (T_{CM}) or elimination ($T_{N/MEM}$) of the CD45RA-depletion step. Specifically, $T_{N/MEM}$ cells are produced by CliniMACS/AutoMACS™ depletion of the CD14+ monocytes and the CD25+ regulatory T cells, followed by AutoMACS™ selection of the CD62L+ memory population. This is in contrast to T_{CM} cells which are produced by CliniMACS/AutoMACS™ depletion of the CD14+ monocytes, the CD25+ regulatory T cells, and the CD45RA+ T_N and T_{SCM}, followed by AutoMACS™ selection of the CD62L+ population. This use of $T_{N/MEM}$-derived T cell products will increase the feasibility of our manufacturing platform as well as the potential efficacy of the clinically administered cell products. Indeed, elimination of the CD45RA-depletion step for $T_{N/MEM}$ enrichment allows for the recovery of more T cells as compared to the T_{CM} enrichment platform, thus allowing for an increased number of T cells that can be used as a starting population for genetic engineering. This will increase the feasibility of manufacturing CAR T cell products for clinical use, especially with research participants whose CD3+ T cell count is already low due to prior therapy. Furthermore, our pre-clinical in vivo studies suggest that $T_{N/MEM}$-derived cell products mediate anti-tumor responses that are superior to those seen with T_{CM}-derived cell products. This observation is supported by the literature (reviewed in[63]) demonstrating that adoptive cell transfer of less differentiated T cell subsets (including the T_N and T_{SCM} found in the $T_{N/MEM}$ population) consistently exhibit superior in vivo anti-tumor activities compared to more differentiated effector T cells.

2.1.6 Route of Administration and Dosing.

For research participants who receive either intratumoral or intracavitary delivery of CAR T cells: CAR T cells will be administered manually followed by up to 0.5 mL preservative-free normal-saline (PFNS) over approximately 5 minutes through a Rickham catheter.

For research participants who receive intraventricular delivery of CAR T cells: CAR T cells will be administered manually followed by up to 0.5 mL preservative-free normal-saline (PFNS) over approximately 5 minutes through a Rickham catheter, placed into the lateral ventricle.

For research participants who receive dual delivery (both the intratumoral/intracavitary and intraventricular delivery) of CAR T cells: the listed CAR+ T cell doses will be delivered into both the tumor bed/cavity and the ventricle, thereby doubling the total infused dose. For example, in Dose Schedule 2 Cycle 1: $10x10^6$ CAR+ T cells will be delivered intratumorally/intracavitary and $10x10^6$ CAR+ T cells will be delivered intraventricularly for a total Cycle 1 infused dose of $20x10^6$ CAR+ T cells. The administration of each dose will follow the rules listed above.

The PFNS flush is meant to clear the administration line and 'push' remaining CAR T cells through the catheter.

Successful manual administration through a Rickham catheter of similar gene-modified IL13Rα2-specific cellular products has been successful in COH IRB Protocol Nos. 01020 (BB-IND 10109) and 07082 (BB-IND 14194).

As of 03/02/2018, we have closed Arm 1 (biopsy with an N=2) as biopsy participants can be included in the ICV or dual arms. The study will continue to enroll participants in the remaining 4 arms, intracavitary (Arm 2), intraventricular (Arm 3), or dual delivery (both intratumoral and intraventricular) (Arms 4 and 5).

Note: If a research participant progresses on either Arm 1 or 2 after the first three CAR T cell infusion cycles, at the discretion of the PI, the research participant may move to intraventricular administration of CAR T cells for the optional infusion cycles.

2.2 Overview of Proposed Study

This Phase I, open-label, nonrandomized study proposes to examine the feasibility and safety of intratumoral/intracavitary/intraventricular/dual delivery administration of autologous *ex vivo* expanded central memory-enriched T cells (T_{CM} or $T_{N/MEM}$) that are genetically modified using a self-inactivating (SIN) lentiviral vector to express an IL13Rα2-specific, hinge-optimized, 41BB-costimulatory chimeric antigen receptor (CAR) as well as a truncated human CD19 (IL13(EQ)BBζ/CD19t+ T cells) to adult research participants with recurrent or refractory malignant glioma (MG).

In this protocol, research subjects with documented IL13Rα2-positive recurrent/refractory high-grade glioma (Grades III and IV) will be enrolled and undergo PBMC collection. Research participant T_{CM} or $T_{N/MEM}$ will be enriched from peripheral blood mononuclear cells (PBMC) using the CliniMACS® device to immunomagnetically select for $CD45RO^+/CD62L^+$ T_{CM} or $CD62L^+$ $T_{N/MEM}$. These cells will then be activated with anti-CD3/CD28 Dynal beads, transduced with a SIN lentiviral vector that directs the expression of an IL13Rα2-specific, hinge-optimized, 41BB-costimulatory CAR (IL13(EQ)BBζ) as well as a truncated human CD19 (CD19t), a non-immunogenic surface marker for both *in vivo* detection and potential *ex vivo* selection. The activated/genetically modified IL13(EQ)BBζ/CD19t+ T cells will be expanded *in vitro* with IL-2/IL-15 and then cryopreserved. Concurrent with the manufacturing process, research participants will undergo biopsy or resection of their tumor(s), as applicable, followed by placement of a Rickham catheter(s) and baseline imaging. Following recovery from surgery, research participants will then receive an initial low dose (cycle 1) followed by 2 additional infusions at the 5-fold higher cell dose (cycles 2 and 3) of autologous IL13(EQ)BBζ/CD19t+ T cells, potentially followed by additional cycles at up to the highest tolerated cell dose (cycles 4+). Depending on the participant's disease status, CAR T cells will be administered one of four ways: (1) directly into the tumor (Arm 1), (2) into the tumor resection cavity (Arm 2), (3) into the lateral ventricles (intraventricularly; Arm 3) or (4) into either the tumor bed/cavity (intratumoral) and into the lateral ventricles (intraventricular) (Arms 4 and 5 = dual delivery).

The primary objectives of the study are to assess the feasibility and safety of intratumoral (Arm 1), intracavitary (Arm 2), intraventricular (Arm 3), or dual delivery (Arm 4) cellular immunotherapy utilizing *ex vivo* expanded autologous IL13(EQ)BBζ/CD19t+ T cells for research participants with recurrent/refractory malignant glioma, and to determine the maximum tolerated dose schedule (MTD)/maximum feasible dose scheduled (MFD) and a recommend phase II dosing plan (RP2D) for each arm based on dose limiting toxicities (DLTs) as defined in **Section 5.7**. The primary endpoints are grade 3 toxicities, DLTs, and all other toxicities.

This study will be conducted in compliance with the protocol, Good Clinical Practice (GCP) and the applicable regulatory requirements.

2.3 Preclinical Studies

2.3.1 IL13(EQ)BBζ/CD19t^{+} TCM.

The methods for the *in vitro* enrichment of human TCM, their genetic modification and expansion to generate clinical cell products that express CAR have been developed in our lab using blood obtained from human donors.[64] The feasibility of engineering the expression of both a costimulatory CAR and a truncated selection marker has also been published.[65] Furthermore, the 13 autologous TCM clinical products made to date under COH IRB Protocol Nos. 09174 (BB-IND 14645) and 12224 (BB-IND 15490) also support our experience in manufacturing clinical products similar to that described in this protocol.

In addition to this experience, we have also performed two small scale qualification runs (detailed below) on cells procured from healthy donors using the manufacturing platform described above in **Section 2.2**, as well as 3 clinical scale GMP qualification runs as described in BB-IND 16226. Importantly, these qualification runs expanded >80 fold within 28 days (**Table 2.1**) and the expanded cells expressed the IL13(EQ)BBζ/CD19t transgenes (**Table 2.1** and **Figure 2.2**), and exhibited IL13Rα2-specific effector function (**Figure 2.3**).

Table 2.1. Summary of CAR expression and fold-expansion

Cell Product	CAR	CD19	CD4*	CD8*	Fold-Expansion
HD006.5	20%	22%	24%	76%	84-fold; 28 days
HD187.1	18%	25%	37%	63%	259-fold 28 days

*Percentages of CD4 and CD8 are donor specific and within the normal expected range

Figure 2.2. Flow cytometric analysis of surface transgene and T cell marker expression. IL13(EQ)BBζ/CD19t+ TCM HD006.5 and HD187.1 were co-stained with anti-IL13-PE and anti-CD8-FITC to detect CD8+ CAR+ and CD4+ (i.e., CD8 negative) CAR+ cells (**A**), or anti-CD19-PE and anti-CD4-FITC to detect CD4+ CD19t+ and CD8+ (i.e., CD4 negative) CAR+ cells (**B**). **C**, IL13(EQ)BBZ/CD19t+ TCM HD006.5 and HD187.1 stained with fluorochrome-conjugated anti-CD3, TCR, CD4, CD8, CD62L and CD28 (grey histograms) or isotype controls (black histograms). **A**-**C**, Percentages based on viable lymphocytes (DAPI negative) stained above isotype.

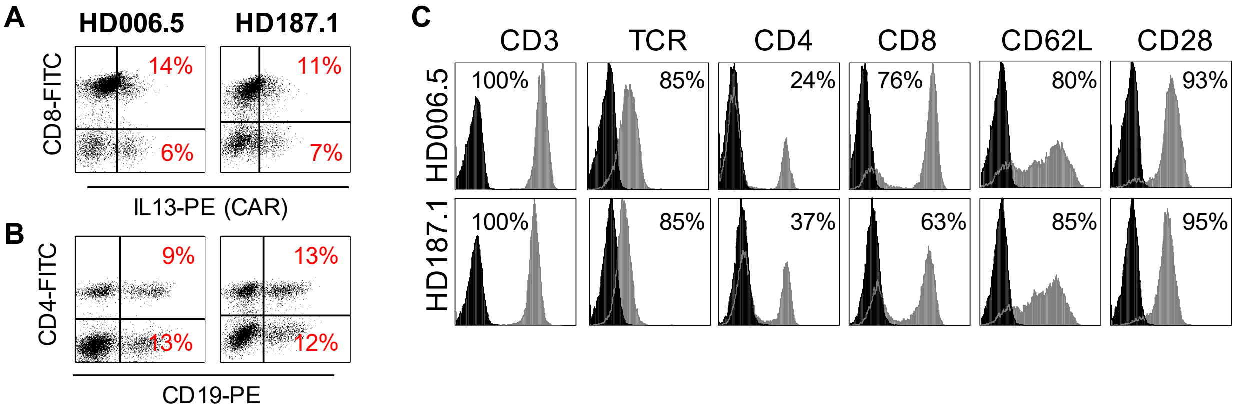

Figure 2.3. Effector activity of IL13(EQ)BBζ/CD19t+ T_{CM}. A, IL13(EQ)BBζ/CD19t+ T_{CM} HD006.5 and HD187.1 were used as effectors in a 6-hour ^{51}Cr-release assay using a 10E:1T ratio based on CD19t expression. The IL13Rα2-positive tumor targets were K562 engineered to express IL13Rα2 (K562-IL13Rα2) and primary glioma line PBT030-2, and the IL13Rα2-negative tumor target control was the K562 parental line. **B**, IL13(EQ)BBζ/CD19t+ T_{CM} HD006.5 and HD187.1 were evaluated for antigen-dependent cytokine production following overnight co-culture at a 10E:1T ratio with the same IL13Rα2-positive and negative targets as described in (A). Cytokine levels were measured using the Bio-Plex Pro Human Cytokine TH1/TH2 Assay kit and INF-γ levels are depicted.

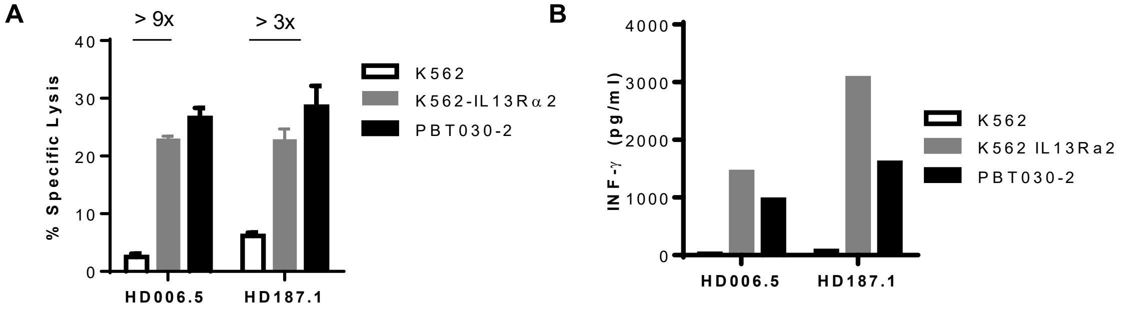

The efficacy of the proposed strategy is further supported by our preclinical studies demonstrating that IL13(EQ)BBζ/CD19t+ T_{CM} exhibit anti-tumor efficacy in *in vivo* mouse models. Specifically, we have evaluated the anti-tumor potency of IL13(EQ)BBζ/CD19t+ T_{CM} against the IL13Rα2+ primary low-passage glioblastoma tumor sphere line PBT030-2, which has been engineered to express both EGFP and firefly luciferase (ffLuc) reporter genes (PBT030-2 EGFP:ffLuc).[6] Our group has generated a panel of primary lines (PBT) from patient glioblastoma specimens grown as tumor spheres (TSs) in serum-free media.[6,66] These expanded TS lines exhibit stem cell-like characteristics, including expression of stem cell markers, multi-lineage differentiation and capacity to initiate orthotopic tumors in immunocompromised mice (NSG) at low cell numbers.[6,66] The PBT030-2 EGFP:ffLuc TS-initiated xenograft model ($0.1x10^6$ cells; 5 day engraftment) has been previously used to evaluate *in vivo* anti-tumor activity in NSG mice of IL13Rα2-specific CAR expressing T cells manufactured under our glioma trial (COH Protocol No. 01020),[6] whereby three injections of $2x10^6$ cytolytic T lymphocytes (CTLs) over a course of 2 weeks were shown to reduce tumor growth. However, in these experiments the majority of the PBT030-2 tumors eventually recurred. By comparison, a single injection of IL13(EQ)BBζ/CD19t+ T_{CM} ($1.1x10^6$ CAR+ T_{CM}; $2x10^6$ total T_{CM}) exhibited robust anti-tumor activity against PBT030-2 EGFP:ffLuc TS-initiated tumors ($0.1x10^6$ cells; 5 day engraftment) (reference **Figure 2.4**). As compared to NSG mice treated with either PBS or mock transduced T_{CM} (no CAR), IL13(EQ)BBζ/CD19t+ T_{CM} significantly reduce ffLuc flux ($p < 0.001$ at $\geq$18-days) and significantly improve survival ($p = 0.0008$).

Figure 2.4. Regression of established glioma tumor xenografts after adoptive transfer of IL13(EQ)BBζ/CD19t+ T_{CM}. EGFP-ffLuc^{+} PBT030-2 tumor cells (1×10^{5}) were stereotactically implanted into the right forebrain of NSG mice. On day 5, mice received either $2x10^{6}$ IL13(EQ)BBζ/CD19t+ T_{CM} ($1.1x10^{6}$ CAR+; n=6), $2x10^{6}$ mock T_{CM} (no CAR; n=6) or PBS (n=6). **A**, Representative mice from each group showing relative tumor burden using Xenogen Living Image. **B**, Quantification of ffLuc flux (photons/sec) shows that IL13(EQ)BBζ/CD19t+ T_{CM} induce tumor regression as compared to mock-transduced T_{CM} and PBS ($^{\#}p<0.02$, $^{*}p<0.001$, repeated measures ANOVA). **C**, Kaplan Meier survival curve (n=6 per group) demonstrating significantly improved survival (p=0.0008; log-rank test) for mice treated with IL13(EQ)BBζ/CD19t+ T_{CM}.

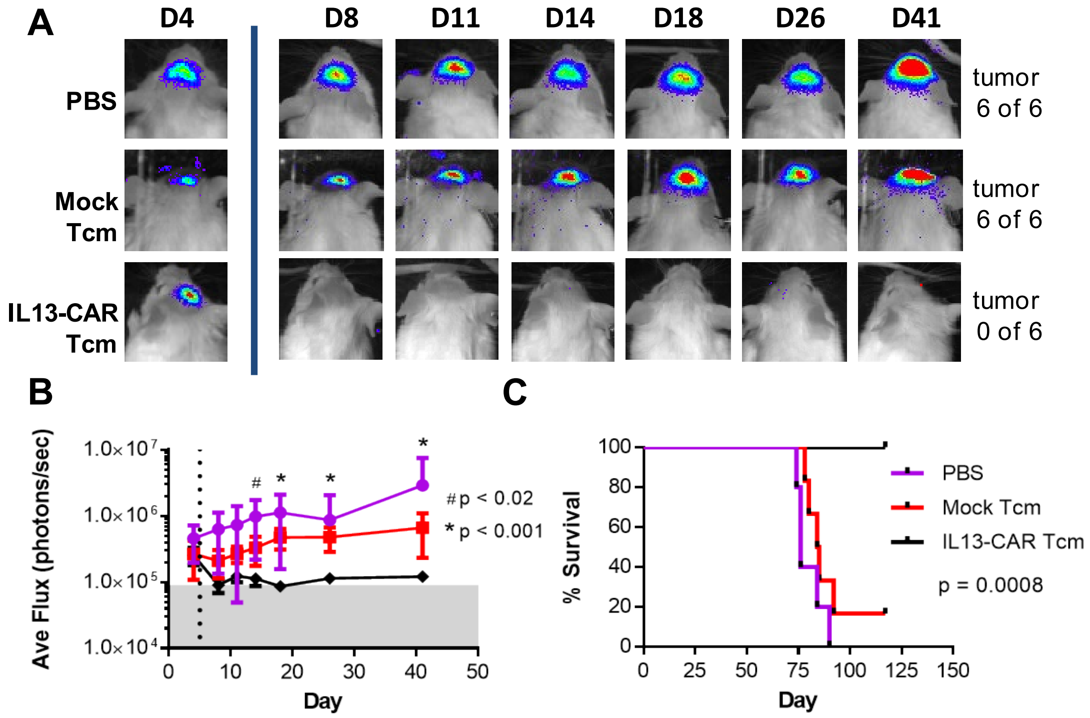

2.4 Human Studies

2.4.1 Clinical ACIT Experience at COH Using Genetically Modified T cells (CAR T cells).

We have significant experience (over 10 years) with clinical adoptive cellular immunotherapy at COH using genetically-modified T cells.[4,67,68] Our studies to date have demonstrated the tolerability of cell doses and lack of lymphoproliferative sequelae based on *in vivo* proliferation of transferred cells. Our lymphoma trials (COH IRB Protocol Nos. 09174 and 12224) demonstrate the feasibility for manufacturing gene-modified CAR+ T_{CM}. Perhaps most relevant to this application, however, are the gene therapy trials for malignant glioma (MG) (COH Protocol Nos. 01020 and 07082) evaluating the safety in targeting IL13Rα2 with repetitive intratumoral/intracavitary doses of CAR-expressing T cells. Reference **Table 2.2** for a summary of Safety and Tolerability of these MG trials.

Table 2.2. Safety and Tolerability of previous MG trials at COH

IND Trial	Patient	T-cell Doses	Infusion Schedule	Maximum Tolerated Dose	Cumulative T-cell Dose	Adverse Event [a] (Time of Occurrence)[b]	Survival Post Relapse (months) [c]
**10109** (IRB 01020)	UPN028	11	4 weekly cycles; 3 x per week (2 weeks on, 1 week rest, 2 weeks on)	10^8	9.6×10^8	Headache (day of 1st infusion; day after final infusion)	10.3
	UPN031	12		10^8	10.6×10^8	Neurologic – Other (day after final infusion)	8.6
	UPN033	12		10^8	10.6×10^8	None	13.9
**10109** (IRB 06164)	UPN033	5	Consecutive days	10^8	3.75×10^8	Low WBC (day of infusion 2)<br>Headache (days of infusions 2-5)<br>Fatigue (day of infusion 5)	11.8[d]
**14149** (IRB 07082)	UPN100	4	2 weekly cycles; 2 x per week	10^8	4×10^8	Dehydration (day after final infusion)<br>Stroke (two weeks after final infusion)	3.6[e]
	UPN102	4		10^8	4×10^8	None	3.5[e]
	UPN103	4		10^8	4×10^8	None	7.5
	UPN104	4		10^8	4×10^8	Gait disturbance (2nd day of first week)	2.8
	UPN105	4		10^8	4×10^8	None	11.9
	UPN106	4		10^8	4×10^8	None	3.1

[a] Only events of Grade 3 or higher, according to NCI Common Toxicity Criteria, with possible attribution to the T cell administration are reported.
[b] Occurrence reported as related to T cell infusion schedule.
[c] Relapse prior to T cell therapy was used to calculate survival.
[d] Calculated after biopsy/relapse diagnosed on day he was placed on compassionate use protocol.
[e] Calculation based on estimated date (only month/year recorded) of diagnosed progression after standard therapy.

2.4.1.1 COH Experience with Zetakine Redirected CTLs for Glioma Immunotherapy

COH has completed 2 clinical trials utilizing zetakine redirected CTLs for glioma immunotherapy. The first study, COH Protocol No. 01020 (BB-IND 10109) opened in 2002. The trial was designed as an initial pilot study to evaluate the feasibility and safety of delivering an autologous cloned $CD8^+$ CTL genetically modified to co-express the IL13-zetakine and the bi-functional selection-suicide gene HyTK. Post re-resection intracavitary cell doses were stratified based on intra-research participant dose escalation starting at 10^7 to 10^8 total cells over the first 3 infusions followed by 9 additional cell doses of 10^8 over 4-weeks. This trial enrolled research participants following initial therapy where cell products were produced and cryopreserved until tumor relapse necessitated re-resection. This prospective cell product manufacturing strategy was employed based on a consensus view that the 10-12 week production time was too long for enrollment at the time of relapse given the typically rapid demise of these research participants facing recurrent/progressive disease. Cell products meeting all FDA-mandated release criteria were successfully manufactured for research participants enrolled on to the protocol, however, only three research participants met treatment eligibility and subsequently underwent adoptive therapy. A case study on one of the research participants has been reported with respect to the non-invasive detection of the autologous IL13(E13Y)-$zetakine^+$/$HyTK^+$ $CD8^+$ CTL using ^{18}F-FHGB PET.[4] Overall, there were 3 total SAEs of grade three or higher that were possibly related to the infusion of the CAR T cells – one research participant had a headache at the start of her third cycle of infusions, and then again the day after her final T cell infusion; and a second research participant experienced a transient neurologic event that caused a shuffling gait and tongue deviation to the left one day following her final T cell infusion. Together, the three patients under COH Protocol No. 01020 had a mean survival of 11 months after relapse, with best survival of almost 14 months. One research participant (UPN031) had evidence that their IL13Rα2-specific adoptively transferred T cells were able to eliminate antigen positive glioma cells (manuscript in preparation). Another one of the three research participants under COH Protocol No. 01020 relapsed (on the hemisphere opposite that of the T cell infusion site) 20 days after his last T cell infusion, and was placed on a compassionate use COH Protocol No. 06164, also filed under BB-IND 10109, where he received 5 daily intratumoral infusions of the previously manufactured CAR T cells, at doses that escalated from $2.5x10^7$ to $1x10^8$. This participant experienced 6xSAEs of grade three or higher that were possibly related to the infusion of CAR T cells – four of them were headaches each day he received the second through fifth T cell infusions; one was a low white blood cell count on the day of the second T cell infusion; and another was fatigue experienced on the final day of T cell infusion. Importantly, this patient exhibited a regression of the recurrent IL13Rα2+ GBM lesion following the intratumoral delivery of the CTL, as detected by MRI (**Figure 2.5**).

Figure 2.5. Regression of recurrent IL13Rα2+ glioma. Post gadolinium MRI (Left images) and MRS analysis of necrotic volume (Right bar graph) before and after the 5 daily infusions of autologous IL13(E13Y)-zetakine+/HyTK+ CD8+ CTL under the compassionate use protocol. Lack of tumor recurrence at the original treatment site (right occipital) and increase in necrosis at the recurrent site that was re-treated with T-cells (left corpus callosum, red circles) are highly suggestive of therapeutic efficacy.

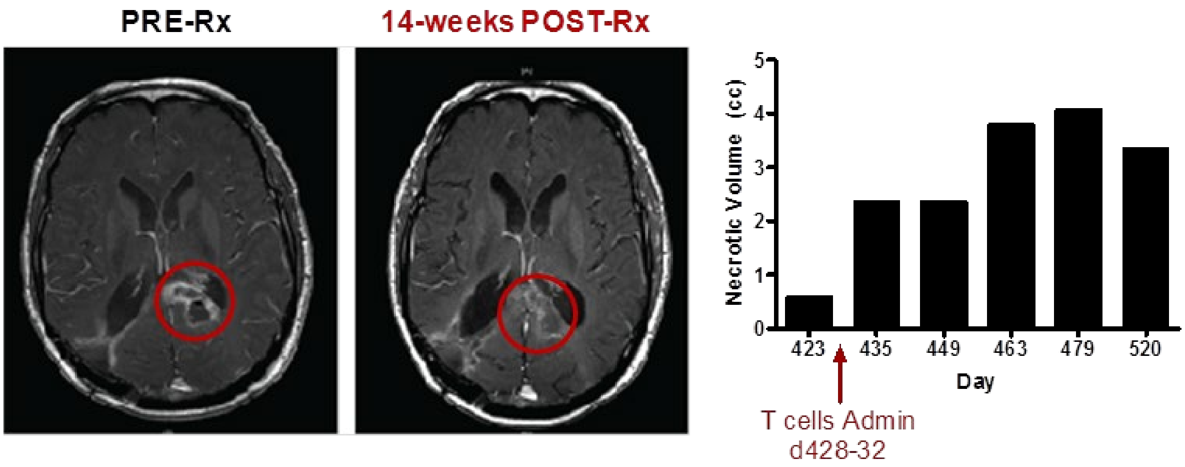

The second study, COH Protocol No. 07082 (BB-IND 14194) opened in 2010. The trial was designed as a phase I study to evaluate the safety of an allogeneic CD8+ CTL line genetically modified to express IL13(E13Y)-zetakine and HyTK, and to be resistant to glucocorticoids, administered intratumorally in conjunction with convection enhanced delivery (CED) of recombinant human Interleukin-2 (rhuIL-2), for research participants with recurrent or refractory/progressive malignant glioma (WHO Grades 3 or 4). In this study six (6) research participants received 2 cycles of 2x CAR T cell infusions per week (total of 4 infusions) at cell doses of approximately 10^8 cells. There were a total of 3xgrade three SAEs in 2 patients that were possibly related to the infusion of the CAR T cells – the first was dehydration one day after the 2 week treatment cycle; the second was a stroke that occurred in this same participant nearly two weeks after the final T cells/IL-2 infusion and was distant from the injection site; and the third event was a gait disturbance which occurred in another research participant on the second day of his first week of treatment, but this did not delay his next infusion, and his treatment course was otherwise uncomplicated. Overall, the therapy was well tolerated with a mean survival of 5 months after relapse, and best survival of almost 12 months in one patient. Furthermore, four research participants had evidence of tumor necrosis at the site of CAR T cell administration.

Taken together, our prior clinical experience treating a total of 9 patients demonstrates the safety of administering intratumoral/intracavitary IL13Rα2-specific CAR T cells and the potential efficacy of this therapeutic approach.

3.0 Patient Eligibility

3.1 Screening Inclusion Criteria

3.1.1 Disease Status

- Participant has a prior histologically-confirmed diagnosis of a grade III or IV glioma, or has a prior histologically-confirmed diagnosis of a grade II glioma and now has radiographic progression consistent with a grade III or IV MG after completing standard therapy.
- Radiographic evidence of progression/recurrence of the measurable disease more than 12 weeks after the end of the initial radiation therapy.

3.1.2 Age Criteria, Performance Status and Life Expectancy

- Male or female research participants between 12-75 years of age
- KPS ≥60%
- Life expectancy > 4 weeks

3.1.3 Child Bearing Potential

The effects of IL13(EQ)BBζ/CD19t+ T cells on the developing fetus are unknown. For this reason, women of child-bearing potential and men must agree to use adequate contraception (hormonal or barrier method of birth control or abstinence) prior to study entry and for six months following duration of study participation. Should a woman become pregnant or suspect that she is pregnant while participating on the trial, she should inform her treating physician immediately.

3.1.4 Protocol-Specific Criteria

- COH Clinical Pathology confirms IL13Rα2+ tumor expression by immunohistochemistry (≥ 20%, 1+).
- All research participants must have the ability to understand and the willingness to sign a written informed consent.

3.1.4.1 Eligibility to Proceed with PBMC Collection

- Research participant must not require more than 2 mg TID of Dexamethasone on the day of PBMC collection.
- Research participant must have appropriate venous access.
- At least 2 weeks must have elapsed since the research participant received his/her last dose of prior chemotherapy or radiation.

3.1.4.2 Eligibility to Proceed with Rickham Placement

- Adequate renal function as evidenced by creatinine <1.6 mg/dL;
- Adequate bone marrow function as evidenced by WBC > 2,000/dl (or ANC>1,000) and platelets ≥ 100,000/dl, and INR<1.3;
- Normal liver function as evidenced by bilirubin <1.5 mg/dL, and ALT and AST<2.5X upper limits of normal;
- An interval of at least 12 weeks must have elapsed since the completion of initial radiation therapy;
- Wash-out requirements (standard or investigational):
    - At least 6 weeks since the completion of a nitrosourea-containing chemotherapy regimen; and
    - At least 23 days since the completion of Temodar and/or 4 weeks for any other non-nitrosourea-containing cytotoxic chemotherapy regimen. If a patient's most recent treatment was with a targeted agent only, and s/he has recovered from any toxicity of this targeted agent, then a waiting period of only 2 weeks is needed from the last dose and the start of study treatment, with the exception of bevacizumab where a wash out period of at least 4 weeks is required before starting study treatment.

3.1.4.3 Eligibility for Enrollment and to Proceed with CAR T Cell Infusion

Research participants will enroll to the study once they meet Eligibility to Proceed with CAR T Cell Infusion prior to Cycle 1. Once enrolled, subjects will be considered on ‘active’ treatment until they complete the DLT evaluation period (~28-days).

- Research participant has a released cryopreserved T cell product.
- Pulmonary: Research participant does not require supplemental oxygen to keep saturation greater than 95% and/or does not have presence of any radiographic abnormalities on chest x-ray that are progressive.
- Cardiac: Research participant does not require pressor support and/or does not have symptomatic cardiac arrhythmias.
- Active Infection: Research participant does not have a fever exceeding 38.5°C; there is an absence of positive blood cultures for bacteria, fungus, or virus within 48-hours prior to T cell infusion and/or there aren’t any indications of meningitis.
- Hepatic: Research participant serum total bilirubin or transaminases does not exceed 2X normal limit.
- Renal: Research participant serum creatinine $\leq$1.8 mg/dL.
- Neurologic: Research participant does not have uncontrolled seizure activity following surgery prior to starting the first T cell dose.
- Hematologic: Research participant platelet count must be $\geq$100,000. However, if platelet level is between 75,000-99,000, then T-cell infusion may proceed after platelet transfusion is given and the post transfusion platelet count is $\geq$100,000.
- Steroid Dependency: Research participants must not require more than 2 mg TID of Dexamethasone during T cell therapy.

3.2 Screening Exclusion Criteria

3.2.1 Organ Function:

- Pulmonary: Research participant requires supplemental oxygen to keep saturation greater than 95% and the situation is not expected to resolve within 2 weeks.
- Cardiac: Research participant requires pressor support and/or has symptomatic cardiac arrhythmias.
- Renal: Research participant requires dialysis.
- Neurologic: Research participant has uncontrolled seizure activity and/or clinically evident progressive encephalopathy.

3.2.2 Study-Specific Exclusions

- Failure of research participant to understand the basic elements of the protocol and/or the risks/benefits of participating in this phase I study. A legal guardian may substitute for the research participant.
- Research participants with any non-malignant intercurrent illness which is either poorly controlled with currently available treatment, or which is of such severity that the investigators deem it unwise to enter the research participant on protocol shall be ineligible.
- Research participants with any other active malignancies.

- Research participants being treated for severe infection or who are recovering from major surgery are ineligible until recovery is deemed complete by the investigator.
- Research participants with any uncontrolled illness including ongoing or active infection. Research participants with known active hepatitis B or C infection; research participants with any signs or symptoms of active infection, positive blood cultures or radiological evidence of infections.
- Research participants who have confirmed HIV positivity within 4 weeks of screening.

3.2.3 Non-Compliance

Subjects, who in the opinion of the investigator, may not be able to comply with the safety monitoring requirements of the study.

3.3 Inclusion of Women and Minorities

The study is open to anyone regardless of gender or ethnicity. Efforts will be made to extend the accrual to a representative population, but in a trial which will accrue approximately 23 participants on each of the remaining four arms for a total of 92 participants, a balance must be struck between subject safety considerations and limitations on the number of individuals exposed to potentially toxic or ineffective treatments on the one hand and the need to explore gender, racial, and ethnic aspects of clinical research on the other. If differences in outcome that correlate to gender, racial, or ethnic identity are noted, accrual may be expanded or additional studies may be performed to investigate those differences more fully.

4.0 Screening and Registration Procedures

4.1 Screening Procedures

A screening consent will be utilized to consent participants to allow for screening their existing tumor tissue for IL13Rα2+ expression. Once the IL13Rα2+ expression is confirmed and participants decide to proceed with the treatment portion of the study, they will be asked to sign the main consent. All other diagnostic or laboratory studies performed exclusively to determine eligibility for this trial will be done only after obtaining written informed consent for the main consent. Studies or procedures that were for clinical indications (not exclusively to determine study eligibility) may be used for baseline values, even if the studies were done before informed consent was obtained. Reference is made to **Section 10.0** – Study Calendar.

4.2 Informed Consent

The investigational nature and objectives of the trial, the procedures and treatments involved and their attendant risks and discomforts, and potential alternative therapies will be carefully explained to the subject and a signed informed consent will be obtained. Documentation of informed consent will be maintained in the subject's research chart and medical record.

4.3 Registration Requirements/Process

Research participants will be identified through the clinical practices of the PI, co-Is and participating clinicians and through direct referrals from outside hospitals and physicians. No direct-to-patient advertising will be performed without IRB approval.

The following criteria will be used for pre- screening:

- History and/or verification of high-grade glioma: History of high-grade glioma (WHO grade III or IV) or has a prior, histologically-confirmed diagnosis of a grade II glioma and now has radiographic findings consistent with a high grade glioma (grade III or IV) after completing standard therapy.

- Imaging and/or Histopathological Confirmation: Imaging and/or histopathological confirmation of recurrent disease, or verification of “high risk” histology.
- IL13Rα2 expression: Potential research participants will request COH Pathology Core to perform prescreening immunohistochemistry for IL13Rα2 tumor expression after signing the screening consent. IL13Rα2 tumor expression performed on paraffin-embedded tumor specimen will be evaluated by COH Clinical Pathology by immunohistochemistry. Tumor expression will be scored on a scale of 0 to 3 with 0 representing negative, 1+ low, 2+ moderate and 3+ high staining intensity. The criteria for inclusion will be at least 20% of the cells scoring 1+ staining intensity (≥ 20%, 1+).

After research participants have signed the main informed consent, met all screening criteria and have undergone leukapheresis procedure that yielded a CAR T cell product for infusion, has undergone Rickham catheter placement, and has been scheduled for Cycle 1 of CAR T cell infusion they will be registered to the study. The research participant will be registered (and marked as “active”) at the time Cycle 1 CAR T cell infusion starts.

4.4 Randomization and/or Dose Level Assignment

Reference **Section 13.2,** Dose Escalation Rules**.**

5.0 Treatment Program

5.1 Treatment Overview

This will be a Phase I, open-label, non-randomized trial with 15 participants in the dose escalation portion of the trial, plus 6 in the expansion portion plus 2 for replacement of unevaluable participants giving an expected sample size of 23 per arm for each of the 4 open arms for a total of 92. As of 03/02/2018, we have closed Arm 1 (biopsy with an N=2) as biopsy participants can be included in the ICV or dual arms. Research participants with recurrent/progressive high-grade glioma will receive an initial low dose (cycle 1) followed by 2 additional infusions at the 5-fold higher cell dose (cycles 2 and 3) of autologous IL13(EQ)BBζ/CD19t+ T cells (‘CAR T cells’) delivered to the tumor bed (Arm 1 = IL13(EQ)BBζ/CD19t+ T_{CM} intratumoral delivery), tumor cavity (Arm 2 = IL13(EQ)BBζ/CD19t+ T_{CM} intracavitary delivery), into the lateral ventricles (Arm 3 = IL13(EQ)BBζ/CD19t+ T_{CM} intraventricular delivery), into either the tumor bed or cavity (intratumoral) and into the lateral ventricles (intraventricular) (Arm 4 = IL13(EQ)BBζ/CD19t+ T_{CM} dual delivery, and Arm 5 = IL13(EQ)BBζ/CD19t+ $T_{N/MEM}$ dual delivery) via indwelling catheter(s).

If participants continue to meet eligibility criteria and have sufficient CAR T cell doses available they may receive additional cycles (cycles 4+) at the highest tolerated cell dose that was studied and deemed to be safe. If a research participant progresses following completion of the intracranial administration (intracavitary or intratumoral), at the discretion of the PI, the research participant may move to intraventricular administration of CAR T cells for additional CAR T cell infusions and may receive the highest dose deemed safe for that delivery method (reference **Section 13.3**) as long as the participant continues to meet eligibility criteria. For subjects initially treated on Arms 4 or 5, should the PI or designee decide to treat at only 1 delivery site, then during the additional cycles, the infusion doses may be delivered at up to the highest dose deemed safe for that delivery method.

5.1.1 Schedule

For a tabular view of the treatment, monitoring, and follow-up schedule as well as allowable evaluation windows (e.g., +/- 7 days), see study calendar in **Section 10**.

The study treatment outline will include the following steps:

5.1.1.1 Screening Evaluation

Evaluation of research participant eligibility after signing the informed consent will include the following. However, procedures completed within 6 weeks of signing the informed consent will not be duplicated and will, therefore, be used to document eligibility:

- Review by COH pathologists of available pathologic specimens to confirm history of high-grade glioma.
- Review of available MRI and PET images.
- COH clinical pathology confirms IL13Rα2+ tumor expression by immunohistochemistry (≥ 20%, 1+).
- History and Physical Exam, Vital Signs, KPS.
- Females and males of reproductive potential must use a physician-approved contraceptive method for at least two weeks prior to, during, and two months after final T cell infusion.
- Serum pregnancy test for post-menarchal women without a history of tubal ligation or hysterectomy
-
- Initiation of the following tests:
    - HIV Ag/Ab Combo Assay
    - CBC, Differential, Platelet Count
    - Comprehensive Metabolic Panel
    - QuantiFERON-TB Gold or equivalent
- Peripheral Blood Correlative Draw

5.1.1.2 PBMC Collection for CAR T Cell Manufacturing

PBMC collection will take place at the COH Donor Apheresis Center (DAC) according to DAC operating procedures. Research participants must be evaluated by a physician prior to PBMC collection and not have a standard contraindication for this procedure per COH standard practices. The evaluation process may take approximately 1 hour. If the research participant has appropriate venous access they will be scheduled for a leukapheresis procedure, however, if they do not then they may require a temporary catheter for central venous access. Please refer to **Section 3.1.4.1** for eligibility criteria to proceed with PBMC collection.

A leukapheresis procedure will be a single apheresis run of approximately 2-4 hours. Apheresis duration may be modified by the DAC physician as required by DAC policies without prior notification to the PI and will not result in a deviation. Should a technical issue arise during the procedure or in the immediate processing of the product, such that it cannot be used for CAR T cell production, or if the PI determines that the patient will benefit from additional cell product manufacturing additional procedures may be ordered.

5.1.1.3 Preparation for Biopsy/Resection and Rickham Placement

Research participants will be evaluated by standard surgical policies and procedures for eligibility to proceed to Rickham reservoir/catheter placement into the tumor bed (intratumoral) and/or into the tumor cavity (intracavitary). In addition, for research participants who will receive intraventricular (ICV) delivery of CAR T cells, the Rickham reservoir/catheter will be placed into the lateral ventricle. Should patient material be available in excess of that needed for standard pathologic studies, this excess material will be used for correlative studies assessing therapeutic responses to include but not be limited to tumor IL13Rα2

expression levels and susceptibility to re-directed CAR T cell effector mechanisms, CAR T cell persistence, and anti-therapy immune responses. During this evaluation time and prior to surgical procedures, the following must be performed:

- Vital Signs, KPS
- EORTC QLQ-C30 Questionnaire
- CBC, Differential, Platelet Count
- Comprehensive Metabolic Panel
- 35 cc peripheral blood will be collected for Correlative Studies (drawn prior to surgical procedure) including, but not limited to, the following:
    - *Ex vivo* Immunophenotyping/Functional Analysis

5.1.1.4 *Post Surgery Evaluations*

Following biopsy/resection/Rickham placement, but before initiation of CAR T cell infusions, the research participants will be evaluated for the following baseline studies (*required 1-2 weeks before initiating the CAR T cell infusions*):

- FDG-PET scan
- MRI +/- contrast

5.1.1.5 *Adoptive Transfer of Cryopreserved CAR T Cell Product*

Prior to Cycle 1 infusion, research participants will be evaluated for study enrollment, and then prior to each cycle, research participants will be evaluated for CAR T cell infusion eligibility (reference **Section 3.1.4.2**). CAR T cell infusion may be delayed no more than 2-weeks without resulting in a DLT (reference **Table 13.2**). No doses will be skipped (reference **Section 13.1 Study Design** for dosing rules).

- Progress notes including vital signs (pulse rate, temperature, respiratory rate, blood pressure)
- KPS
- EORTC QLQ-C30 Questionnaire (REQUIRED for Cycles 1, 2 and 3 while elective for additional cycles)
- Complete blood count, differential, platelet count
- Comprehensive Metabolic Panel
- 35 cc peripheral blood will be collected for Correlative Studies (drawn prior to CAR T cell infusion) including, but not limited to, the following:
    - WPRE Q-PCR
    - *Ex vivo* Immunophenotyping/Functional Analysis
- When feasible, tumor cavity fluid/CSF will be aspirated from research participants for Correlative Studies.
- CTCAE evaluation using version 4 and the revised CRS grading system (**Appendix 16.3**)

After a research participant is deemed eligible to proceed with CAR T cell infusion, the PI or designee will alert the T cell Manufacturing Supervisor and COH Quality Systems to initiate preparation of the CAR T cell infusion product.

On the morning of CAR T cell infusion, a sample of the CAR T cell product will be sent for sterility analysis. Results will be obtained after the product has been infused. In the event that there is a positive microbiology culture, the PI or designee must be notified immediately. Standard of care will be followed

to treat research participants having a bacterial, fungal or viral infection in addition to standard prophylaxis, with the agent chosen to cover the specific organism identified while taking into account any patient specific antibiotic allergies. Treatment will be at the PI or designee's discretion.

Research participants will be pre-medicated approximately 30 minutes prior to CAR T cell infusion with 15 mg/kg of acetaminophen P.O. (max. 650 mg), and diphenhydramine 25-50 mg I.V. or PO (max dose 50 mg). Clinically acceptable alternatives may be used if research participant is intolerant.

5.1.1.6 Evaluation Period

Following the completion of cycles 1 through 3, research participants will be required to undergo evaluations. Evaluations for adverse events will be made during each cycle and 1 week following the 3rd cycle. Assessments for disease response will be required to be completed within 2 weeks from Cycle #3 at the discretion of the PI/designee:

- Progress notes including vital signs (pulse rate, temperature, respiratory rate, blood pressure)
- KPS
- EORTC QLQ-C30 Questionnaire
- Complete blood count, differential, platelet count
- Comprehensive Metabolic Panel
- CRP
- FDG-PET
- MRI +/- contrast

5.2 Planned Duration of Therapy

Therapy as defined on this study consists of three, weekly, dose escalating autologous IL13(EQ)BBζ/CD19t+ T cell infusions (CAR T cell infusions). Infusion doses will be prepared in 0.5 mL PFNS (+2% HSA).

For research participants who receive intratumoral or intracavitary delivery of CAR T cells: CAR T cells will be administered manually followed by up to 0.5 mL preservative-free normal-saline (PFNS) over approximately 5 minutes through a Rickham catheter.

For research participants who receive intraventricular delivery of CAR T cells: CAR T cells will be administered manually followed by up to 0.5 mL preservative-free normal-saline (PFNS) over approximately 5 minutes through a Rickham catheter, placed into the lateral ventricle for an intraventricular administration.

For research participants who receive dual delivery (both the intratumoral/intracavitary and intraventricular delivery) of CAR T cells: the listed CAR+ T cell doses will be delivered into both the tumor bed/cavity and the ventricle, thereby doubling the total infused dose. For example, in Dose Schedule 2 Cycle 1: $10x10^6$ CAR+ T cells will be delivered intratumorally/intracavitary and $10x10^6$ CAR+ T cells will be delivered intraventricularly for a total Cycle 1 infused dose of $20x10^6$ CAR+ T cells. The administration of each dose will follow the rules listed above.

Please note that as of 03/02/2018, we have closed Arm 1 (biopsy with an N=2) as biopsy participants can be included in the ICV or dual arms. The study will continue to enroll participants in the remaining 4 arms, intracavitary (Arm 2), intraventricular (Arm 3), or dual delivery (both intratumoral and intraventricular) (Arms 4 and 5).

The PFNS flush is meant to clear the administration line and 'push' remaining CAR T cells through the catheter.

Immediately following CAR T cell infusion research participants will be monitored for at least 3 hours (e.g., vital status, O_2, BP and temp).

5.2.1.1 Additional Cycles

Additional cycles of CAR T cell infusions will be offered to research participants after they complete their required evaluation period, provided the following criteria are met:

- In the initial Dose Schedule the participant received a cell dose that was well tolerated (i.e., did not experience a DLT at that dose level), and therefore may receive additional cycles via the same delivery route at or below this tolerated cell dose.
- Additional CAR T cell product is available or can be generated from excess material of the PBMC collection, and the PI feels that the participant will benefit from undergoing an additional apheresis procedure.

After first progression, a participant may continue to receive additional CAR T cycles as long as the participant continues to meet eligibility criteria. For subjects initially treated on Arms 4 or 5, should the PI or designee decide to treat at only 1 delivery site, then during the additional cycles, the infusion doses may be delivered at up to the highest dose deemed safe for that delivery method.

Note: additional leukapheresis procedures may be performed in order to manufacture additional CAR T cell doses at the PI determination and per COH Transfusion guidelines. Participants must continue to meet all eligibility criteria as listed in **Section 3.1.4.2** at the time of *each* additional infusion. Infusions via the same delivery route will be given at less than or equal to the highest tolerated dose in Cycles 1 through 3. The dose may be lowered based on available cryopreserved product.

5.3 Criteria for Removal from Treatment

Research participants may be removed from the study for any of the following reasons:

- Research Participant Premature Discontinuation

Research participants who DO NOT receive any CAR T cell infusion will be considered screen failures and will NOT be enrolled to the study.

Research participants who screen fail will be replaced. The reasons for screen failure must be recorded on the case report form and may include:

- The judgment of the PI, or his designee, is that the research participant is too ill to enroll and receive CAR T cell infusion,
- Significant and rapid progression of GBM prior to CAR T cell infusion requiring alternative medical intervention that, in the opinion of the PI or his designee, is not compatible with CAR T cell therapy. If a research participant had successful generation of a therapeutic CAR T cell product but screen failed, s/he may be eligible to enroll on the trial at a future date if they meet the eligibility criteria and a study slot is available.
- Inability to manufacture and/or release a CAR T cell product.
- Patient failure to meet eligibility criteria on **Section 3.1.4.2,** Eligibility Criteria to Enroll and Undergo Infusion of Genetically Modified Autologous T Cells (CAR T cells).
- Research participant/family noncompliance with study therapy and/or clinic appointments,
- Pregnancy,
- Voluntary withdrawal; a research participant or his/her parents/legal guardians may remove himself/herself from the study at any time without prejudice.

- Research Participant - Off Active Treatment

Research participants AFTER receiving any CAR T cell infusion would still be required to adhere to long term follow up guidelines as mandated for research participants who receive gene modified cells (follow-up for 15 years from the time a CAR T cell product is infused).

All research participants are free to withdraw from participation at any time, for any reason, specified or unspecified, and without prejudice.

Potential reasons for the study participant being taken off active treatment include:

  - The judgment of the PI, or his designee, the research participant is too ill to continue,
  - Research participant/family noncompliance with study therapy and/or clinic appointments,
  - Pregnancy,
  - Voluntary withdrawal; a research participant or his/her parents/legal guardians may remove himself/herself from the study at any time without prejudice,
  - Significant progression of GBM requiring alternative medical interventions that in the opinion of the PI or his/her, designee, are not compatible with CAR T cell therapy. Research participants who have progressive disease may begin the modified follow-up evaluations outlined in **Section 10.3** until completion of the 1 year (12 month) follow-up period at which time they will be followed in accordance with the FDA guidance "Gene Therapy Clinical Trials – Observing Subjects for Adverse Events."

- CAR T Cell Infusion *cancelled* for the following reasons:
  - Patient failure to meet eligibility criteria on **Section 3.1.4.2,** Eligibility Criteria at Time of Infusion of Genetically Modified Autologous T Cells (CAR T cells).
  - Inability to manufacture and/or release a CAR T cell product.
  - Significant and rapid progression of GBM prior to CAR T cell infusion requiring alternative medical intervention that, in the opinion of the PI or his designee, is not compatible with CAR T cell therapy.
- Other definitions include the following:
  - Off Active Treatment: this is the date when the study PI/team and/or the patient decide that he/she will no longer be receiving CAR T cells. This date is then inputted into the CTMS and EPIC systems to denote that the participant is no longer actively receiving CAR T cells.
  - Off Protocol Therapy: This is the censor date which could reflect any of the following situations:
    - Date of first progression after receiving CAR T cells.
    - The start date of disallowed therapy for participants who have not yet progressed.
    - The last date of contact for participants who have not yet progressed.

5.4 Subject Follow-Up

5.4.1 Follow-Up Post CAR T cell Infusion

- Immediate follow-up: Research participants will have follow-up evaluations (reference **Section 10.1** for details) at least twice weekly during each CAR T cell infusion week for toxicities, serum analysis, correlative studies and, when accessible, tumor cavity aspirate/CSF samples.
- In research participants who exhibit and/or experience cytokine release syndrome symptoms or focal neurological signs or mental status changes following CAR T cell therapy will be admitted to the hospital for observation, management of clinical symptoms (as applicable), and additional testing which MAY include a neurological consultation, MRI/CT, tumor cavity aspirate/CSF samples and a lumbar puncture. Subsequent correlative studies will include analysis of serum for cytokine profiles (reference **Section 10**).
- Short-term follow-up: Research participants will have follow-up evaluations at 4 weeks and 3 months post last CAR T cell infusion, subsequently at 6 months they will continue with evaluations every other month for the first year post last CAR T cell infusion for toxicities, and correlative studies (as applicable, reference **Section 10.2**).
- Long-term follow-up: Research participants will continue with annual evaluations (reference **Section 10.3** for details) for a minimum of 15 years in accordance with the guidance “Gene Therapy Clinical Trials – Observing Subjects for Adverse Events”.

5.4.2 AE and ConMed data collection:

- Research participants, who have received at least one cycle of CAR T cells, will be followed and considered on active therapy through one week post last cycle of CAR T cells for toxicity and DLT assessments. During this time, all AE grade changes and ConMeds (as new, ongoing or discontinued) will be captured regardless of disease status or additional therapy (reference **Section 10.1**).
- During short term follow-up, AEs (including worst grade and attribution) and ConMeds (as new, ongoing or discontinued) will be captured at the set evaluation periods for the first year (reference **Section 10.2**).
- Once the participant has completed one week post last cycle of CAR T cells or if they undergo one or more of the following: (1) receives radiation and/or high dose steroids, and/or (2) has documented disease progression, the research participant may be taken off active therapy and continue to be followed as specified below:
  - In the event a research participant undergoes either radiation and/or high dose steroids, the research participant may then continue to be followed per **Section 10.3** where only select AEs attributable to gene therapy are collected while ConMed collection is no longer required. At the end of the first year post last CAR T cell infusion, the research participant will continue to be followed per FDA guidance on gene therapy clinical trials.
  - In the event a research participant is found to have progressive disease, the research participant may continue to be followed per **Section 10.3**, where only select AEs attributable to gene therapy are collected while ConMed collection is no longer required. At the end of the first year post last CAR T cell infusion, the research participant will continue to be followed per FDA guidance on gene therapy clinical trials.

5.5 Supportive Care, Other Concomitant Therapy, Prohibited Medications

5.5.1 Supportive Care for Regimen Related Toxicities

All standard supportive care measures for patients with recurrent/progressive GBM will be used at the discretion of the research participant's COH oncologist.

5.5.1.1 Contra-Indicated Medications

Unless the Principal Investigator or his designee provides an exception, the following agents, other than specified in the protocol, are not allowed once CAR T cell infusion commences through completion of the DLT Evaluation Period (unless otherwise specified):

- chemotherapy
- immunosuppressive agents, with the following exceptions:
  - Start of CAR T cell Cycles through completion of Cycle 3: $\leq$2 mg TID of Dexamethasone,
  - During Rest Week: $\leq$4 mg QID of Dexamethasone
- immunotherapy
- other investigational agents

5.5.2 Management of Constitutional Symptoms Associated with CAR T cell Infusion

5.5.2.1 Fever, and Chills: Fever, chills and temperature elevations >101°F will be managed with additional Tylenol (or equivalent) as clinically indicated. Demerol I.V. (max dose 50 mg) may be given for chills. Additional methods such as cooling blankets may be employed for fevers resistant to these measures. Research participants that develop fever or chills will have a blood culture drawn. Appropriate selection of empiric antibiotics for treatment of neutropenic fever will be administered to research participants who, in the opinion of the physician in attendance, appear septic; alternate antibiotic choices will be used as clinically indicated.

5.5.2.2 Headache: Headaches may be managed with Tylenol (or equivalent). If unresponsive to Tylenol, manage with good clinical judgment.

5.5.2.3 Nausea and Vomiting: Nausea and vomiting will be managed with ondansetron IV or PO as well as additional standard anti-emetic treatments per patient preference.

5.5.2.4 Seizures: All patients will be started on prophylactic antiepileptic drugs (AED) prior to the surgery. However, in the event of worsening seizure activity, AED dose strength and frequency will be increased, or a second-line AED will be started.

5.5.2.5 Hypoxemia: Hypoxemia will be managed with supplemental oxygen. An etiology for hypoxemia will be worked up per standard clinical practice.

5.5.3 Management of Acute Adverse Event(s) Attributable to This Study

5.5.3.1 Management of Acute Adverse Event(s) Attributable to Infused CAR T Cells:

Potential acute toxicities experienced following CAR T cell therapy include fever, chills, CRS, and change in mental status. The following rules will be applied for such acute adverse events attributed to CAR T cell infusion:

- Research participants experiencing a new grade 3 or higher toxicity with an attribution of probable or definite to the infused CAR T cells rather than expected toxicities attributable to the surgery or catheter placement, that in the opinion of the Principal Investigator puts the research participant in significant risk of an untoward outcome if measures are not taken to ameliorate the toxicity should commence with corticosteroids and/or IL-6 antagonist Tocilizumab (reference **Section 8.1.2**).
- Additional measures may also be taken to resolve the toxicity should the protocol specified T cell toxicity treatment plans fail to ablate side effects associated with CAR T cell infusion such as, but not limited to, immunosuppressive medications or chemotherapy agents with immunosuppressive properties.

- If applicable, research participants will be hospitalized for at least the first 72 hours of receiving any CAR T cell toxicity treatment plan (reference **Section 8** for specific treatment schedules).

5.6 Additional Studies

Additional studies will include relevant laboratory tests for screening and baseline evaluations as well as applicable laboratory tests to be performed during the follow up period.

Reference is made to **Section 9.0** for any correlative studies to be conducted under this protocol.

5.6.1 Laboratory Studies

Laboratory evaluations will be performed described in **Section 10**.

5.6.2 Long-Term Follow-Up

Research participants treated on this study are required to be followed for 15 years to monitor potential late effects of gene therapy as specified in the guidance "Gene Therapy Clinical Trials – Observing Subjects for Delayed Adverse Events'.

Long term follow-up will include the following:

- Physical examination which will include assessment for reproductive risk and risk to the fetus; autoimmune toxicities (such as new evidence or exacerbation of a prior rheumatologic or other autoimmune disorder);
- Continued annual testing with an HIV test by qPCR, or equivalent as determined by the LTFU PI, for the diagnosis of HIV positivity which could result from an RCL mediated event from the investigational agent (initial testing at the pre, 3, 6, and 12 month time points previously required under COH IRB 13384 follow-up, reference **Section 10**);
  - In the event of a confirmed positive HIV result COH will require further testing to interrogate for evidence of a recombination event due to the investigational agent to distinguish from HIV infection. This will include qPCR testing for VSV-G and transgene.
  - Reports of all confirmed positive HIV results must be submitted to the FDA. Contact the Office of IND Development and Regulatory Affairs for such reporting requirements.
- Evaluation of CAR T cell persistence which will include testing for long-term vector persistence and insertional mutagenesis until at such time CAR T cells become undetectable.
  - In the event of continued CAR T cell persistence, the participant will be asked to return to COH so that additional peripheral blood samples may be evaluated for clonal T cell population(s), including the gene expression profile (i.e., insertional site mutagenesis) and the cytokine independent growth potential of any such population.

All long-term toxicities will be reported to the Agency in accordance with CFR 312.32.

In addition, research participants will be requested to send all future brain imaging studies for review by the investigative team. Autopsy reports and available tissues/specimens will be sought on expired research participants who consent to this procedure.

5.7 Definition of Dose-Limiting Toxicity (DLT)

A dose limiting toxicity will be defined as events attributable to the T-cell infusion (probable or definite) with the exception of those listed in **Section 8.1.2**, and occurring from the time of initial CAR T cell infusion through 1 week following the last infusion cycle (not including the optional cycles) unless otherwise specified within this definition:

a) Two grade 3 toxicities at the same dose with the exception of those grade 3 toxicities listed below;

b) Any grade 3 CRS toxicity lasting more than 72 hours with intervention;
c) Any grade 3 or higher allergic reaction;
d) Any grade 3 or higher autoimmune reaction;
e) Any grade 4 toxicity

Note: *each treatment arm will be followed and analyzed separately.*

6.0 Dose Delays/Modifications for Adverse Events

A single CAR T cell infusion will be performed at each delivery site, as applicable, for each cycle. This is an intrapatient and interpatient dose escalation study where dose delays and dose modifications are defined in **Section 13.2** as part of the study design.

7.0 Data and Safety Monitoring, Unanticipated Problems and Adverse Event Reporting

7.1 Data and Safety Monitoring

This is a Risk Level 4 study, as defined in the City of Hope Institutional Data and Safety Monitoring Plan [policy dated 07/09/2014]. This determination was made because the study involves COH as the IND holder and a first-in-human gene therapy for *ex vivo* expanded autologous IL13(EQ)BBζ/CD19t+ T Cells for patients with recurrent/refractory high-grade malignant glioma.

7.2 Monitoring and Personnel Responsible for Monitoring

The Protocol Management Team (PMT) is responsible for monitoring the data and safety of this study. The PMT consists of the Principal Investigator (PI), Collaborating Investigator, Biostatistician, Research Protocol Nurse, and Clinical Research Coordinator. is responsible for monitoring the data and safety of this study, including implementation of the stopping rules for safety and efficacy.

The PMT is required to submit periodic status reports (i.e., the PMT Report) according to the frequency prescribed in the City of Hope Institutional Data and Safety Monitoring Plan [policy dated 07/09/2014]. Important decisions made during PMT meetings (i.e., dose escalation, de-escalation, etc.) only need to be noted in the PMT Report submitted to the Data and Safety Monitoring Committee (DSMC).

This study will utilize the Phase I tracking log to monitor data and safety for dose escalation**.** The tracking log will contain dose levels administered, dose limiting toxicities (DLT), DLT-defining adverse events, and any details regarding dose level escalation. The record of doses administered and resultant adverse events will be included in the PMT Report.

7.3 Definitions

7.3.1 Adverse Events and Serious Adverse Events

The PI will be responsible for determining the event name, assessing the severity (i.e., grade), expectedness, and attribution of all adverse events.

7.3.2 Adverse event (AE):

An adverse event is any untoward medical experience or change of an existing condition that occurs during or after treatment, whether or not it is considered to be related to the protocol intervention.

7.3.3 Reporting Non-serious Adverse Events

Adverse events will be collected after the patient is given the study treatment. Adverse events will be monitored by the PMT. Adverse events that do not meet the criteria of serious OR are not unanticipated problems will be reported only in the PMT Report.

7.3.4 Serious Adverse Event (SAE) [21 CFR 312.32]:

[Modified from the definition of unexpected adverse drug experience in 21 CFR 312.32] - defined as *any expected or unexpected adverse event* that results in any of the following outcomes:

- Death
- Is life-threatening experiences (places the subject at immediate risk of death from the event as it occurred);
- Unplanned hospitalization equal or greater than 24 hours or prolongation of existing hospitalization;
- A persistent or significant disability/incapacity;
- A congenital anomaly/birth defect, or
- Secondary Malignancy.

Any other adverse event that, based upon appropriate medical judgment, may jeopardize the subject's health and may require medical or surgical intervention to prevent one of the outcomes listed above (examples of such events include allergic bronchospasm requiring intensive treatment in the emergency room or at home, blood dyscrasisas of convulsions that do not result in inpatient hospitalization, or the development of drug dependency or drug abuse).

7.3.5 Reporting Serious Adverse Events

Begins at the start of study treatment: All SAEs occurring during this study, whether observed by the physician, nurse, or reported by the patient, will be reported according to the approved City of Hope's Institutional policy [policy effective date: 05/14/14]. Serious Adverse Events that require expedited reporting will be submitted electronically using iRIS.

7.3.6 Adverse Event Name and Severity

The PI will determine the adverse event name and severity (grade) by using the CTCAE v4.0, the revised CRS grading system (**Section Appendix 16.3**) as well as the modified neurological grading system (Section **16.1, Appendix A**) from data obtained at each clinical assessment as outlined in **Section 10**.

7.3.7 Expected Adverse Event:

Any event that does not meet the criteria for an unexpected event OR is an expected natural progression of any underlying disease, disorder, condition, or predisposed risk factor of the research participant experiencing the adverse event.

7.3.8 Unexpected Adverse Event [21 CFR 312.32 (a)]:

An adverse event is unexpected if it is not listed in the investigator's brochure and/or package insert; is not listed at the specificity or severity that has been observed; is not consistent with the risk information described in the protocol and/or consent; is not an expected natural progression of any underlying disease, disorder, condition, or predisposed risk factor of the research participant experiencing the adverse event.

7.3.9 Adverse Event Attribution

The following definitions will be used to determine the causality (attribution) of the event to the study agent or study procedure.

Definite - The AE is clearly related to the investigational agent or study procedure and unrelated to any other cause.

Probable - The AE is likely related to the investigational agent or study procedure and unlikely related to other cause(s).

Possible -The AE may be related to the investigational agent or study procedure and may be related to another cause(s).

Unlikely -The AE is doubtfully related to the investigational agent or study procedure and likely related to another cause(s).

Unrelated -The AE is clearly not related to the investigational agent or study procedure and is attributable to another cause(s).

7.3.10 COH Held IND

Serious Adverse Events meeting the requirements for expedited reporting to the Food and Drug Administration (FDA), as defined in 21 CFR 312.32, will be reported as an IND safety report using the MedWatch Form FDA 3500A for Mandatory Reporting.

The criteria that require reporting using the Medwatch 3500A are:

- Any unexpected fatal or life threatening adverse experience associated with use of the drug must be reported to the FDA no later than 7 calendar days after initial receipt of the information [21 CFR 312.32(c)(2)]
- Any adverse experience associated with use of the drug that is both serious and unexpected must be submitted no later than 15 calendar days after initial receipt of the information [21 CFR 312.32(c)(1)]
- Any follow-up information to a study report shall be reported as soon as the relevant information becomes available. [21 CFR 312.32(d)(3)]

The PI or designee will be responsible for contacting the Office of IND Development and Regulatory Affairs (OIDRA) at COH to ensure prompt reporting of safety reports to the FDA. OIDRA will assist the PI with the preparation of the report and submit the report to the FDA in accordance with the approved City of Hope's Institutional policy [policy effective date: 05/14/14].

7.3.10.1 Deviations and Unanticipated Problems

Deviation - A deviation is a divergence from a specific element of a protocol that occurred without prior IRB approval. Investigators may deviate from the protocol to eliminate immediate hazard(s) for the protection, safety, and well-being of the study subjects without prior IRB approval. For any such deviation, the PI will notify the COH DSMC and IRB within 5 calendar days of its occurrence via iRIS in accordance with the Clinical Research Protocol Deviation policy [policy effective date: 11/07/11].

Single Subject Exception (SSE) - An SSE is a planned deviation, meaning that it involves circumstances in which the specific procedures called for in a protocol are not in the best interests of a specific patient. It is a deviation that is anticipated and receives prior approval by the PI and the IRB. The SSE must be submitted as a "Single Subject Exception Amendment Request" via iRIS in accordance with IRB guidelines and the Clinical Research Protocol Deviation policy [policy effective date: 11/07/11]. An IRB approved SSE does not need to be submitted as a deviation to the DSMC.

Unanticipated problem (UP):

Any incident, experience or outcome that **meets all three** of the following criteria:

1. Unexpected (in term nature, severity, or frequency) given the following: a) the research procedures described in the protocol-related documents such as the IRB approved research protocol, informed consent document or Investigator Brochure (IB); and b) the characteristics of the subject population being studied; **AND**
2. Related or possibly related to participation in the research (possibly related means there is a reasonable possibility that the incident, experience, or outcomes may have been caused by the drugs, devices or procedures involved in the research); **AND**
3. Suggests that the research places subjects or others at greater risk of harm (including physical, psychological, economic, or social harm) than previously known or recognized.

Any UP that occurs during study conduct will be reported to the DSMC and IRB in accordance with the City of Hope's Institutional policy [policy effective date: 05/14/14] using iRIS.

7.3.10.2 ADDITIONAL REPORTING REQUIRMENTS

The Office of IND Development and Regulatory Affairs (OIDRA) will assist the PI in reporting the event to the Food and Drug Administration (FDA).

Studies Involving Gene Therapy and COH Held IND

As this study involves gene therapy and a COH held IND, the PI is required to report adverse events using iRIS. The Office of IND Development and Regulatory Affairs (OIDRA) will be responsible for reporting the adverse event to National Institutes of Health-Office of Biotechnology Activities (NIH-OBA) using the MedWatch Form FDA 3500.

Long Term Follow-Up of Gene Therapy Studies

Study patients will be followed long term for up to 15 years in accordance with the guidance "Gene Therapy Clinical Trials – Observing Subjects for Adverse Events" (reference **Section 10**). It is the PI's responsibility to continue reporting data and safety concerns to the FDA, DSMC, and IRB as they arise during the follow-up period.

8.0 Agent Information and Risks

8.1 IL13(EQ)BBζ/CD19t+ T Cells (*BB-IND 16226*)

8.1.1 Description

The investigational agent in this protocol is IL13(EQ)BBζ/CD19t+ T Cells. Autologous T cells will be isolated from patient's PBMC collection after ficoll separation. Peripheral blood mononuclear cell (PBMC) preparations will then undergo immunomagnetic selection to derive T_{CM} enriched (Arms 1-4) or $T_{N/MEM}$ enriched (Arm 5) T cell preparations. The resulting cell preparation, highly enriched for $CD45RO^+CD62L^+$ T_{CM}, or $CD62L^+$ $T_{N/MEM}$, will then be activated with anti-CD3/CD28 beads. Activated T_{CM} or $T_{N/MEM}$ (as applicable) will undergo a lentiviral transduction to express the human IL13(EQ)BBζ CAR and human CD19t transgenes (description of the lentiviral vector is provided below), expanded *in vitro* to achieve cell numbers sufficient for the research participant's planned clinical cell dose and all related product release testing, and then harvested, washed and formulated for cryopreservation until time of infusion.

Lentiviral Vector IL13(EQ)BBZ-T2A-CD19t _epHIV7:Clinical grade IL13(EQ)BBZ-T2A-CD19t _epHIV7 lentiviral vector was produced and released by COH Center for Biomedicine and Genetics (reference BB-MF 9778). All raw materials used for the production of the lentiviral vector were released by the Quality Assurance (QA) department of CBG prior to use. Certificates of Analysis (CofAs) for all materials referenced in any lentiviral production batch record, as well as completion of batch records, labeling and tracking of the product are maintained by the Center for Applied Technology Development, Department of Quality Systems. All processes were carried out according to SOPs and include QA oversight for cGMP compliance. The cloned DNA that will be used for the genetic modification of autologous T cells consists of a IL13Rα2-specific, hinge-optimized, costimulatory chimeric immunoreceptor sequence (designated IL13(EQ)BBZ), a ribosome-skip T2A sequence,[69] and a CD19t sequence. The IL13(EQ)BBZ sequence was generated by fusion of the human GM-CSF receptor alpha leader peptide with human IL13(E13Y) ligand,[5] L235E/N297Q-modified human IgG4 Fc hinge (where the double mutation interferes with FcR recognition[49]), human CD4 transmembrane, human 4-1BB cytoplasmic signaling domain, and human CD3ζ cytoplasmic signaling domain sequences. This sequence was synthesized *de novo* by Geneart after codon optimization. The T2A sequence was obtained from digestion of a T2A-containing plasmid. The CD19t sequence was obtained from that spanning the leader peptide sequence to the transmembrane components (i.e., base pairs 1-972) of a human CD19-containing plasmid. All three fragments, 1)

IL13(EQ)BBZ, 2) T2A, and 3) CD19t, were cloned into the multiple cloning site of the epHIV7 lentiviral vector, so that its transcription is driven by the EF1 promoter. The IL13(EQ)BBZ-T2A-CD19t _epHIV7 construct does not contain an intact 3' long terminal repeat (LTR) promoter, so the resulting expressed and reverse transcribed DNA proviral genome in targeted cells will have inactive LTRs. As a result of this design, no HIV-I derived sequences will be transcribed from the provirus and only the therapeutic IL13(EQ)BBZ and CD19t sequences will be expressed. The removal of the LTR promoter activity in this self-inactivating vector is also expected to significantly reduce the possibility of unintentional activation of host genes.[70] Furthermore, this study will employ replication-incompetent lentivirus produced by the 4-plasmid co-transfection of producer cells (i.e., 293T renal carcinoma cells).Briefly, the crippled IL13(EQ)BBZ-T2A-CD19t _epHIV7 containing lentivirus was harvested from cultures of 293T cells that had been transiently transfected with the following four plasmids encoding the required components: 1) pCgp containing the HIV-1 gag and pol genes required for viral vector assembly; 2) pCMV-G containing the VSV-G gene required for viral vector infectivity; 3) pCMV-rev containing the rev gene which assists in the transportation of the viral genome for efficient packaging; and 4) the above described IL13(EQ)BBZ-T2A-CD19t _epHIV7 transfer vector.

8.1.2 Toxicology

Anticipated "expected" adverse events associated with the intracranial infusion of genetically modified T cells (CAR T cells) include:

- ≤ Grade 3 Dyspnea lasting up to 72 hours with intervention
- Grade 3 Fever lasting up to 5 days with intervention
- Grade 4 Fever for less than 72 hours with intervention
- ≤ Grade 2 Chills lasting for less than 72 hours with intervention
- Grade 3 Tachycardia not responding to intervention and lasting up to 72 hours
- ≤ Grade 3 Hypotension not responding to intervention and lasting up to 72 hours
- ≤ Grade 3 Headache lasting for less than 48 hours
- ≤ Grade 3 Nausea/vomiting lasting for less than 48 hours
- ≤ Grade 3 Neurological toxicities
- ≤ Grade 3 Rash lasting up to 72 hours with intervention
- Cytokine Release Syndrome
  - Grade 2 Cytokine Release Syndrome lasting up to 5 days with intervention
  - Grade 3 or 4 CRS with hypotension alone requiring a single vasopressor for support (not requiring intubation) that resolves to < Grade 3 in ≤72 hours
  - Grade 3 or 4 hypotension (without other CRS symptoms) requiring a single vasopressor for support that resolves to < Grade 3 in ≤72 hours
  - Grade 3 encephalopathy for ≤7 days and resolves to baseline in ≤28 days

  As cytokine release syndrome consists of a combination of toxicities and can only be diagnosed through exclusion it is believed that a grade 2 event lasting up to 5 days or a grade 3 event lasting up to 3 days are toxicities that can both be managed and diagnosed within the given timeframes. In the event either grade continues beyond the allowable timeframe with intervention the event will be considered a DLT (Reference **Appendix 16.3**).

*Allowable 'expected' AEs will not result in administration of CAR T cell ablation methods, and will not be considered a DLT.

Adverse events from the above list occurring within the specified time will:

- Not result in an expedited adverse event reporting to the FDA
- Not result in ablation of CAR T cells with corticosteroids

Management of Acute Toxicities associated with CAR T cell activity (i.e., grade 3 and above cytokine release syndrome, or macrophage activation syndrome) requiring the treatment of CAR T cell related side effects the participant will receive supportive management including Tocilizumab (using computerized order entry, reference **Section 8.4**), and/or high dose steroids. Recent literature supports the option of a rapid steroid taper within several days without recurrence of the CRS[71]. Dosing and choice of corticosteroid will be tailored to the individual patient, at the discretion of the principle investigator. Commonly used initial doses include methylprednisolone (2 mg/kg/day), which may be weaned over several days, depending on response to therapy. Alternatively, consideration may be given to using dexamethasone (0.5 mg/kg; maximum, 10 mg/dose) due to more efficient penetration of the blood:brain barrier. Evidence for differential efficacy of methylprednisolone vs. dexamethasone in this setting has not been established. Reference is also made to the COH CRS Management SOP.

Management of Chronic Toxicities associated with transferred CAR T cell (insertional mutagenesis, tumorigenesis of infused CAR T cells) requiring the ablation of CAR T cells, high dose steroids can be given. Chemotherapy treatment of the treatment physician's choice is also allowed to eradicate aberrant CAR T cells.

8.1.3 Pharmacology – Handling, Storage, Dispensing and Disposal

The IL13(EQ)BBζ/CD19t+ T Cell product will be prepared under BB-IND 16226 by the T Cell Therapeutics Research Laboratory (TCTRL) staff under the direction of Dr. Stephen Forman. Cell products are prepared using approved standard operating procedures and manufactured under cGMP conditions on COH campus as described in **Section 8.1**. All raw materials used for the production of the cell product are released by the QA department of CBG prior to use. Certificates of Analysis for all materials referenced in any cell product batch record and all records for T cell manipulations, including cell selection and transduction, as well as completion of batch records, labeling and tracking of the cell product will be maintained by the Center for Applied Technology Development, Department of Quality Systems. All processes are carried out according to SOPs and include QA oversight for cGMP compliance. Cryopreserved cell products as well as prepared infusion doses are Quality Control tested as required under IND and released for use by the Director of Quality Systems, or designee, Center for Applied Technology Development (CATD).

Cryopreserved vials will be frozen using a controlled rate freezer and stored in vapor phase in a controlled access LN_2 freezer until released for clinical use. The required number of cryopreserved vials will be thawed, cooled and washed with a PBS/2% human serum albumin (HSA) Wash Buffer. After centrifugation, the supernatant will be removed and the cells resuspended in a Preservative-Free Normal Saline (PFNS)/ 2% HSA infusion diluent.

Research participants will be pre-medicated approximately 30 minutes prior to CAR T cell infusion with 15 mg/kg of acetaminophen P.O. (max. 650 mg), and diphenhydramine 25-50 mg I.V. or PO (max dose 50 mg). Clinically acceptable alternatives may be used if research participant is intolerant.

Upon receipt of the PI's request for infusion preparation, the appropriate number of cryopreserved vial(s) will be thawed, washed and prepared at a concentration of no more than 0.5 x 10^6 cells/µL and released to the PI by the Director of Quality Systems, or designee. Samples of the final product will be tested for sterility as required by the IND.

Prepared infusion dose(s) will be maintained at room temperature on a rocker until transported to patient bedside by Quality Systems. Expiration date and/or time will be provided on the product label as determined by stability testing performed by TCTRL and forwarded to the FDA by formal amendment.

Any product released to the PI that is not infused to the patient will either be released back to the TCTRL for storage or discarded by clinical staff per institutional policy, and documented on the infusion record.

8.2 ACTEMRA® (Tocilizumab)

8.2.1 Description:

ACTEMRA® (tocilizumab) is indicated for the treatment of adult patients with moderately to severely active rheumatoid arthritis who have had an inadequate response to one or more Disease-Modifying Anti-Rheumatic Drugs (DMARDs), active polyarticular juvenile idiopathic arthritis in patients 2 years of age and older, and active systemic juvenile idiopathic arthritis in patients 2 years of age and older.

Tocilizumab binds specifically to both soluble and membrane-bound IL-6 receptors (sIL-6R and mIL-6R), and has been shown to inhibit IL-6-mediated signaling through these receptors. IL-6 is a pleiotropic pro-inflammatory cytokine produced by a variety of cell types including T- and B-cells, lymphocytes, monocytes and fibroblasts. IL-6 has been shown to be involved in diverse physiological processes such as T-cell activation, induction of immunoglobulin secretion, initiation of hepatic acute phase protein synthesis, and stimulation of hematopoietic precursor cell proliferation and differentiation. IL-6 is also produced by synovial and endothelial cells leading to local production of IL-6 in joints affected by inflammatory processes such as rheumatoid arthritis.

Recent clinical responses in the field of adoptive cellular immunotherapy related to the effective tumor targeting by engineered CAR T cell therapy have been associated with acute cytokine release syndrome (CRS) which often requires intensive care to manage.[54] In one case of CRS, the use of steroids did not abrogate CRS, though this was subsequently controlled by the anti-cytokine antibodies[54] implying that the antibodies not only provide an adequate alternative to steroids but that they are in fact likely a superior alternative. Furthermore, with the avoidance of steroids, these patients have been able to go on and have good responses to therapy. Although this anti-cytokine agent is associated with immune suppression when dosed repeatedly in the context of disease like arthritis or psoriasis there is little evidence to suggest risk in this on-off dosing context where the immediate benefits may well be life-saving.[54]

For this study, Tocilizumab will be used per COH institutional SOPs to manage CRS (as described in **Section 8.1** above).

8.2.2 Toxicology:

ADVERSE REACTIONS: Most common adverse reactions (incidence ≥ 5%): upper respiratory tract infections, nasopharyngitis, headache, hypertension, increased ALT.

WARNINGS and PRECAUTIONS:

Serious Infections: Serious and sometimes fatal infections due to bacterial, mycobacterial, invasive fungal, viral, protozoal, or other opportunistic pathogens have been reported in patients receiving immunosuppressive agents including ACTEMRA for rheumatoid arthritis. The most common serious infections included pneumonia, urinary tract infection, cellulitis, herpes zoster, gastroenteritis, diverticulitis, sepsis and bacterial arthritis. Among opportunistic infections, tuberculosis, cryptococcus, aspergillosis, candidiasis, and pneumocystosis were reported with ACTEMRA. Other serious infections, not reported in clinical studies, may also occur (e.g., histoplasmosis, coccidioidomycosis, listeriosis). Patients have presented with disseminated rather than localized disease, and were often taking concomitant immunosuppressants such as methotrexate or corticosteroids which in addition to rheumatoid arthritis may predispose them to infections.

ACTEMRA should not be administered in patients with an active infection, including localized infections. The risks and benefits of treatment should be considered prior to initiating ACTEMRA in patients:

- with chronic or recurrent infection;
- who have been exposed to tuberculosis;
- with a history of serious or an opportunistic infection;
- who have resided or travelled in areas of endemic tuberculosis or endemic mycoses; or
- with underlying conditions that may predispose them to infection.

Patients should be closely monitored for the development of signs and symptoms of infection during and after treatment with ACTEMRA, as signs and symptoms of acute inflammation may be lessened due to suppression of the acute phase reactants.

ACTEMRA should be interrupted if a patient develops a serious infection, an opportunistic infection, or sepsis. A patient who develops a new infection during treatment with ACTEMRA should undergo a prompt and complete diagnostic workup appropriate for an immunocompromised patient, appropriate antimicrobial therapy should be initiated, and the patient should be closely monitored

Tuberculosis: Patients should be evaluated for tuberculosis risk factors and tested for latent infection prior to initiating ACTEMRA. Anti-tuberculosis therapy should also be considered prior to initiation of ACTEMRA in patients with a past history of latent or active tuberculosis in whom an adequate course of treatment cannot be confirmed, and for patients with a negative test for latent tuberculosis but having risk factors for tuberculosis infection. Consultation with a physician with expertise in the treatment of tuberculosis is recommended to aid in the decision whether initiating anti-tuberculosis therapy is appropriate for an individual patient. Patients should be closely monitored for the development of signs and symptoms of tuberculosis including patients who tested negative for latent tuberculosis infection prior to initiating therapy. It is recommended that patients be screened for latent tuberculosis infection prior to starting ACTEMRA. The incidence of tuberculosis in worldwide clinical development programs is 0.1%. Patients with latent tuberculosis should be treated with standard antimycobacterial therapy before initiating ACTEMRA.

Viral Reactivation: Viral reactivation has been reported with immunosuppressive biologic therapies and cases of herpes zoster exacerbation were observed in clinical studies with ACTEMRA. No cases of Hepatitis B reactivation were observed in the trials; however patients who screened positive for hepatitis were excluded.

Gastrointestinal Perforations: Events of gastrointestinal perforation have been reported in clinical trials, primarily as complications of diverticulitis. ACTEMRA should be used with caution in patients who may be at increased risk for gastrointestinal perforation. Patients presenting with new onset abdominal symptoms should be evaluated promptly for early identification of gastrointestinal perforation.

Laboratory Parameters

Neutrophils

Treatment with ACTEMRA was associated with a higher incidence of neutropenia. Infections have been uncommonly reported in association with treatment-related neutropenia in long-term extension studies and postmarketing clinical experience. It is not recommended to initiate ACTEMRA treatment in patients with a low neutrophil count i.e., absolute neutrophil count (ANC) $<2000/mm^3$. In patients who develop an absolute neutrophil count $<500/mm^3$ treatment is not recommended. Neutrophils should be monitored every 4 to 8 weeks.

Platelets

Treatment with ACTEMRA was associated with a reduction in platelet counts. Treatment-related reduction in platelets was not associated with serious bleeding events in clinical trials. It is not recommended to initiate ACTEMRA treatment in patients with a platelet count below $100{,}000/mm^3$. In patients who develop a platelet count $<50{,}000/mm^3$ treatment is not recommended. Platelets should be monitored every 4 to 8 weeks.

Liver Function Tests
Treatment with ACTEMRA was associated with a higher incidence of transaminase elevations. These elevations did not result in apparent permanent or clinically evident hepatic injury in clinical trials. Increased frequency and magnitude of these elevations was observed when potentially hepatotoxic drugs (e.g., MTX) were used in combination with ACTEMRA. In one case, a patient who had received ACTEMRA 8 mg/kg monotherapy without elevations in transaminases experienced elevation in AST to above 10x ULN and elevation in ALT to above 16x ULN when MTX was initiated in combination with ACTEMRA. Transaminases normalized when both treatments were held, but elevations recurred when MTX and ACTEMRA were restarted at lower doses. Elevations resolved when MTX and ACTEMRA were discontinued. It is not recommended to initiate ACTEMRA treatment in patients with elevated transaminases ALT or AST > 1.5x ULN. In patients who develop elevated ALT or AST > 5x ULN treatment is not recommended. ALT and AST levels should be monitored every 4 to 8 weeks. When clinically indicated, other liver function tests such as bilirubin should be considered.

Lipids
Treatment with ACTEMRA was associated with increases in lipid parameters such as total cholesterol, triglycerides, LDL cholesterol, and/or HDL cholesterol. Assessment of lipid parameters should be performed approximately 4 to 8 weeks following initiation of ACTEMRA therapy, then at approximately 6 month intervals. Patients should be managed according to clinical guidelines [e.g., National Cholesterol Educational Program (NCEP)] for the management of hyperlipidemia.

Immunosuppression
The impact of treatment with ACTEMRA on the development of malignancies is not known but malignancies were observed in clinical studies. ACTEMRA is an immunosuppressant, and treatment with immunosuppressants may result in an increased risk of malignancies.

Hypersensitivity Reactions
Serious hypersensitivity reactions, including anaphylaxis, have been reported in association with infusion of ACTEMRA. Appropriate medical treatment should be available for immediate use in the event of an anaphylactic reaction during administration of ACTEMRA.

Demyelinating Disorders
The impact of treatment with ACTEMRA on demyelinating disorders is not known, but multiple sclerosis and chronic inflammatory demyelinating polyneuropathy were reported rarely in clinical studies. Patients should be closely monitored for signs and symptoms potentially indicative of demyelinating disorders. Prescribers should exercise caution in considering the use of ACTEMRA in patients with preexisting or recent onset demyelinating disorders.

Active Hepatic Disease and Hepatic Impairment
Treatment with ACTEMRA is not recommended in patients with active hepatic disease or hepatic impairment.

Vaccinations
Live vaccines should not be given concurrently with ACTEMRA as clinical safety has not been established. No data are available on the secondary transmission of infection from persons receiving live vaccines to patients receiving ACTEMRA. No data are available on the effectiveness of vaccination in patients receiving ACTEMRA. Because IL-6 inhibition may interfere with the normal immune response to new antigens, patients should be brought up to date on all recommended vaccinations, except for live vaccines, prior to initiation of therapy with ACTEMRA.

8.2.3 Pharmacology – Handling, Storage, Dispensing and Disposal

Tocilizumab will be used per COH institutional SOPs (https://cityofhope.my.salesforce.com/069d0000001W24A) especially in the management of severe CRS when (C-Reactive Protein) CRP is >/=20mg/dl, Marco Davila et al, Science Translational Medicine 2014.[72]

Due to the 1-2 day turn-around time reasonably expected for cytokine results, it is reasonable to consider administration of Tocilizumab based on clinical evidence of CRS, and before having the cytokine data in hand.

9.0 Correlative/Special Studies

9.1 Summary of Correlative Studies

Correlative studies will include collaborative efforts (not limited to) with Translational Genomics Research Institute (TGen),Dr. Swanson's Laboratory at Mayo Clinic, and Dr. Roy's Laboratory at Georgia Tech. Tumor specimen samples (either solid or residual fluid), CSF samples (when available) as well as peripheral blood will be collected from research participants at the designated time points defined in **Section 10,** Study Calendar. Samples will be delivered, processed, stored, and evaluated in accordance with established laboratory practices at COH. Correlative studies performed will provide information regarding the ability of CAR+ T cells to traffic through brain parenchyma and mediate antitumor responses and/or changes in disease status at the site of infusion as well as sites away from catheter placement. These correlative studies will be assessed individually, and collectively, along with RANO criteria and CTCAE grading to provide a fuller understanding of observed toxicities and/or changes in disease/tumor status. Correlatives studies to be performed, but not limited to, are as follows:

9.1.1 Antitumor Responses Following Adoptive Transfer of IL13(EQ)BBζ/CD19t+ T Cells.

MRI/MRS and/or PET assessments will be used to evaluate changes in brain-inflammation, tumor size (e.g., maximal diameter and second perpendicular measurement), and tumor FDG-uptake. Progression free survival at 6 months, and disease response by RANO criteria will be evaluated and compared to historical survival rates to provide evidence of antitumor responses. Additional areas of evaluation will be changes in tumor growth/necrosis near the site of catheter placement versus more distal sites of disease progression. Our ability to measure necrotic tumor volume for these studies is supported by the data presented in **Figure 2.5** above. We will also use mathematical modeling of image-based signatures (MRI) to assess the correlation between tumor growth phenotype and response to therapy on a patient-specific basis and as compared to a virtual control dataset.

9.1.2 Determination of Frequency of IL13(EQ)BBζ/CD19t+ T Cells in the Tumor Microenvironment and Peripheral Blood.

The magnitude and duration of persistence of adoptively transferred IL13(EQ)BBζ/CD19t+ T Cells and their accumulation in tumor specimen samples (either solid or residual fluid) CSF samples (when available) and within the peripheral blood (circulation) will be quantified by Q-PCR with primers specific for the lentiviral WPRE (woodchuck hepatitis virus post-transcriptional regulatory element) sequence, and normalized to cell numbers to read out frequency of gene marked cells in mononuclear cell preps. IL13(EQ)BBζ/CD19t+ T Cells (stained for CD19t, CD3, CD4, CD8) will be also be monitored by flow cytometry and immunohistochemistry. Magnitude and duration of CAR T cell persistence will be evaluated with respect to observed toxicities and/or changes in disease/tumor status (referencing the correlatives within **Section 9**).

9.1.3 *Ex vivo* Immunophenotyping/Functional Analysis

Direct *ex vivo* analyses of persisting infused cells will be performed directly on tumor aspirates and tumor resection material (when available), CSF samples (when available) as well as peripheral blood, using multiparameter flow cytometric analyses, immunohistochemistry, gene expression analysis, and

metabolomic/lipidomic profiles (i.e., qPCR, bulk and single-cell RNA-seq, TCR clonotyping analysis, mass spectrometry).We will analyze markers of CAR+ T cell activation (including but not limited to CD25, CD69, CD71, CD137, CD107a, Granzyme B) and exhaustion (including but not limited to PD-1, TIM-3, LAG-3). We will also analyze CAR+T cell metabolic state. Further, IL13(EQ)BBζ/CD19t+ T Cells adoptive transfer has the potential to alter the immune-environment of the tumor, and therefore we will also evaluate endogenous immune infiltrates in tumor resection material (when available) CSF samples (when available) and peripheral blood, including endogenous CD4+ and CD8+ T cells, Tregs (CD4/FoxP3+), and tumor associated macrophages (CD14, CD16, HLA-DR). These analyses will be evaluated with respect to observed toxicities and/or changes in disease/tumor status (referencing additional correlatives within **Section 9**) to the infused CAR T cells.

These and other analyses will also be performed on manufactured CAR+ T cell product that will not be infused (i.e., "extra" product, defined as product that cannot be used clinically; most commonly, this will be because patients have withdrawn from the trial), to facilitate comparisons between patients and groups of patients. In those cases, patient product may be cultured and/stimulated in vitro or in in vivo animal systems before comparison.

Additionally, for participants who continue to receive CAR T cells post progression where additional therapies, such as (but not limited to) bevacizumab, nivolumab, pembrolizumab, and dexamethasone, are allowed the concentration of these agents may be assessed in the CSF and or tumor fluid to evaluate the impact on CAR T cells and endogenous immune responses. Additional markers may be added depending on the post progression therapy(ies) administered to participants.

9.1.4 Serum, CSF, and Tumor Cavity Cytokine Analysis

Tumor cavity aspirates, CSF samples (when available), and serum from peripheral blood draws will be analyzed for the presence of cytokines such as GM-CSF, IFN-γ, IL-2, IL-6, IL-10, IL-12, TNF-α, and VEGF, using the Human Cytokine 30-Plex assay (Life Technologies) which is standard in the field of ACT.[54,55] Systemic Th1/Th2 cytokine imbalance has been observed in GBM patients – i.e., decreased circulating IL-12 (Th1) and increased circulating IL-10.[73] Because this favoring of Th2 responses is associated with immunosuppression and poor prognosis for a variety of cancers, [74] we will be interested in seeing if administration of our optimized IL13Rα2-specific CAR+ T cells alters this Th1/Th2 balance in any way. We will also test the level of these cytokines released by the corresponding final cell product after *in vitro* antigen stimulation. In addition, because cytokine levels provide a measure of T-cell functional activity that corresponds to associated clinical toxicity,[55,75] these cytokine analyses will assist us in potentially attributing toxicities and/or changes in disease/tumor status (referencing additional correlatives within **Section 9**) to the infused CAR T cells.

9.1.5 Evaluation of Tumor Antigen IL13α2 Expression and Susceptibility to Re-directed T Cells

Available biopsy/resection material collected prior to and/or after CAR T cell therapy will be used for PCR analysis as well as immunohistochemical analysis of IL13Rα2 as well as other tumor antigens (e.g., HER2, EGFRvIII, GD2, and tumor neoantigens). These samples will also be used for analysis of the presence and location of IL13(EQ)BBζ/CD19t+ T Cells by either immunohistochemistry, imagine mass cytometry, or flow cytometry for more extensive phenotyping. Tumor antigen IL13Rα2 expression levels will be evaluated with respect to observed toxicities and/or antitumor response to the infused CAR T cells.

9.1.6 Evaluation of human anti-CAR antibodies (HACA)

Serum samples will be evaluated for immune responses against our therapeutic agent due to the presence of unique junction-site sequences in the IL13(EQ)BBζ CAR. Cryopreserved or fresh serum samples will be measured for human anti-CAR antibodies (HACA) using a flow cytometry based antibody binding assay.

9.1.7 Evaluation of changes in frequency and phenotype of endogenous immune cell populations and tumor microenvironment

Host immune subsets and alterations in the tumor microenvironment will be assessed by multiparameter flow cytometry, immunohistochemistry, gene expression profiling, and metabolomic/lipidomic analysis (e.g., qPCR, RNA-seq, mass spectrometry) on tumor cavity fluid (TCF), cerebrospinal fluid (CSF), peripheral blood, and tumor resection material, when available. We will evaluate changes in the expression of T cell inhibitory/exhaustion markers (such as PD-1, 2B4, TIM-3, LAG-3, BTLA), activation markers (such as 4-1BB, CD4 and CD8 subsets, CD40L, CD69, OX40 and intracellular level of IFNɣ) as well effector memory T cells ($CD3^{+}CD45RO^{+}CD27^{+}CD62L^{-}$), naïve T cells ($CD3^{+}CD45RA^{+}CD127^{+}$ $CD27^{+}CD62L^{+}$), NK cells ($CD56^{+}CD3^{-}$) and regulatory T cells ($CD4^{+}$, $CD25^{High}$, FOXP3, $CD127^{Low}$), both locally (CSF and TCF) and systemically (PB) pre- and post-CAR T cell therapy. Additionally, we will assess changes in the frequency and activation of myeloid subsets such as granulocytes/neutrophils (CD11b, CD15) and macrophages (CD14, $CD86^{+}$, HLA-DR^{+}), with intracellular measures of immunosuppressive phenotypes such as p-Stat3, Arg, IL-10 and TGFβ. For participants who demonstrate encouraging response to therapy, endogenous immune responses (e.g. circulating tumor-specific T cells) will be further assessed. T cells from PB and/or CSF of participants' pre- and post-T cells infusion will be sorted for TCR deep sequencing and in vitro expansion and autologous tumor reactivity measurements as described previously. PB serum, CSF and TCF will also be evaluated for metabolomic/lipidomic changes by mass spectrometry to reveal treatment related changes in the tumor microenvironment. Additionally, the plan is to perform gene expression profiling at single-cell resolution to evaluate on changes in host immune responses following CAR T cell therapy. These analyses will be evaluated with respect to observed toxicities and/or antitumor response to the infused CAR T cells.

10.0 Study Calendar

10.1 Enrollment and Treatment Study Calendar:

						CAR T Cell Infusions									Post CAR T cell infusion assessments[7]	Optional CAR T Cell Infusions			Post CAR T cell infusion assessments[7]
						Cycle 1			Cycle 2			Cycle 3							
	Pre-Study/Screening	PBMC Collection	Prior to Surgical Procedure[5]	Surgical Procedure / Rickham Placement[5]	Post Surgery Evaluations[5]	Prior to CAR T cell Infusion/Enrollment	Infusion	Interim evaluations [6]	Prior to CAR T cell Infusion	Infusion	Interim evaluations[6]	Prior to CAR T cell Infusion	Infusion	Interim evaluations[6]		Prior to CAR T cell Infusion	Infusion	Interim evaluations[6]	
Informed Consent	X																		
Demographics	X																		
History and Physical	X					X		X	X		X	X		X		X		X	
Concurrent Meds	X							X			X			X				X	
Vital Signs, KPS			X			X	X	X	X	X	X	X	X	X	X	X	X	X	X
Quality of Life Questionnaires			X[5]			X			X			X			X	X[11]			X[11]
CBC, Differential	X		X			X		X	X		X	X		X	X	X		X	X
Comprehensive Metabolic Panel	X		X			X		X	X		X	X		X	X	X		X	X
CRP, Ferritin[1]						X		X	X		X	X		X	X	X		X	X
Serum Pregnancy Test	X																		
QuantiFERON-TB Gold or equivalent	X																		
HIV Ag/Ab Combo Assay	X																		

		Pre-Study/Screening	PBMC Collection	Prior to Surgical Procedure[5]	Surgical Procedure / Rickham Placement[5]	Post Surgery Evaluations[5]	CAR T Cell Infusions: Cycle 1: Prior to CAR T cell Infusion/Enrollment	Cycle 1: Infusion	Cycle 1: Interim evaluations [6]	Cycle 2: Prior to CAR T cell Infusion	Cycle 2: Infusion	Cycle 2: Interim evaluations[6]	Cycle 3: Prior to CAR T cell Infusion	Cycle 3: Infusion	Cycle 3: Interim evaluations[6]	Post CAR T cell infusion assessments[7]	Optional CAR T Cell Infusions: Prior to CAR T cell Infusion	Optional: Infusion	Optional: Interim evaluations[6]	Post CAR T cell infusion assessments[7]
IHC Target Confirmation		X																		
Tumor Biopsy/Resection[3]					X[5]															
FDG-PET						X[5,9]										X[13]				X[12]
MRI +/- contrast						X[5,9]										X				X[12]
Adverse Event Evaluation[4]							------------------------ X ------------------------													
Apheresis			X																	
Correlative Studies	Tumor Cavity Aspirate/CSF[3]							X[15]	X[10, 14]		X[14]	X[10, 14]		X[14]	X[10, 14]			X[14]	X[10, 14]	
Correlative Studies	5 cc Peripheral Blood Draw	X																		
Correlative Studies	35 cc Peripheral Blood Draw			X[5,8]			X		X	X		X	X		X		X	X[15]	X	
Drug	Study Agent (CAR T cells)							X			X			X				X		

1 Daily levels should be taken if a patient experiences persistent fevers (38^{0}C) and clinical indications of toxicity
2 Positive results may require additional testing, and expedited FDA reporting.
3 When feasible, tumor tissue, tumor cavity aspirate, or CSF samples may be collected for correlative studies during clinically-indicated and/or standard of care procedures.
4 AE evaluations to begin on day of first CAR T cell infusion

5 For the optional infusions, evaluations are NOT required to be repeated for Rickham catheter placements that take place after completing the first three required CAR T cell infusions; may be conducted at the discretion of the PI/designee. Following catheter placement, the research participant will be followed per the 'optional cycles' calendar in **Section 10.1**.
6 Interim evaluations, specifically correlative blood draws, will be done at least twice a week
7 Assessments to occur no sooner than one week post last CAR T cell infusion
8 Correlative samples may be drawn at time of pre-operative work-up
9 Imaging required 1-2 weeks prior to the start of the first CAR T cell infusion
10 When feasible, tumor cavity aspirate/CSF samples to be drawn, per PI/Designee discretion, in the event of toxicity management and/or interim evaluations
11 QoL questionnaires MAY be administered but are not required
12 For the optional infusions, imaging may be conducted per PI/Designee discretion
13 Imaging required within 2 weeks from the third CAR T cell infusion
14 At the discretion of the PI/designee, the following tests may be ordered (culture, cytology, cell count, glucose and/or protein)
15 At the discretion of the PI/designee, additional correlative blood draws may be collected prior to the CAR T cell infusion.

10.2 Short-Term Follow-Up Calendar

		Post Final CAR T Cell Infusion					
		Follow-Up Week +4 (+/- 3 days)	Month 3 (+/- 4 weeks)	Month 6 (+/- 4 weeks)	Month 8 (+/- 4 weeks)	Month 10 (+/- 4 weeks)	Month 12 (+/- 4 weeks)
H&P			X	X	X	X	X
Vital Signs, KPS		X	X	X	X	X	X
Quality of Life Questionnaires		X					
CBC, Differential, Platelet Count		X	X	X	X	X	X
Comprehensive Metabolic Panel		X	X	X	X	X	X
CRP, Ferritin							
HIV qPCR testing[1]			X	X			X
Tumor Biopsy/CSF[2]							
FDG-PET		----------------------- X[3] -----------------------					
MRI +/- Contrast		----------------------- X[3] -----------------------					
Adverse Event Evaluation		----------------------- X -----------------------					
Correlative Studies	35 cc Peripheral Blood	----------------------- X[4] -----------------------					

1 Reference **Section 5.6.2** (Long-Term Follow-Up). Testing may be done either by Ag/Ab combo assay or qPCR. Positive results may require additional testing, and expedited FDA reporting
2 When feasible, to be collected at any time during clinically-indicated interventions or as part of patient's standard of care management.
3 FDG-PET & MRI +/- contrast are not required at the designated time-points but may be ordered per SOC timeline/PI or designee discretion
4 Patients who do not have a local lab/oncologist office to assist with mailing kits to COH do not require correlative blood draw to be collected UNLESS they are reported to have a suspected AE theoretically related CAR T (per the list in **Section 10.3**).

10.3 Long-Term Follow-Up (or Modified Follow-Up for Participants that Progress):

		Post Final CAR T Cell Infusion				
		Day 30 (+ 7 days)	Month 3 (+/- 4 weeks)	Month 6 (+/- 4 weeks)	Month 12 (+/- 4 weeks)	LTFU Evaluation: Yearly post final CAR T cells[2]
H&P, Vital Signs, KPS		X	X	X	X	
Assessment for Reproductive Risk and Risk to Fetus		X	X	X	X	X
Assessment for Autoimmune Toxicities		X	X	X	X	X
CBC, Differential, Platelet Count		X	X	X	X	
Comprehensive Metabolic Panel		X	X	X	X	
CRP, Ferritin		X				
HIV qPCR testing[1]			X	X	X	X
Concurrent Meds		----------X--------				
Correlative Studies	35 cc Peripheral Blood	----------X[3]--------				
Adverse Event Evaluation		----------X--------				

1 Reference **Section 5.6.2** (Long-Term Follow-Up). Testing may be done either by Ag/Ab combo assay or qPCR. Positive results may require additional testing, and expedited FDA reporting
2 Research participants will be followed and data will be collected on IRB#13384 per FDA guidelines until a separate long term follow up protocol is executed.
3 Patients who do not have a local lab/oncologist office to assist with mailing kits to COH do not require correlative blood draw to be collected UNLESS they are reported to have a suspected AE theoretically related CAR T.

11.0 Endpoint Evaluation Criteria/Measurement of Effect

11.1 Response Criteria

Endpoint Evaluation: Endpoint Evaluation is listed in **Section 13.3**.

Response Criteria: Toxicity and adverse event will be assessed using CTCAE v4.0 and the revised CRS grading system (**Section Appendix 16.3** from data obtained at each clinical assessment as outlined in **Section 10**. Symptoms and toxicities will be evaluated as follows:

- Physical exam and Blood Chemistry/Hematology results
- Adverse event reporting

Additionally, disease status will be assessed by the grading of the tumor responses performed according to the RANO criteria, reference **Appendix 16.2**.

12.0 Data Reporting/Protocol Deviations

12.1 Data Reporting

12.1.1 Confidentiality and Storage of Records

The original data collection forms will be sent to COH Clinical Trials Office (CTO) assigned Clinical Research Coordinator (CRC) and stored in a locked cabinet in within the assigned locked office of T Cell Therapeutics Research Laboratory (TCTRL) Clinical and Regulatory Support staff. This protocol also uses Electronic Data Collection where data is stored in encrypted, password protected, secure computers that meet all HIPAA requirements. When results of this study are reported in medical journals or at meetings, identification of those taking part will not be disclosed. Medical records of subjects will be securely maintained in the strictest confidence, according to current legal requirements. They will be made available for review, as required by the FDA, HHS, or other authorized users such as the NCI, under the guidelines established by the Federal Privacy Act and rules for the protection of human subjects.

12.1.2 Subject Consent Form

At the time of registration, the original signed and dated Informed Consent form, HIPAA research authorization form, and the California Experimental Subject's Bill of Rights (for the medical record) and three copies (for the subject, the research record, and the Coordinating Center) must be available. All Institutional, NCI, Federal, and State of California requirements will be fulfilled.

12.1.3 Data Collection Forms and Submission Schedule

All data will be collected using study designed electronic Case Report Forms (eCRFs), as well as Administrative Forms which are maintained by the TCTRL Clinical and Regulatory Support Staff. Data will be captured to the best of our ability within 10 working days of the event(s) being monitored. Data will be sent to the location identified in **Section 12.1.1** and stored in a secure location.

12.1.3.1Eligibility Checklist

The Eligibility Checklist must be completed by a protocol nurse or clinical research associate and signed by an authorized investigator prior to registering the subject. See **Section 4.3** for the registration procedure.

12.1.3.2Prior Therapy Forms and On-Study Forms

Within 30 days of registration, the clinical research associate will submit study enrollment and baseline form as described in **Section 12.1.1.**

12.2 Protocol Deviations

12.2.1 Deviation Policy

This protocol will be conducted in accordance with COH's "Clinical Research Protocol Deviation Policy" located at http://www.coh.org/dsmc/Documents/Institutional%20Deviation%20Policy.pdf.

Deviations from the written protocol that could increase patient risk or alter protocol integrity require prior IRB approval of a single subject exception (SSE) request. In addition, if contractually obligated, the sponsor must also approve the deviation. IRB pre-approved SSE protocol modifications are considered an amendment to the protocol and not a deviation. The submission of a deviation report is not required.

Brief interruptions and delays may occasionally be required due to travel delays, airport closure, inclement weather, family responsibilities, security alerts, government holidays, etc. This can also extend to complications of disease or unrelated medical illnesses not related to disease progression. The PI has the discretion to deviate from the protocol when necessary so long as such deviation does not threaten patient safety or protocol scientific integrity. Examples include, but are not limited to: a) dose adjustments based on excessive patient weight; b) alteration in treatment schedule due to non-availability of the research participant for treatment; c) laboratory test results which are slightly outside the protocol requirements but at levels that do not affect participant safety. These instances are considered to be deviations from the protocol. A deviation report will be submitted to the DSMC/IRB within five days.

12.2.2 Reporting of Deviations

All deviations will be reported to the COH DSMC within five days. The DSMC will forward to report to the IRB following review.

12.2.3 Resolving Disputes

The COH Investigational Drug Service (IDS) cannot release a research agent that would cause a protocol deviation without approval by the PI. Whenever the protocol is ambiguous on a key point, the IDS should rely on the PI to clarify the issue.

In situations where there is misperception or dispute regarding a protocol deviation among the persons involved in implementing the protocol, it is the responsibility of the PI to resolve the dispute and the PI may consult with the DSMC chair (or designee) to arrive at resolution.

13.0 Statistical Considerations

13.1 Study Design

This protocol is a Phase I feasibility/safety study of IL13(EQ)BBζ/CD19t+ T cells which implements both inter- and intra-patient dose escalation in five patient arms (T_{CM} intratumoral, intracavity, intraventricular, and dual delivery; as well as $T_{N/MEM}$ dual delivery) in recurrent/refractory malignant glioma.

Table 13.1: Dose Schedule (CAR+) for Arms 1 to 5^

Planned Cycle	Dose schedule 1de	Dose schedule 1	Dose schedule 2de	Dose schedule 2	Dose Schedule 3de	Dose schedule 3
1	A: $2x10^6$	A: $2x10^6$	C: $10x10^6$	C: $10x10^6$	C:$10x10^6$	F: $20x10^6$
2	B: $5x10^6$	C: $10x10^6$	D: $25x10^6$	E: $50x10^6$	G:$75x10^6$	H:$100x10^6$
3	B: $5x10^6$	C: $10x10^6$	D: $25x10^6$	E: $50x10^6$	G:$75x10^6$	H:$100x10^6$

Total Dose#	*12x10⁶*	*22x10⁶*	*60x10⁶*	*110x10⁶*	*160x10⁶*	*220x10⁶*
	Evaluation/Restaging					
Optional Cycles#	≤5x10⁶	≤10x10⁶	≤25x10⁶	≤50x10⁶	≤75x10⁶	≤100x10⁶

^ Infusion doses should not exceed $2.5x10^8$ per 0.5mL ($0.5x10^6$/µL); Reference **Section 5.2.1**.
de = de-escalation schedule
# The dose listed at each cycle is the CAR+ T cell dose intended for each delivery method, therefore in Arms 4 (T_{CM} Dual Delivery) and 5 ($T_{N/MEM}$ Dual Delivery) 2 infusions doses will be prepared per infusion cycle, 1 for each delivery method.

13.1.1 Study Participant Staggering Rules

The first 4 research participants in Arms 1 through 3 will be treated sequentially, followed through the DLT period (3 infusions cycles plus 1-week for AE evaluations) before the next participant may receive their initial infusion; all further research participants will be treated in cohorts of 3. The first research participant in Arms 4 and 5 will be followed through the DLT period before the other 2 participants in that cohort are treated, all further participants will be followed in cohorts of 3. Dose escalation rules for intra-patient and inter-patient scheduling are provided in **Section 13.2**. Study Stopping Rules are provided in **Section 13.4**.

13.2 Dose Escalation Rules (each arm will be followed separately):

Note that the dose escalation plan calls for intra-patient escalation through dose levels labeled with letters in **Table 13.1**, as well as inter-cohort dose escalation through schedules 1, 2 and 3 in the columns of **Table 13.1**. Schedules 1de, 2de and 3de describe possible de-escalation schedules (shown in grey).

13.2.1 Intra-patient dose escalation rules:

We implement intra-patient dose escalation starting with an initial low dose of CAR T cells to minimize the extent of unexpected on-tumor and off-tumor targeting toxicities, with the goal of determining a final dosing regimen of 3 weekly infusions with a DLT rate less than 33%. The intra-patient dose escalation/de-escalation rules are based on toxicities with an attribution of probable or definitely related to CAR T cell infusion with the exception of those listed in **Section 8.1** of the protocol will be the following (**Table 13.2**).

Table 13.2: Rules for CAR T cell dose on day of the infusion	
**On the day of scheduled infusion, if an attributed toxicity from the previous infusion(s) is probably or definitely related to CAR T cells AND is:**	**Then:**
Toxicities listed in **Section 8.1**	These toxicities are considered manageable but would result in cancellation of the cell dose for that day. The next scheduled dose will restart at the planned dose level.
Grade 2 CRS, and Grade 3 and above toxicities	Defined as a medically significant but not immediately life threatening toxicity will result in cancellation of cell infusion for that day. The next scheduled infusion will re-start at the same dose level. Before the study participant can escalate to the next dose level, they must receive the assigned cell infusion dose without experiencing a grade 3 (grade 2 CRS) or higher toxicity not listed in section 8.1.
	If on re-start the study participant has a second grade 3 toxicity (or grade 2 CRS) at the same dose this will be considered a DLT. At the discretion of the PI the participant can either be taken off treatment or be de-escalated to the next lower dose for the next cycle. If there is no lower dose the study participant will be taken off treatment.
**If maximum attributed toxicity from the previous infusion(s) is probably or definitely related to CAR T cells AND is:**	**Then:**
DLTs toxicities listed in **Section 5.7b through e**	Will be defined as a DLT and the study participant will be taken off treatment.
Any attributed toxicity that causes a 2 week delay in dosing	Will be defined as a DLT and the study participant will be taken off treatment.

13.2.2 Inter-cohort dose escalation rules

Inter-cohort dose escalation rules for escalating and de-escalating through the schedules follow a 3+3 design with de-escalation in half step as described below.

1) The first cohort of 3 participants will receive **dose schedule 1** with the option of receiving additional cycles of treatment at ≤ the individual participant's maximum achieved safe dose after a minimum of 1 week break (**Tables 13.1**). Participants will be followed for DLTs from start of study until 1 week after the cycle 3 or the last infusion cycle in the DLT period.
2) If all participants from a cohort complete a schedule without a DLT, the next cohort will escalate to the next schedule, moving through schedules 1, 2 and 3.
3) If 2 of 3 participants cannot complete dose level A (i.e., the low dose) without experiencing a DLT we will put the study on hold to determine if a lower dose should be considered.
4) If 2 of 3 participants in a cohort do not complete a schedule (i.e., all 3 cycles) without experiencing a DLT, we will de-escalate to the next lower schedule in **Tables 13.1.**

Schedule 1→Schedule 1de: 1 cycles of dose A followed by 2 cycles of dose B
Schedule 2→Schedule 2de: 1 cycle of dose C followed by 2 cycles of dose D
Schedule 3→Schedule 3de: 1 cycle of dose C followed by 2 cycles of dose G

5) If 1 of 3 participants in a cohort does not complete a schedule without experiencing a DLT, a second cohort of 3 participants will repeat that schedule.
   i. If 5 of the 6 participants from the combined cohorts complete the schedule without experiencing a DLT, the next cohort of 3 participants will escalate to the next schedule.
   ii. If 4 or fewer of the 6 participants completes the current schedule without experiencing a DLT we will de-escalate to next lower schedule in **Tables 13.1**.
6) The MTD/MFD schedule will be the highest dose schedule in which fewer than 33% of participants experience a DLT, when at least 6 participants were treated and are evaluable for toxicity.
7) An expansion cohort of 6 participants will be added at the MTD/MFD dose schedule.
8) The RP2D will be determined based on the maximum tolerated dose schedule/maximum feasible dose schedule, the results from the expansion cohort at the MTD/MFD schedule in combination with the number of additional weeks participants were able to stay on that dose and the activity data.

Tables 13.3 to 13.5 provide operating characteristics for Arms 1 to 5 based on 1000 simulated trials for each set of toxicity schedules. The operating characteristics include the rate each schedule was chosen as the MTD/MFD, and the mean DLT rate at the MTD/MFD and the median trial sample size. Note that trial seeks the highest schedule with a DLT rate < 33% (≤17%).

Table 13.3 Sample Size: Median=15	Toxicity Rates			MTD/MFD rate
Toxicity levels/ Dose Schedules	Normal to Moderate	Severe*	Life Threatening*	
Mean DLT rate at the MTD/MFD (std.dev)				0.098 (.082)
All doses are too toxic				<0.01
Schedule 1de				0.10
Cell Dose 1 –A	0.98	0.01	0.01	
Cell Dose 2 –B	0.95	0.025	0.025	
Cell Dose 3 –B	0.95	0.025	0.025	
Schedule 1				0.035
Cell Dose 1 –A	0.98	0.01	0.01	
Cell Dose 2 –C	0.875	0.075	0.05	
Cell Dose 3 –C	0.875	0.075	0.05	
Schedule 2de				0.16
Cell Dose 1 –C	0.875	0.075	0.05	
Cell Dose 2 –D	0.85	0.10	0.05	
Cell Dose 3 –D	0.85	0.10	0.05	
Schedule 2				0.17
Cell Dose 1 –C	0.875	0.075	0.05	
Cell Dose 2 –E	0.80	0.15	0.05	
Cell Dose 3 –E	0.80	0.15	0.05	
Schedule 3de				0.19
Cell Dose 1 –C	0.875	0.075	0.05	
Cell Dose 2 –G	0.75	0.15	0.01	
Cell Dose 3 –G	0.75	0.15	0.1	
Schedule 3				0.33
Cell Dose 1 –F	0.85	0.1	0.05	
Cell Dose 2 –H	0.70	0.2	0.1	
Cell Dose 3 –H	0.70	0.2	0.1	

*The toxicity levels severe and life threatening are defined in **Table 13.2**.

**Table 13.4 Sample Size: Median=15**	**Toxicity Rates**			**MTD/MFD rate**
Toxicity levels/ Dose Schedules	Normal to Moderate	Severe*	Life Threatening*	
Mean DLT rate at the MTD/MFD (std.dev)				0.099 (.082)
All doses are too toxic				<0.01
Schedule 1de				0.11
Cell Dose 1 –A	0.98	0.01	0.01	
Cell Dose 2 –B	0.95	0.025	0.025	
Cell Dose 3 –B	0.95	0.025	0.025	
Schedule 1				0.20
Cell Dose 1 –A	0.98	0.01	0.01	
Cell Dose 2 –C	0.875	0.075	0.05	
Cell Dose 3 –C	0.875	0.075	0.05	
Schedule 2de				0.23
Cell Dose 1 –C	0.875	0.075	0.05	
Cell Dose 2 –D	0.80	0.10	0.1	
Cell Dose 3 –D	0.80	0.10	0.1	
Schedule 2				0.16
Cell Dose 1 –C	0.875	0.075	0.05	
Cell Dose 2 –E	0.70	0.2	0.1	
Cell Dose 3 –E	0.70	0.2	0.1	
Schedule 3de				0.17
Cell Dose 1 –C	0.875	0.075	0.05	
Cell Dose 2 –G	0.65	0.25	0.1	
Cell Dose 3 –G	0.65	0.25	0.1	
Schedule 3				0.13
Cell Dose 1 –F	0.85	0.1	0.05	
Cell Dose 2 –H	0.60	0.25	0.15	
Cell Dose 3 –H	0.60	0.25	0.15	

*The toxicity levels severe and life threatening are defined in **Table 13.2**.

Table 13.5 Sample Size: Median=15	Toxicity Rates			MTD/MFD rate
Toxicity levels/ Dose Schedules	Normal to Moderate	Severe*	Life Threatening*	
Mean DLT rate at the MTD/MFD (std.dev)				0.094 (.083)
All doses are too toxic				<0.01
Schedule 1de				0.12
Cell Dose 1 –A	0.98	0.01	0.01	
Cell Dose 2 –B	0.95	0.025	0.025	
Cell Dose 3 –B	0.95	0.025	0.025	
Schedule 1				0.35
Cell Dose 1 –A	0.98	0.01	0.01	
Cell Dose 2 –C	0.875	0.075	0.05	
Cell Dose 3 –C	0.875	0.075	0.05	
Schedule 2de				0.39
Cell Dose 1 –C	0.875	0.075	0.05	
Cell Dose 2 –D	0.75	0.15	0.10	
Cell Dose 3 –D	0.75	0.15	0.10	
Schedule 2				0.089
Cell Dose 1 –C	0.875	0.075	0.05	
Cell Dose 2 –E	0.50	0.30	0.2	
Cell Dose 3 –E	0.50	0.30	0.2	
Schedule 3de				0.023
Cell Dose 1 –C	0.875	0.075	0.05	
Cell Dose 2 –G	0.50	0.30	0.20	
Cell Dose 3 –G	0.50	0.30	0.20	
Schedule 3				0.021
Cell Dose 1 –F	0.75	0.15	0.10	
Cell Dose 2 –H	0.80	0.30	0.05	
Cell Dose 3 –H	0.80	0.30	0.05	

13.3 Optional additional cycles

Participants can continue receiving CAR T cell infusions at a rate where infusions are no more frequent than once a week. Additional cycles if administered through the same route of delivery as the first three cycles, will be administered at ≤ the highest tolerated cell dose in the initial dose schedule, provided that the participant continues to meet eligibility criteria and there are cell doses available from the already manufactured cell product. If a research participant on the intracranial administration arms (intracavitary or intratumoral) progresses after the first three CAR T cell infusion cycles, at the discretion of the PI, the research participant may move to intraventricular administration for the optional CAR T cell infusions. For research participants in Arms 4 and 5, based on clinical response after the first three infusions, the study PI may decide to continue with the optional infusions at either one or both sites (instead of requiring injections at both sites) ; these infusion doses may be delivered at up to the highest dose deemed safe for that delivery site..

13.4 Study Endpoints:

- Primary: Grade 3 toxicities, DLTs, and all other toxicities.
- Secondary:
  - Disease response by RANO criteria from initial infusion method;
  - Overall survival from time of surgery to death date or last contact date;
  - Functioning scale, symptom scale, and item scores from the EORTC QLQ-C30;
  - CAR T cells and cytokine levels in peripheral blood, tumor cavity fluid, and CSF;
  - Endogenous immune cell detection in peripheral blood, tumor cavity fluid, and CSF; and
  - Tumor/tumor micro-environment marker detection.
- Exploratory:
  - Timing and extent of brain inflammation following CAR T cell administration;
  - CAR T cell product characteristics;
  - Persistence and location of CAR T cells in tumor micro-environment; and
  - IL13Rα2 antigen expression levels.

13.5 Study Stopping Rules

13.5.1 Rules for Holding Accrual Due to Toxicity

The study will hold accrual and the data will be reviewed, if within 30 days of treatment either DLT level toxicities with attribution of probable or definite is observed in greater than 50% of participants out of at least 6 participants, or any grade 3 or higher organ toxicity (cardiac, dermatologic, gastrointestinal, hepatic, pulmonary, renal/genitourinary, or neurologic) not pre-existing or due to the underlying malignancy, or a Grade 5 toxicity is observed with attribution of possibly, probably or definitely.

13.6 Sample Size Accrual Rate

As of 03/02/2018, we have closed Arm 1 (biopsy with an N=2) as biopsy participants can be included in the ICV or dual arms. Based on simulation results we expect to study 15 participants in the dose escalation portion of the trial, plus 6 in the expansion portion plus 2 for replacement of unevaluable participants giving an expected sample size of 23 per arm for each of the 4 open arms for a total of 92. To be evaluable for defining the MTD/MFD a research participant must either complete three infusion cycles receiving at least 80% of each defined infusion dose and at least one rest cycle, or have had a DLT. A sample size of 12 at the MTD/MFD will provide us with *i*) maximum margin of error of 0.25 for a 95% confidence interval (95% CI) for the DLT rate, and *ii*) to detect a toxicity with a true rate of 0.15 in 85% of trials. The full sample size of 21 will provide us with a maximum margin of error 0.19 for a 95% CI for progression free survival at 6 months and disease response. Accrual is expected to be 6 evaluable research participants per year per arm. Thus, we expect to complete accrual in 5 years.

13.7 Statistical Analysis Plan

The statistical analysis will be done separately for each arm. Rate and associated 95% Clopper and Pearson binomial confidence limits (95% CI) will be estimated for participants' experiencing DLTs at the RP2D schedule. In study participants that received the full schedule of 3 CAR T cell doses, we will estimate the rate (95% CI) with disease response, and Kaplan Meier methods will be used to estimate median PFS and OS and graph the results. In study participants that receive at least 1 dose of CAR T cells we will estimate the mean and standard error for change from baseline during treatment and post treatment in the quality of life functioning scale, symptom scale and item scores from the EORTC QLQ-C30. Statistical and graphical methods will be used to describe persistence and expansion of the CAR T cells (peripheral blood, tumor cavity fluid, and CSF) and cytokine levels (CSF, tumor cavity fluid, peripheral blood) over the study period.

In study participants that undergo a second resection or following autopsy, CAR T cell numbers, location, and antigen levels will be described. Tables will be created to summarize all toxicities and side effects by dose, time post treatment, organ, severity and arm. We will provide descriptive statistics for patient demographics.

14.0 Human Subject Issues

14.1 Institutional Review Board

In accordance with City of Hope policies, an Institutional Review Board (IRB) that complies with the federal regulations at 45 CFR 46 and 21 CFR 50, 56 and State of California Health and Safety code, Title 17, must review and approve this protocol and the informed consent form prior to initiation of the study. All institutional, NCI, Federal, and State of California regulations must be fulfilled.

14.2 Recruitment of Subjects

Research participants will be identified through the clinical practices of the PI, co-Is and participating clinicians and through direct referrals from outside hospitals and physicians. No direct-to-patient advertising will be performed without IRB approval.

14.3 Advertisements

Advertisements to include print, media (radio, television, billboards), telephone scripts, lay summary to be posted on City of Hope's public Clinical Trials On-Line^{SM} website, etc., will be reviewed and approved by the IRB prior to their use to recruit potential study subjects.

14.4 Study location and Performance Sites

This study will be performed at COH.

14.5 Confidentiality

This research will be conducted in compliance with federal and state of California requirements relating to protected health information (PHI). The principal investigator, co-investigators, and laboratory technicians will have access to all study information captured in electronic data bases as well as any patient and study data documented in individual patient research binders, but all information will be treated confidentially. No identifiers will be used in any subsequent publication of these results.

14.6 Financial Obligations and Compensation

The investigational drug, autologous IL13(EQ)BBζ/CD19t+ T Cells, will be provided free of charge by City of Hope. Should this drug become commercially available during the course of your treatment, the research participant and/or the insurance carrier may be asked to pay for the costs of the drug.

The standard of care drug(s) or devices and procedures provided will be the responsibility of the research participant and/or the insurance carrier. The research participant will be responsible for all copayments, deductibles, and other costs of treatment and diagnostic procedures as set forth by the insurance carrier. The research participant and/or the insurance carrier will be billed for the costs of treatment and diagnostic procedures in the same way as if the research participant were not in a research study. However, neither the research participant nor the insurance carrier will be responsible for the research procedures related to this study.

In the event of physical injury to a research participant, resulting from research procedures, appropriate medical treatment will be available at the City of Hope to the injured research participant, however, financial compensation will not be available.

The research participant will not be paid for taking part in this study.

14.7 Informed Consent Processes

The Principal Investigator or IRB approved named designate will explain the nature, duration, purpose of the study, potential risks, alternatives and potential benefits, and all other information contained in the informed consent document. In addition, they will review the experimental subject's bill of rights and the HIPAA research authorization form. Research subjects will be informed that they may withdraw from the study at any time and for any reason without prejudice, including as applicable, their current or future care or employment at City of Hope or any relationship they have with City of Hope. Research subjects will be afforded sufficient time to consider whether or not to participate in the research.

Should sufficient doubt be raised regarding the adequacy of comprehension, further clarifications will be made and the questionnaire repeated until a satisfactory result is obtained. Prospective research subjects who cannot adequately comprehend the fundamental aspects of the research study with a reasonable amount of discussion, education and proctoring will be ineligible for enrollment. For those subjects who do comprehend the fundamental aspects of the study, consent will be obtained and documented, followed by eligibility testing. The research team will review the results of eligibility testing and determine if the subject is a candidate for study enrollment.

15.0 References

1. Cartellieri, M., *et al.* Chimeric antigen receptor-engineered T cells for immunotherapy of cancer. *J Biomed Biotechnol* **2010**, 956304 (2010).

2. Brown, C.E., *et al.* Tumor-Derived Chemokine MCP-1/CCL2 Is Sufficient for Mediating Tumor Tropism of Adoptively Transferred T Cells. *J Immunol* **179**, 3332-3341 (2007).

3. Hong, J.J., *et al.* Successful treatment of melanoma brain metastases with adoptive cell therapy. *Clin Cancer Res* **16**, 4892-4898 (2010).

4. Yaghoubi, S.S., *et al.* Noninvasive detection of therapeutic cytolytic T cells with 18F-FHBG PET in a patient with glioma. *Nat Clin Pract Oncol* **6**, 53-58 (2009).

5. Kahlon, K.S., *et al.* Specific recognition and killing of glioblastoma multiforme by interleukin 13-zetakine redirected cytolytic T cells. *Cancer Res* **64**, 9160-9166 (2004).

6. Brown, C.E., *et al.* Stem-like Tumor-Initiating Cells Isolated from IL13Ralpha2 Expressing Gliomas Are Targeted and Killed by IL13-Zetakine-Redirected T Cells. *Clin Cancer Res* **18**, 2199-2209 (2012).

7. Berger, C., *et al.* Adoptive transfer of effector CD8 T cells derived from central memory cells establishes persistent T cell memory in primates. *J Clin Invest* **118**, 294-305 (2008).

8. Wang, X., *et al.* Engraftment of human central memory-derived effector CD8+ T cells in immunodeficient mice. *Blood* **117**, 1888-1898 (2011).

9. Stupp, R., *et al.* Radiotherapy plus concomitant and adjuvant temozolomide for glioblastoma. *N Engl J Med* **352**, 987-996 (2005).

10. Wong, E.T., *et al.* Outcomes and prognostic factors in recurrent glioma patients enrolled onto phase II clinical trials. *J Clin Oncol* **17**, 2572-2578 (1999).

11. Chang, S., Kuhn, J., Wen, P.Y. & al., E. Phase II/pharmacokinetic study of CCI-779 in recurrent GBM. *Neuro Oncol* **5**, 349 [abstract] (2003).

12. Conrad, C., Friedman, H., Reardon, D. & al., E. A phase I/II trial of singl-agen PTK 787/ZK222584 (PTK/ZK), a novel, oral angiogenesis inhibitor, in patients with recurrent glioblastoma multiforme (GBM). *J Clin Oncol* **22**, 1512 [abstract] (2004).

13. Lieberman, F.S., Cloughesy, T., Malin, M. & al., E. Phase I-II study of ZD-1839 for recurrent malignant gliomas and meningiomas progressing after radiation therapy. *J Clin Oncol* **22**(2003).

14. Raizer, J.J., Abrey, L.E., Wen, P. & al., E. A phase II trial or erlotinib (OSI-774) in patients with recurrent malignant gliomas (MG) not on EIAEDs. *J Clin Oncol* **22**, 1502 [abstract] (2004).

15. Rich, J.N., *et al.* Phase II trial of gefitinib in recurrent glioblastoma. *J Clin Oncol* **22**, 133-142 (2004).

16. Van den Bent, M., Brandes, A.A., van Oosterom, A. & al., E. Multicentre phase II study of imatinib esylate (Gleevec) in patients with recurrent glioblastoma. *Neuro Oncol* **6**, 383 [abstract] (2004).

17. Vogelbaum, M.A., Peereboom, G., Stevens, G. & al., E. Phase II trial of the EGFR tyrosine kinase inhibotor erlotinib for signel agen therapy of recurrent glioblastoma multiforme: interim results. *J Clin Oncol* **22**, 1558 [abstract] (2004).

18. Yung, A., Vredenburgh, J., Cloughesy, T. & al., E. Erlotinib HCL for glioblastoma multiforme in first relapse, a phase II trial. *J Clin Oncol* **22**, 1555 [abstract] (2004).

19. Yung, W.K.A., Friedman, H., Jackson, E. & al., E. A phase I trial of PTK787/ZK 222584 (PTK/ZK), a novel oral VEGFR TK inhibitor in recurrent glioma. *Proc Am Soc Clin Oncol*, [abstract 315] (2004).

20. Gorlia, T., *et al.* New prognostic factors and calculators for outcome prediction in patients with recurrent glioblastoma: a pooled analysis of EORTC Brain Tumour Group phase I and II clinical trials. *Eur J Cancer* **48**, 1176-1184 (2012).

21. Fujisawa, T., Joshi, B.H. & Puri, R.K. IL-13 regulates cancer invasion and metastasis through IL-13Ralpha2 via ERK/AP-1 pathway in mouse model of human ovarian cancer. *Int J Cancer* **131**, 344-356 (2012).

22. Hsi, L.C., *et al.* Silencing IL-13Ralpha2 promotes glioblastoma cell death via endogenous signaling. *Mol Cancer Ther* **10**, 1149-1160 (2011).

23. Brown, C.E., *et al.* Glioma IL13Ralpha2 is associated with mesenchymal signature gene expression and poor patient prognosis. *PLoS One* **8**, e77769 (2013).

24. Debinski, W., Gibo, D.M., Hulet, S.W., Connor, J.R. & Gillespie, G.Y. Receptor for interleukin 13 is a marker and therapeutic target for human high-grade gliomas. *Clin Cancer Res* **5**, 985-990 (1999).

25. Jarboe, J.S., Johnson, K.R., Choi, Y., Lonser, R.R. & Park, J.K. Expression of interleukin-13 receptor alpha2 in glioblastoma multiforme: implications for targeted therapies. *Cancer Res* **67**, 7983-7986 (2007).

26. Joshi, B.H., *et al.* Identification of interleukin-13 receptor alpha2 chain overexpression in situ in high-grade diffusely infiltrative pediatric brainstem glioma. *Neuro Oncol* **10**, 265-274 (2008).

27. Kawakami, M., Kawakami, K., Takahashi, S., Abe, M. & Puri, R.K. Analysis of interleukin-13 receptor alpha2 expression in human pediatric brain tumors. *Cancer* **101**, 1036-1042 (2004).

28. Berger, C., Berger, M., Feng, J. & Riddell, S.R. Genetic modification of T cells for immunotherapy. *Expert Opin Biol Ther* **7**, 1167-1182 (2007).

29. Bobisse, S., Zanovello, P. & Rosato, A. T-cell receptor gene transfer by lentiviral vectors in adoptive cell therapy. *Expert Opin Biol Ther* **7**, 893-906 (2007).

30. Brenner, M.K. & Heslop, H.E. Adoptive T cell therapy of cancer. *Curr Opin Immunol* **22**, 251-257 (2010).

31. Eshhar, Z. Adoptive cancer immunotherapy using genetically engineered designer T-cells: First steps into the clinic. *Curr Opin Mol Ther* **12**, 55-63 (2010).

32. Kieback, E. & Uckert, W. Enhanced T cell receptor gene therapy for cancer. *Expert Opin Biol Ther* **10**, 749-762 (2010).

33. Marcu-Malina, V., van Dorp, S. & Kuball, J. Re-targeting T-cells against cancer by gene-transfer of tumor-reactive receptors. *Expert Opin Biol Ther* **9**, 579-591 (2009).

34. Morgan, R.A., Dudley, M.E. & Rosenberg, S.A. Adoptive cell therapy: genetic modification to redirect effector cell specificity. *Cancer J* **16**, 336-341 (2010).

35. Riddell, S.R. Engineering antitumor immunity by T-cell adoptive immunotherapy. *Hematology Am Soc Hematol Educ Program* **2007**, 250-256 (2007).

36. Sadelain, M., Brentjens, R. & Riviere, I. The promise and potential pitfalls of chimeric antigen receptors. *Curr Opin Immunol* **21**, 215-223 (2009).

37. Thomas, S., Hart, D.P., Xue, S.A., Cesco-Gaspere, M. & Stauss, H.J. T-cell receptor gene therapy for cancer: the progress to date and future objectives. *Expert Opin Biol Ther* **7**, 1207-1218 (2007).

38. Rosenberg, S.A. & Dudley, M.E. Adoptive cell therapy for the treatment of patients with metastatic melanoma. *Curr Opin Immunol* **21**, 233-240 (2009).

39. Jena, B., Dotti, G. & Cooper, L.J. Redirecting T-cell specificity by introducing a tumor-specific chimeric antigen receptor. *Blood* **116**, 1035-1044 (2010).

40. Dudley, M.E., *et al.* Adoptive cell transfer therapy following non-myeloablative but lymphodepleting chemotherapy for the treatment of patients with refractory metastatic melanoma. *J Clin Oncol* **23**, 2346-2357 (2005).

41. Rosenberg, S.A. & Dudley, M.E. Cancer regression in patients with metastatic melanoma after the transfer of autologous antitumor lymphocytes. *Proc Natl Acad Sci U S A* **101 Suppl 2**, 14639-14645 (2004).

42. Appay, V., *et al.* Memory CD8+ T cells vary in differentiation phenotype in different persistent virus infections. *Nat Med* **8**, 379-385 (2002).

43. Joshi, N.S., *et al.* Inflammation directs memory precursor and short-lived effector CD8(+) T cell fates via the graded expression of T-bet transcription factor. *Immunity* **27**, 281-295 (2007).

44. Gattinoni, L., *et al.* Acquisition of full effector function in vitro paradoxically impairs the in vivo antitumor efficacy of adoptively transferred CD8+ T cells. *J Clin Invest* **115**, 1616-1626 (2005).

45. Kaech, S.M. & Wherry, E.J. Heterogeneity and cell-fate decisions in effector and memory CD8+ T cell differentiation during viral infection. *Immunity* **27**, 393-405 (2007).

46. Sallusto, F., Geginat, J. & Lanzavecchia, A. Central memory and effector memory T cell subsets: function, generation, and maintenance. *Annu Rev Immunol* **22**, 745-763 (2004).

47. Cooper, L.J., *et al.* T-cell genetic modification for re-directed tumor recognition. *Cancer Chemother Biol Response Modif* **22**, 293-324 (2005).

48. Kunwar, S., *et al.* Direct intracerebral delivery of cintredekin besudotox (IL13-PE38QQR) in recurrent malignant glioma: a report by the Cintredekin Besudotox Intraparenchymal Study Group. *J Clin Oncol* **25**, 837-844 (2007).

49. Strohl, W.R. Optimization of Fc-mediated effector functions of monoclonal antibodies. *Curr Opin Biotechnol* **20**, 685-691 (2009).

50. Kowolik, C.M., *et al.* CD28 costimulation provided through a CD19-specific chimeric antigen receptor enhances in vivo persistence and antitumor efficacy of adoptively transferred T cells. *Cancer Res* **66**, 10995-11004 (2006).

51. Wang, J., *et al.* Optimizing adoptive polyclonal T cell immunotherapy of lymphomas, using a chimeric T cell receptor possessing CD28 and CD137 costimulatory domains. *Hum Gene Ther* **18**, 712-725 (2007).

52. Croft, M. Costimulation of T cells by OX40, 4-1BB, and CD27. *Cytokine Growth Factor Rev* **14**, 265-273 (2003).

53. Duttagupta, P.A., Boesteanu, A.C. & Katsikis, P.D. Costimulation signals for memory CD8+ T cells during viral infections. *Critical reviews in immunology* **29**, 469-486 (2009).

54. Grupp, S.A., *et al.* Chimeric Antigen Receptor-Modified T Cells for Acute Lymphoid Leukemia. *N Engl J Med* **368**, 1509-1518 (2013).

55. Kalos, M., *et al.* T cells with chimeric antigen receptors have potent antitumor effects and can establish memory in patients with advanced leukemia. *Sci Transl Med* **3**, 95ra73 (2011).

56. Till, B.G., *et al.* CD20-specific adoptive immunotherapy for lymphoma using a chimeric antigen receptor with both CD28 and 4-1BB domains: pilot clinical trial results. *Blood* **119**, 3940-3950 (2012).

57. Einsele, H., *et al.* Infusion of cytomegalovirus (CMV)-specific T cells for the treatment of CMV infection not responding to antiviral chemotherapy. *Blood* **99**, 3916-3922 (2002).

58. Moeller, M., *et al.* Adoptive transfer of gene-engineered CD4+ helper T cells induces potent primary and secondary tumor rejection. *Blood* **106**, 2995-3003 (2005).

59. Walker, R.E., *et al.* Long-term in vivo survival of receptor-modified syngeneic T cells in patients with human immunodeficiency virus infection. *Blood* **96**, 467-474 (2000).
60. Kohn, D.B., *et al.* CARs on track in the clinic. *Mol Ther* **19**, 432-438 (2011).
61. Vence, L., *et al.* Circulating tumor antigen-specific regulatory T cells in patients with metastatic melanoma. *Proc Natl Acad Sci U S A* **104**, 20884-20889 (2007).
62. Powell, D.J., Jr., Parker, L.L. & Rosenberg, S.A. Large-scale depletion of CD25+ regulatory T cells from patient leukapheresis samples. *J Immunother* **28**, 403-411 (2005).
63. Klebanoff, C.A., Gattinoni, L. & Restifo, N.P. Sorting through subsets: which T-cell populations mediate highly effective adoptive immunotherapy? *J Immunother* **35**, 651-660 (2012).
64. Wang, X., *et al.* Phenotypic and Functional Attributes of Lentivirus-modified CD19-specific Human CD8+ Central Memory T Cells Manufactured at Clinical Scale. *J Immunother* **35**, 689-701 (2012).
65. Wang, X., *et al.* A transgene-encoded cell surface polypeptide for selection, in vivo tracking, and ablation of engineered cells. *Blood* **118**, 1255-1263 (2011).
66. Brown, C.E., *et al.* Recognition and killing of brain tumor stem-like initiating cells by CD8+ cytolytic T cells. *Cancer Res* **69**, 8886-8893 (2009).
67. Jensen, M.C., *et al.* Antitransgene rejection responses contribute to attenuated persistence of adoptively transferred CD20/CD19-specific chimeric antigen receptor redirected T cells in humans. *Biol Blood Marrow Transplant* **16**, 1245-1256 (2010).
68. Park, J.R., *et al.* Adoptive transfer of chimeric antigen receptor re-directed cytolytic T lymphocyte clones in patients with neuroblastoma. *Mol Ther* **15**, 825-833 (2007).
69. Donnelly, M.L., *et al.* Analysis of the aphthovirus 2A/2B polyprotein 'cleavage' mechanism indicates not a proteolytic reaction, but a novel translational effect: a putative ribosomal 'skip'. *J Gen Virol* **82**, 1013-1025 (2001).
70. Miyoshi, H., Blomer, U., Takahashi, M., Gage, F.H. & Verma, I.M. Development of a self-inactivating lentivirus vector. *J Virol* **72**, 8150-8157 (1998).
71. Lee, D.W., *et al.* Current concepts in the diagnosis and management of cytokine release syndrome. *Blood* **124**, 188-195 (2014).
72. Davila, M.L., *et al.* Efficacy and Toxicity Management of 19-28z CAR T Cell Therapy in B Cell Acute Lymphoblastic Leukemia. *Sci Transl Med* **6**, 224ra225 (2014).
73. Kumar, R., *et al.* Th1/Th2 cytokine imbalance in meningioma, anaplastic astrocytoma and glioblastoma multiforme patients. *Oncol Rep* **15**, 1513-1516 (2006).
74. Lippitz, B.E. Cytokine patterns in patients with cancer: a systematic review. *Lancet Oncol* **14**, e218-228 (2013).
75. Kochenderfer, J.N., *et al.* B-cell depletion and remissions of malignancy along with cytokine-associated toxicity in a clinical trial of anti-CD19 chimeric-antigen-receptor-transduced T cells. *Blood* **119**, 2709-2720 (2012).
76. Adair, J.E., *et al.* Gene therapy enhances chemotherapy tolerance and efficacy in glioblastoma patients. *J Clin Invest* **124**, 4082-4092 (2014).
77. Baldock, A.L., *et al.* Patient-specific metrics of invasiveness reveal significant prognostic benefit of resection in a predictable subset of gliomas. *PLoS One* **9**, e99057 (2014).
78. Baldock, A.L., *et al.* From patient-specific mathematical neuro-oncology to precision medicine. *Frontiers in oncology* **3**, 62 (2013).

79. Smith, T.R., *et al.* Survival after surgery and stereotactic radiosurgery for patients with multiple intracranial metastases: results of a single-center retrospective study. *J Neurosurg* **121**, 839-845 (2014).

16.0 Appendices

16.1 APPENDIX A – Toxicity Grading

NCI Common Toxicity Criteria V.4.0

Toxicity data being obtained at each clinical assessment will be graded using NCI Common Toxicity Criteria V.4.0 which may be downloaded from the NCI website (*http://evs.nci.nih.gov/ftp1/CTCAE/*)

Any toxicity reported by research participants while receiving treatment or in follow-up for which there is no specific CTC designation will be graded on the following scale:

Table 1: Grading for Toxicities with no specific CTC designation

Grade of toxicity	Toxicity Description
0	No toxicity
1	Mild toxicity, usually transient, requiring no special treatment and generally not interfering with usual daily activities
2	Moderate toxicity that may be ameliorated by simple therapeutic maneuvers, and does not impair usual activities
3	Severe toxicity which requires therapeutic intervention and interrupts usual activities, hospitalization may be required or may not be required
4	Life-threatening toxicity which requires hospitalization

16.2 APPENDIX B – Response Criteria and Grading

16.2.1 Response Assessment in Neuro-Oncology (RANO) criteria (cited below).

- *Complete Response (CR)*: Complete disappearance of all enhancing disease (measurable and non-measurable) that is sustained for at least 4 weeks, stable or improved non-enhancing FLAIR/T2 lesions, no new lesions, off corticosteroids (physiologic replacement doses allowed), and neurologically stable or improved.
- *Partial Response (PR)*: $\geq$ 50% decrease of all measurable enhancing lesions, sustained for at least 4 weeks, no progression of non-measurable disease, stable or improved non-enhancing FLAIR/T2 lesions, no new lesions, corticosteroid dose stable or reduced (compared to baseline), and neurologically stable or improved.
- *Stable Disease (SD)*: Does not qualify for CR, PR or PD, stable non-enhancing FLAIR/T2 lesions, stable or reduced corticosteroids (compared to baseline), clinically stable.
- *Progressive Disease (PD)*: $\geq$ 25% increase in enhancing lesions despite stable or increasing steroid dose, increase (significant) in non-enhancing FLAIR/T2 lesions that is not attributable to other non-tumor causes, any new lesions, clinical deteriorations (not attributable to other non-tumor causes and not due to steroid decrease).

16.2.2 Retrospective Research Analysis - Mathematical Models of Tumor Invasion and Proliferation

Dr. Rockne's Laboratory here at City of Hope, in collaboration with Dr. Swanson's Laboratory at Mayo Clinic, will assess MRI and PET images pre and post T cell therapy to evaluate the impact of the therapy on tumor progression and survival outcomes. Dr. Rockne was a member of the Swanson lab, previously at Northwestern University, where their lab devised mathematical models of tumor invasion and proliferation that predict patient outcomes, and has an extensive image and survival database of glioma patients receiving standard of care treatments.[76-79] Thus, Dr. Rockne, in collaboration with Dr. Swanson, will be able to assess therapeutic responses post therapy compared to a set of comparable recurrent glioblastoma patients who received standard of care treatments to infer potential therapeutic impact of the gene-modified T cells in this subject. Any data/records shared with Dr. Swanson's Laboratory at Mayo Clinic will be de-identified.

16.3 APPENDIX C: Cytokine Release Syndrome (CRS) in the Context of Cellular Immunotherapy (Clinical Symptoms & Revised Grading System)

16.3.1 Clinical signs and symptoms associated with CRS

Organ System	Symptoms
Constitutional	Fever ± rigors, malaise, fatigue, anorexia, myalgias, arthalgias, nausea, vomiting, headache
Skin	Rash
Gastrointestinal	Nausea, vomiting, diarrhea
Respiratory	Tachypnea, hypoxemia
Cardiovascular	Tachycardia, widened pulse pressure, hypotension, increased cardiac output (early), potentially diminished cardiac output (late)
Coagulation	Elevated D-dimer, hypofibrinogenemia ± bleeding
Renal	Azotemia
Hepatic	Transaminitis, hyperbilirubinemia
Neurologic	Headache, mental status changes, confusion, delirium, word finding difficulty or frank aphasia, hallucinations, tremor, dymetria, altered gait, seizures

16.3.2 Revised CRS Grading System

Grade	Toxicity
Grade 1	Symptoms are not life threatening and require symptomatic treatment only; e.g: fever, nausea, fatigue, headache, myalgias, malaise
Grade 2	Symptoms require and respond to moderate intervention
	Oxygen requirement <40% or
	Hypotension responsive to fluids or low dose of one vasopressor or
	Grade 2 organ toxicity*
Grade 3	Symptoms require and respond to aggressive intervention
	Oxygen requirement ≥40% or
	Hypotension requiring high dose or multiple vasopressors or
	Grade 3 organ toxicity* or grade 4 transaminitis
Grade 4	Life-threatening symptoms
	Requirement for ventilator support or
	Grade 4 organ toxicity* (excluding transaminitis)
Grade 5	Death

*Grades 2-4 refer to CTCAE v4.0 grading

16.3.3 High-dose vasopressors (all doses are required for > 3 hours)

Pressor	Dose
Norepinephrine monotherapy	≥20 µg /min
Dopamine monotherapy	≥10 µg/kg/min
Phenylephrine monotherapy	≥200 µg/min
Epinephrine monotherapy	≥10 µg/min
If on vasopressin	Vasopressin + norepinephrine equivalent of ≥10 µg/min*
If on combination vasopressors (not vasopressin)	Norepinephrine equivalent of ≥20 µg/min*

* VASST Trial vasopressor equivalent equation: norepinephrine equivalent dose = [norepinephrine (µg/min)] + [dopamine (µg/kg/min) ÷ 2] + [epinephrine (µg/min)] + [phenylephrine (µg/min) ÷10].

16.4 APPENDIX D – Case Report Forms

Case Report Forms

To be determined

Medidata Forms (eCRFs)

All the eCRFs are accessible via Medidata Rave at https://coh.mdsol.com/MedidataRAVE/%28k444s3nsk0uw0b55z5d1zzff%29/Login.aspx
