# Supplementary material for: Locoregional delivery of IL-13Rα2-targeting CAR-T cells in recurrent high-grade glioma: a phase 1 trial
Source: Nat Med. 2024 Mar 7;30(4):1001–12. doi: 10.1038/s41591-024-02875-1 (PMC11031404; doi:10.1038/s41591-024-02875-1)
Supplement: Supplementary file 2 — Reporting Summary [file 41591_2024_2875_MOESM2_ESM.pdf]

nature portfolio

Corresponding author(s): Christine Brown

Last updated by author(s): Feb 13, 2024

Reporting Summary

Nature Portfolio wishes to improve the reproducibility of the work that we publish. This form provides structure for consistency and transparency in reporting. For further information on Nature Portfolio policies, see our Editorial Policies and the Editorial Policy Checklist.

Statistics

For all statistical analyses, confirm that the following items are present in the figure legend, table legend, main text, or Methods section.

n/a	Confirmed	
☐	☒	The exact sample size (n) for each experimental group/condition, given as a discrete number and unit of measurement
☐	☒	A statement on whether measurements were taken from distinct samples or whether the same sample was measured repeatedly
☐	☒	The statistical test(s) used AND whether they are one- or two-sided<br>*Only common tests should be described solely by name; describe more complex techniques in the Methods section.*
☐	☒	A description of all covariates tested
☐	☒	A description of any assumptions or corrections, such as tests of normality and adjustment for multiple comparisons
☐	☒	A full description of the statistical parameters including central tendency (e.g. means) or other basic estimates (e.g. regression coefficient) AND variation (e.g. standard deviation) or associated estimates of uncertainty (e.g. confidence intervals)
☐	☒	For null hypothesis testing, the test statistic (e.g. F, t, r) with confidence intervals, effect sizes, degrees of freedom and P value noted<br>*Give P values as exact values whenever suitable.*
☒	☐	For Bayesian analysis, information on the choice of priors and Markov chain Monte Carlo settings
☒	☐	For hierarchical and complex designs, identification of the appropriate level for tests and full reporting of outcomes
☐	☒	Estimates of effect sizes (e.g. Cohen's d, Pearson's r), indicating how they were calculated

Our web collection on statistics for biologists contains articles on many of the points above.

Software and code

Policy information about availability of computer code

Data collection	Siemens MAGNETOM Verio 3.0 Tesla scanner; GE Discovery DST HP60 PET-CT scanner; NanoZoomer 2.0-HT digital slide scanner; NanoZoomer S360 Digital Slide Scanner; Olympus BX46 transmitted light microscope with an SC-180 Olympus camera; MACSQuant Analyzer 10; FLEXMAP 3D® (Luminex); TaqMan qPCR; Illumina Iseq100; IlluminaNovaSeq6000.
Data analysis	Vital Images Vitrea v6.7.2; ITK-SNAP v3.8.0; FlowJo v10.1; GraphPad Prism v9; 10x Genomics Cell Ranger v5.0 and Ensemble 98; Seurat v4; R v4.0.2; Visiopharm v2023.01; Demuxlet; BWA v0.7.17-r1188; Samtools Fixmates and Samtools Sort v1.10; DeepVariant (https://github.com/google/deepvariant).<br>Scripts to conduct the RNAseq analyses are publicly available on GitHub at https://github.com/Banovich-Lab/13384_IL13Ra2_CART_product

For manuscripts utilizing custom algorithms or software that are central to the research but not yet described in published literature, software must be made available to editors and reviewers. We strongly encourage code deposition in a community repository (e.g. GitHub). See the Nature Portfolio guidelines for submitting code & software for further information.

Data

Policy information about availability of data

All manuscripts must include a data availability statement. This statement should provide the following information, where applicable:

- Accession codes, unique identifiers, or web links for publicly available datasets
- A description of any restrictions on data availability
- For clinical datasets or third party data, please ensure that the statement adheres to our policy

AAll requests for raw and analyzed data and materials will be promptly reviewed by the corresponding author and appropriate COH committees to verify if the request is subject to any intellectual property or confidentiality obligations. Requests may be made to cbrown@coh.org; response time will be within approximately 30 business days. Release of individual-level data may be restricted due to patient confidentiality considerations. Any data and materials that can be shared will be de-identified and released via a data or material transfer agreement. The RNA-sequencing data is deposited on the National Center of Biotechnology Information Gene Expression Omnibus (NCBI GEO) under the accession number GSE255850.

Research involving human participants, their data, or biological material

Policy information about studies with human participants or human data. See also policy information about sex, gender (identity/presentation), and sexual orientation and race, ethnicity and racism.

Reporting on sex and gender	This information is provided in Table 1 and Extended Table 1. Sex or gender was not considered in the study design as rHGG occurs in both males and females.
Reporting on race, ethnicity, or other socially relevant groupings	This information is provided in Extended Table 1.
Population characteristics	This information is provided in Table 1 and Extended Table 1.
Recruitment	Research participants were identified at City of Hope through the clinical practices of the PI, co-Is and participating clinicians and through direct referrals from outside hospitals and physicians. No bias emerging from recruitment is expected. Patients were not compensated for their participation on the study.
Ethics oversight	This study was conducted in accordance with the Institutional Review Board, Data Safety Monitoring Committee and Independent Ethics Committee at The City of Hope (COH) National Medical Center as well as the U.S. Food and Drug Administration. All subjects provided written informed consent in accordance with local regulatory review.

Note that full information on the approval of the study protocol must also be provided in the manuscript.

Field-specific reporting

Please select the one below that is the best fit for your research. If you are not sure, read the appropriate sections before making your selection.

☒ Life sciences ☐ Behavioural & social sciences ☐ Ecological, evolutionary & environmental sciences

For a reference copy of the document with all sections, see nature.com/documents/nr-reporting-summary-flat.pdf

Life sciences study design

All studies must disclose on these points even when the disclosure is negative.

Sample size	Based on simulation results, we expected to study 15 participants in the dose escalation portion of the trial, plus 6 in the expansion portion plus 2 for replacement of unevaluable participants, giving an expected sample size of 23 per Arm for each of the 4 open Arms for a total of 92. In actuality we achieved this exact sample size (reference Fig. 1c).<br>For secondary objectives, sample sizes depended on availability of tumor, CSF, TCF and PB samples.
Data exclusions	Study participants that did not receive the full schedule of 3 T cell doses were excluded from analysis; this was pre-established in the clinical protocol.
Replication	Statistical tests were employed to ensure significance of results within our study. However the study was limited to a single site, and investigation true replicability is beyond the scope of the study.
Randomization	This was a nonrandomized study. The first 4 research participants in Arms 1- 3 were treated sequentially, followed through the DLT period (3 infusion cycles plus 1-week for AE evaluations) before the next participant could receive their initial infusion; all further research participants were treated in cohorts of 3. The first research participant in Arms 4 and 5 were followed through the DLT period before the other 2 participants in that cohort were treated, all further participants followed in cohorts of 3.
Blinding	Analysts used a de-identified dataset. However blinding was not possible for clinicians due to nature of the clinical interventions. As noted above, the study was not a randomized study.

Reporting for specific materials, systems and methods

We require information from authors about some types of materials, experimental systems and methods used in many studies. Here, indicate whether each material, system or method listed is relevant to your study. If you are not sure if a list item applies to your research, read the appropriate section before selecting a response.

Materials & experimental systems

n/a	Involved in the study
☐	☒ Antibodies
☐	☒ Eukaryotic cell lines
☒	☐ Palaeontology and archaeology
☐	☒ Animals and other organisms
☐	☒ Clinical data
☒	☐ Dual use research of concern
☒	☐ Plants

Methods

n/a	Involved in the study
☒	☐ ChIP-seq
☐	☒ Flow cytometry
☐	☒ MRI-based neuroimaging

Antibodies

Antibodies used

1) CCR7-PE (R&D Systems Cat. # FAB197P, Clone 150503)
2) CD3 (Leica Biosystems Cat. # PA0553, Clone LN10)
3) CD3-APC (BD Biosciences Cat. # 340440, Clone SK7)
4) CD3-VioGreen (BD Biosciences Cat. # 563109, Clone UCHT1)
5) CD4 (Ventana Cat. # 790-4423, Clone SP35)
6) CD4-FITC (BD Biosciences Cat. # 340133, Clone SK3)
7) CD4-PerCP (BD Biosciences Cat. # 347324, Clone SK3)
8) CD8 (Ventana Cat # 790-4460, Clone SP57)
9) CD8-APC-Cy7 (BD Biosciences Cat. # 348793, Clone SK1)
10) CD19-PE-Cy7 (BD Biosciences Cat. # 557835, Clone SJ25C1)
11) CD25-APC-Cy7 (BioLegend Cat # 302613, Clone BC96)
12) CD27-APC-Cy7 (BioLegend Cat. #302816, Clone O323)
13) CD27-PE (BD Pharmingen Cat. # 555441, Clone M-T271)
14) CD45RA-FITC (BD Bioscience Cat. # 555488, Clone HI100)
15) CD57-FITC (BD Biosciences Cat. # 555619, Clone NK-1)
16) CD62L-FITC (BD Biosciences Cat. # 347443, SK11)
17) CD62L-PE (BD Biosciences Cat. # 341012, Clone SK11)
18) CD66b (Novus Cat. # NB100-77808, Clone G105F)
19) CD68 (Ventana Cat. # 790-2931, Clone KP-1)
20) FOXP3 (Abcam Cat. # ab20034, Clone 236A/E7)
21) FOXP3-PE (eBiosciences Cat. # 12-4777-42, Clone 236A/E7)
22) IL13Rα2 (Cell Signaling Technology Cat. # 85677, Clone E7U7B)
23) LAG-3-PE (eBiosciences Cat. # 12-2239-42, Clone 3DS223H)
24) LAG-3-FITC (Lifespan Biosciences Cat. # LS-C344745, Clone 17B4)
25) PD-1-FITC (eBiosciences Cat. # 11-9969-42, Clone MIH4)
26) PD-1-PE (eBiosciences Cat. # 12-9969-42, Clone MIH4)
27) DISCOVERY anti-Rabbit HQ (Ventana Cat. # 760-4815)
28) DISCOVERY anti-Rabbit NP (Ventana Cat. # 760-4817)
29) DISCOVERY anti-Mouse HQ (Ventana Cat. # 760-4814)
30) DISCOVERY anti-Mouse NP (Ventana Cat. # 760-4816)
31) DISCOVERY anti-NP-AP (Ventana Cat. # 760-4827)
32) DISCOVERY anti-HQ-HRP (Ventana Cat. # 760-4820)

The concentration of antibody used is the one recommended by the manufacturer.
Lot number information was not recorded.

Validation

All antibodies are validated by specificity to their respective target on human cells as provided by the manufacturer's information and references available in the following websites:
1) https://www.rndsystems.com/products/human-ccr7-pe-conjugated-antibody-150503_fab197p
2) https://shop.leicabiosystems.com/us/actions/ViewProductAttachment-OpenFile?LocaleId=&DirectoryPath=SDSs&FileName=pa0553.pdf&UnitName=LBS
3) https://www.bdbiosciences.com/en-us/products/reagents/flow-cytometry-reagents/clinical-discovery-research/single-color-antibodies-ruo-gmp/apc-mouse-anti-human-cd3.340440
4) https://www.bdbiosciences.com/en-us/products/reagents/flow-cytometry-reagents/research-reagents/single-color-antibodies-ruo/bv510-mouse-anti-human-cd3.563109
5) https://diagnostics.roche.com/us/en/products/lab/cd4-sp35-confirm-rtd000770.html
6) https://www.bdbiosciences.com/en-us/products/reagents/flow-cytometry-reagents/clinical-diagnostics/single-color-antibodies-asr-ivd-ce-ivd/cd4-fitc.340133
7) https://www.bdbiosciences.com/en-us/products/reagents/flow-cytometry-reagents/clinical-discovery-research/single-color-antibodies-ruo-gmp/percp-mouse-anti-human-cd4.347324

8) https://diagnostics.roche.com/us/en/products/lab/cd8-sp57-confirm-rtd000777.html
9) https://www.bdbiosciences.com/en-us/products/reagents/flow-cytometry-reagents/clinical-discovery-research/single-color-antibodies-ruo-gmp/apc-cy-7-mouse-anti-human-cd8.348793
10) https://www.bdbiosciences.com/en-us/products/reagents/flow-cytometry-reagents/research-reagents/single-color-antibodies-ruo/pe-cy-7-mouse-anti-human-cd19.557835
11) https://www.biolegend.com/fr-ch/products/apc-cyanine7-anti-human-cd25-antibody-1908?GroupID=BLG7919
12) https://www.biolegend.com/en-us/products/apc-cyanine7-anti-human-cd27-antibody-3611
13) https://www.bdbiosciences.com/en-us/products/reagents/flow-cytometry-reagents/research-reagents/single-color-antibodies-ruo/pe-mouse-anti-human-cd27.555441
14) https://www.bdbiosciences.com/en-us/products/reagents/flow-cytometry-reagents/research-reagents/single-color-antibodies-ruo/fitc-mouse-anti-human-cd45ra.555488
15) https://www.bdbiosciences.com/en-eu/products/reagents/flow-cytometry-reagents/research-reagents/single-color-antibodies-ruo/fitc-mouse-anti-human-cd57.555619
16) https://www.bdbiosciences.com/en-us/products/reagents/flow-cytometry-reagents/clinical-discovery-research/single-color-antibodies-ruo-gmp/fitc-mouse-anti-human-cd62l.347443
17) https://www.bdbiosciences.com/en-us/products/reagents/flow-cytometry-reagents/clinical-discovery-research/single-color-antibodies-ruo-gmp/pe-mouse-anti-human-cd62l.341012
18) https://www.novusbio.com/products/ceacam8-cd66b-antibody-g10f5_nb100-77808
19) https://diagnostics.roche.com/global/en/products/lab/cd68-kp-1-confirm-rtd000775.html
20) https://www.abcam.com/products/primary-antibodies/foxp3-antibody-236ae7-ab20034.html
21) https://www.thermofisher.com/antibody/product/FOXP3-Antibody-clone-236A-E7-Monoclonal/12-4777-42
22) https://www.cellsignal.com/products/primary-antibodies/il-13ra2-cd213a2-e7u7b-rabbit-mab/85677
23) https://www.thermofisher.com/antibody/product/CD223-LAG-3-Antibody-clone-3DS223H-Monoclonal/17-2239-42
24) https://www.lsbio.com/antibodies/lag3-antibody-aa70-99-clone-17b4-fitc-flow-icc-ihc-ip-wb-western-ls-c344745/355623
25) https://www.thermofisher.com/antibody/product/CD279-PD-1-Antibody-clone-MIH4-Monoclonal/11-9969-42
26) https://www.thermofisher.com/antibody/product/CD279-PD-1-Antibody-clone-MIH4-Monoclonal/12-9969-42
27) https://elabdoc-prod.roche.com/eLD/web/us/en/documents/download/0c64b3ff-2636-ea11-fc90-005056a71a5d
28) https://elabdoc-prod.roche.com/eLD/web/us/en/documents/download/3997c013-8957-eb11-0291-005056a71a5d
29) https://elabdoc-prod.roche.com/eLD/web/us/en/documents/download/4f5ab3ff-2636-ea11-fc90-005056a71a5d
30) https://elabdoc-prod.roche.com/eLD/web/us/en/documents/download/e1c99c27-7857-eb11-0091-005056a772fd
31) https://elabdoc-prod.roche.com/eLD/web/us/en/documents/download/16a84d11-8957-eb11-0291-005056a71a5d
32) https://elabdoc-prod.roche.com/eLD/web/us/en/documents/download/1aa72a08-2736-ea11-fc90-005056a71a5d

Eukaryotic cell lines

Policy information about cell lines and Sex and Gender in Research

Cell line source(s)	Raji-ffluc was a kind gift from Dr. Michael Jensen in 2014; patient-derived glioma line PBT030-2-ffluc-IL13Rα2+ was generated at COH
Authentication	All tumor lines were authenticated for the desired antigen/marker expression by flow cytometry, tested for mycoplasma using the MycoAlert™ PLUS Mycoplasma Detection Kit (Lonza), and maintained in culture for less than 1-2 months.
Mycoplasma contamination	All cell lines tested negative for mycoplasma contamination
Commonly misidentified lines (See ICLAC register)	No commonly misidentified lines were used in this study.

Animals and other research organisms

Policy information about studies involving animals; ARRIVE guidelines recommended for reporting animal research, and Sex and Gender in Research

Laboratory animals	NOD/Scid IL2RγCnull (NSG) mice; 9-10 weeks-old
Wild animals	Study did not involve wild animals
Reporting on sex	Findings do not apply to one sex as rHGG occurs in both males and females.
Field-collected samples	Study did not involve samples collected from the field
Ethics oversight	All animal studies were approved by the City of Hope Institutional Animal Care and Use Committee (IACUC).

Note that full information on the approval of the study protocol must also be provided in the manuscript.

Clinical data

Policy information about clinical studies
All manuscripts should comply with the ICMJE guidelines for publication of clinical research and a completed CONSORT checklist must be included with all submissions.

Clinical trial registration	NCT02208362

Study protocol	Full trial protocol is available as Supplementary Information
Data collection	Patients were enrolled and treated on this phase I study between June 2015 and February 2021 at City of Hope Beckman Research Institute and Medical Center in Duarte, CA 91010
Outcomes	The Primary Objectives were:<br>• Assess the feasibility and safety of cellular immunotherapy utilizing ex vivo expanded autologous memory-enriched T cells that are genetically modified using a self-inactivating (SIN) lentiviral vector to express a IL13Rα2-specific, hinge-optimized, 41BB-costimulatory CAR, as well as a truncated human CD19 for participants with recurrent/refractory malignant glioma in one of the following ways: Arm 1 (intratumoral delivery of IL13(EQ)BBζ/CD19t+ Tcm), Arm 2 (intracavitary delivery of IL13(EQ)BBζ/CD19t+ Tcm), Arm 3 (intraventricular delivery of IL13(EQ)BBζ/CD19t+ Tcm), Arm 4 (dual delivery [both intratumoral and intraventricular] of IL13(EQ)BBζ/CD19t+ Tcm) or Arm 5 (dual delivery [both intratumoral and intraventricular] of IL13(EQ)BBζ/CD19t+ Tn/mem), and<br>• Determine maximum tolerated dose schedule (MTD)/maximum feasible dose schedule (MFD) and a recommend Phase II dosing plan (RP2D) for each arm based on dose limiting toxicities (DLTs) and the full toxicity profile.<br><br>The Secondary Objectives are<br>• In research participants who receive the full schedule of 3 CAR T cell doses:<br>o Estimate disease response rates,<br>o Estimate median overall survival, and<br>o Estimate the mean change from baseline in quality of life using the EORTC QLQ-C30 during and post treatment;<br>• Describe cytokine levels (CSF, tumor cavity fluid, peripheral blood) over the study period;<br>• Describe CAR T cell and endogenous immune populations (CSF, tumor cavity fluid, peripheral blood) over the study period; and<br>• Identify tumor and tumor micro-environment markers associated with response to CAR T cells.

Flow Cytometry

Plots

Confirm that:

☒ The axis labels state the marker and fluorochrome used (e.g. CD4-FITC).

☒ The axis scales are clearly visible. Include numbers along axes only for bottom left plot of group (a 'group' is an analysis of identical markers).

☒ All plots are contour plots with outliers or pseudocolor plots.

☒ A numerical value for number of cells or percentage (with statistics) is provided.

Methodology

Sample preparation	Cell suspensions were washed and resuspended in PBS.
Instrument	MACSQuant Analyzer 10 (Miltenyi Biotec)
Software	FlowJo software (v10.1, TreeStar) and GraphPad Prism Software (v9).
Cell population abundance	Refer to histograms/percentages on figures.
Gating strategy	Gating strategies are depicted in Supplementary Figure 2. In brief, PBMC or CAR-T cell products were gated first to exclude debris by FSC vs SSC, then for viability using DAPI, and, when indicated, further for the CD3+ population. For Treg analyses, CAR-T cell products were gated first for viability, then for the CD3+ population, then for the CD4+ population, and finally for the CAR/CD19t+ population. Cells in the CSF/TCF were gated first to exclude debris by FSC vs SSC, then for viability using DAPI, and then further for the CD3+ population.

☒ Tick this box to confirm that a figure exemplifying the gating strategy is provided in the Supplementary Information.

Magnetic resonance imaging

Experimental design

Design type	Response Assessment in Neuro-Oncology (RANO) version 1 criteria
Design specifications	N/A - not a functional assessment
Behavioral performance measures	N/A - not a functional assessment

Acquisition

Imaging type(s): Structural, Diffusion-Weighted (for clinical use, not volumetric or functional analysis, not reported)

Field strength: 3 Tesla

Sequence & imaging parameters: T1-weighted with and without macrocyclic Gd-based contrast agents, T2-weighted, T2-FLuid Attenuation Inversion Recovery (FLAIR), Diffusion-Weighted Imaging (DWI). Gradient-based sequences, and others, as clinically/technically necessary

Area of acquisition: Whole brain

Diffusion MRI: ☒ Used ☐ Not used

Parameters: as necessary for optimal image acquisition, assessment, or surgical planning; up to 20 directions for tractography, when needed, minimally: b-1000, b-0, no cardiac gating

Preprocessing

Preprocessing software: BraTumIA v 2.0.0.5 for registration, normalization, and resampling. Skull-stripped images were not used. ITK-SNAP v 3.8 was used for volume segmentation of Enhancing Tumor and Edema Volumes of Interest.

Normalization: BraTumIA employs a linear transformation technique to register the images.

Normalization template: Images are registered to the T1-weighted sequence using a rigid registration technique based on mutual information.

Noise and artifact removal: BraTumIA uses an edge-preserving smoothing filter and corrects for field bias.

Volume censoring: N/A

Statistical modeling & inference

Model type and settings: N/A - volumes are not used for modeling

Effect(s) tested: N/A

Specify type of analysis: ☐ Whole brain ☒ ROI-based ☐ Both

Anatomical location(s): Using the 4 structural images, BraTumIA generates an algorithmically determined labeling of Clear Label, CSF, White Matter, Grey Matter, Edema, Necrosis, and Enhancing Tumor. None of these labels were reported or used for determining change in volumes. Labeling for Edema and Enhancing tumor was conducted manually on the co-registered images.

Statistic type for inference (See Eklund et al. 2016): Voxel count and Volume are calculated within ITK-SNAP using the affine and other metadata included in the image file

Correction: N/A

Models & analysis

n/a	Involved in the study
☒	☐ Functional and/or effective connectivity
☒	☐ Graph analysis
☒	☐ Multivariate modeling or predictive analysis
